# Lung cancer and diesel exhaust: an updated critical review of the occupational epidemiology literature

**DOI:** 10.3109/10408444.2012.690725

**Published:** 2012-06-02

**Authors:** John F. Gamble, Mark J. Nicolich, Paolo Boffetta

**Affiliations:** 1 566 Elizabeth Avenue, Somerset, NJ 08873, USA; 2 COGIMET, Lambertville, NJ, USA; 3 The Tisch Cancer Institute and Institute for Translational Epidemiology, Mount Sinai School of Medicine in New York, NY, USA; 4 International Prevention research Institute, Lyon, France

**Keywords:** Cumulative exposure, diesel exhaust, elemental carbon, epidemiology, exposure-response, latency, lung cancer, odds ratio

## Abstract

A recent review concluded that the evidence from epidemiology studies was indeterminate and that additional studies were required to support the diesel exhaust-lung cancer hypothesis. This updated review includes seven recent studies. Two population-based studies concluded that significant exposure-response (E-R) trends between cumulative diesel exhaust and lung cancer were unlikely to be entirely explained by bias or confounding. Those studies have quality data on life-style risk factors, but do not allow definitive conclusions because of inconsistent E-R trends, qualitative exposure estimates and exposure misclassification (insufficient latency based on job title), and selection bias from low participation rates. Non-definitive results are consistent with the larger body of population studies. An NCI/NIOSH cohort mortality and nested case-control study of non-metal miners have some surrogate-based quantitative diesel exposure estimates (including highest exposure measured as respirable elemental carbon (REC) in the workplace) and smoking histories. The authors concluded that diesel exhaust may cause lung cancer. Nonetheless, the results are non-definitive because the conclusions are based on E-R patterns where high exposures were deleted to achieve significant results, where *a posteriori* adjustments were made to augment results, and where inappropriate adjustments were made for the “negative confounding” effects of smoking even though current smoking was not associated with diesel exposure and therefore could not be a confounder. Three cohort studies of bus drivers and truck drivers are in effect air pollution studies without estimates of diesel exhaust exposure and so are not sufficient for assessing the lung cancer-diesel exhaust hypothesis. Results from all occupational cohort studies with quantitative estimates of exposure have limitations, including weak and inconsistent E-R associations that could be explained by bias, confounding or chance, exposure misclassification, and often inadequate latency. In sum, the weight of evidence is considered inadequate to confirm the diesel-lung cancer hypothesis.

## 1. Introduction

Since reviewing the epidemiology of lung cancer and diesel exhaust (Gamble 2010) seven additional diesel studies have been published (Birdsey et al., 2010; Merlo et al., 2010; Petersen et al., 2010; Olsson et al., 2011; Villeneuve et al., 2011; Attfield et al., 2012; Silverman et al., 2012). Two of these are large pooled population-based case-control studies. One looks at populations in Europe and Canada (Olsson et al., 2011), and has been the subject of previous comments and responses (Mohner 2012; Morfeld and Erren 2012; Olsson et al., 2012). Results from three of the countries included in the pooled analysis had been published earlier and reviewed previously (Bruske-Hohlfeld et al., 1999; Gustavsson et al., 2000; Richiardi et al., 2006). The second large population-based case-control study looks at populations in eight Canadian provinces (Villeneuve et al., 2011) and is similar in methodology to the previously reviewed Montreal cohort (Parent et al., 2007).

Two of the other recent studies involve the same group of underground (UG) non-metal miners that is the subject of the Diesel Exhaust in Miners Study (DEMS) (Attfield et al., 2012; Silverman et al., 2012). One is a cohort mortality study (Attfield et al., 2012) and the other a nested case-control study, with information on smoking, complete work histories and other potential confounders (Silverman et al., 2012). Surrogate-based quantitative estimates of respirable elemental carbon (REC) are used in exposure-response (E-R) analyses. Exposure estimates are based on recent sampling and historical samples of CO as well as on estimates of CO based on diesel engine horsepower and mine ventilation rates (Coble et al., 2010; Stewart et al., 2010; Vermeulen et al., 2010a,b; Borak et al., 2011; Stewart et al., 2012).

The remaining three studies (Birdsey et al., 2010; Merlo et al., 2010; Petersen et al., 2010) are cohort studies of bus drivers and truck drivers. Risk is evaluated based on employment in these occupations without estimates of diesel exhaust exposure and no E-R analyses.

An updated critical review of these studies is needed because of upcoming health hazard assessments by Authoritative Bodies. In June, 2012 a Working Group of the International Agency for Research on Cancer (IARC) will update their 1989 review of diesel engine exhaust (IARC 1989). In that review IARC concluded that the epidemiology data were “limited,” and classified whole DE as a “probable” human carcinogen.

The National Toxicology Program (NTP) is planning to update their 2000 review of diesel exhaust particulates (NTP 2000). In that review NTP concluded that DE particulate could be “reasonably anticipated to be a human carcinogen” based on increased lung cancer rates in workers exposed to DE, but noted there were no quantitative risk assessments for DE carcinogenicity.

This update of the previous review (Gamble 2010) is focused on studies with quantitative (or semi-quantitative or qualitative) estimates of exposure that were previously unavailable for the earlier IARC and NTP reviews (IARC 1989; NTP 2000). In their upcoming hazard assessments, these agencies will be considering carcinogenicity based on semi-quantitative E-R analysis for the first time. A reliable biological gradient in a study is important evidence for or against a causal association and determination of carcinogenicity. Authoritative Bodies and Regulatory Bodies in their review of epidemiological data need to focus on issues of exposure assessment, confounding and other hidden uncertainties, as well as chance when considering the reliability of E-R trends. In that regard, many important questions need to be addressed: are the observed trends accurate representations of the true associations? Do the study subjects have adequate latency to attribute increased lung cancer risk to occupational DE exposure? Is exposure misclassification of sufficient magnitude to produce spurious increases in lung cancer risk and changes in E-R patterns that can affect the interpretation of possible cancer etiology?

Adequate latency and potential misclassification of DE exposure were, and remain, of particular concern in retrospective studies of diesel-exposed workers (Gamble 2010). Heterogeneity of diesel engines in the workplace (including their rate of introduction) and the resultant frequent exposure misclassifications have occurred because either the heterogeneity issue was ignored and investigators simply assumed that diesel engines were present in the workplace and that all workers were exposed to DE, or because the time and rate for the introduction of diesel into the workplace was incorrect, unknown, or could not be determined for individual subjects.

The importance of latency was reconfirmed by an HEI (Bailar et al., 1999) review where it is stated, “The study design chosen needs to allow for an adequate latent period for developing the health outcome of interest after exposure to the risk factors studied. For some cancers the latent period may be 20 to 40 years…Latency period, timing of exposures, duration of exposures, and exposure-response measures are all interlinked, and all are essential to a complete assessment of risk.”

Although more than a century has passed since diesel engines were first introduced into the workplace, latency remains an issue in contemporaneous studies (Gamble 2010) as well as in one of the recently published studies. An extension of the latency issue relates to the changing composition and reduced magnitude of DE emissions. In our review, we are evaluating studies that attempt to assess DE exposure beginning in the 1920s and extending through the 1980s and occasionally into the 1990s. Diesel emissions have evolved dramatically over these 70+ years, and several recent papers provide increased clarification regarding the changes in the levels and composition of diesel emissions (Hesterberg et al., 2011; Hesterberg et al., 2012). Those papers provide detailed definitions of three generations of exhaust emissions: Traditional Diesel Exhaust (TDE) (pre-1989 engines), transitional diesel exhaust (1989–2006 engines), and New Technology Diesel Exhaust (NTDE) (2007 and later engines). Thus, the latency issue is compounded by the question of which generation of exhaust the workers may have been exposed to, and for how long.

Chronic diseases such as lung cancer require decades from initial exposure for the development of lung tumors. Because of the requirement of a long latency period, epidemiology can only address associations of TDE with lung cancer, not transitional diesel exhaust and certainly not NTDE. In their upcoming reviews of DE and lung cancer, IARC and NTP will need to recognize that the only epidemiology studies that are available for evaluating the potential cancer risk of diesel engine exhaust are studies of TDE.

This is the background for the previous review (Gamble 2010) and remains the same for this update. The purpose of this review is to update that critical review of the relevant epidemiology studies with newly published studies (Olsson et al., 2011; Villeneuve et al., 2011; Attfield et al., 2012; Silverman et al., 2012) that may be useful for testing the diesel-lung cancer hypothesis.

This review will first consider the population-based case-control studies (Olsson et al., 2009; Villeneuve et al., 2011) followed by the NCI/NIOSH studies of non-metal miners (Attfield et al., 2012; Silverman et al., 2012) and ending with cohorts without estimates of DE (Birdsey et al., 2010; Merlo et al., 2010; Petersen et al., 2010). But first we briefly summarize each study to assist the reader in following the detailed discussions regarding our conclusions.

### Population-based case-control studies

A major limitation of population-based case-control studies is the difficulty in defining exposure because of the wide range of occupations and negligible information on individual jobs or workplaces. Exposure is generally not based on specifics of individual workplaces in time, but rather is ranked based on generalities that often have limited relevance to study subjects.

### Pooled study of populations from Europe and Canada (Olsson et al., 2012)

Exposure assessment is an important concern in the pooled study of 11 case-control studies in Europe and Canada, which include over 13,000 cases and controls. This and other factors preclude a definitive conclusion regarding the association of lung cancer and DE in this study. Other factors include: the wide range of exposure history beginning in the 1920s, which increases the probability of exposure misclassification; less than 20-year latency periods since initial DE exposure for many subjects, such that lung cancer caused by DE exposures late in life is implausible in many cases; and inadequate adjustment for potentially confounding occupational exposures (e.g., silica, asbestos) and possibly other carcinogens.

Exposure misclassification appears to be high for work histories prior to the 1970s that are classified as diesel-exposed when the probability of actual diesel exposure for most jobs was low (i.e., for jobs prior to 1970, the probability of diesel exposure was less than 50%). Latency is too short to attribute any increased risk to DE exposure when the bulk of the exposures occurred after 1970, since there were relatively few diesels in the workplace before then, and since the exposure assessment did not take time and dieselization into account.

Selection bias from low participation rates also potentially produces spurious associations in the pooled studies. The best documented rate is the 40% participation rate among the better educated, healthier controls, which biases the OR away from the null in the German part of the pooled results. Nevertheless, the strength of association is still weak with ORs less than about 1.3 in high exposed categories. Overall, this study does not provide consistent evidence of an association between DE exposure and lung cancer. Although its results are compatible with the diesel-lung cancer hypothesis, the results could well be due to residual confounding. The authors conclude there is a small, consistent association between occupational diesel exposure and lung cancer after adjustment for potential confounders. We suggest the results are indefinite with regard to the diesel-lung cancer hypothesis.

### Population-based case-control study of Canadian men (Villeneuve et al., 2011)

A strength of the Canadian study is the expert-based exposure assessments made on a case-by-case basis taking into account the era of employment to reflect the shift from gasoline to diesel engine use. Exposure periods ranged from the year 1920–1997, so this effort was essential to ameliorate exposure misclassification. Fifty six percent of cases were considered “ever” exposed to diesels.

The authors (Villeneuve et al., 2011) concluded that there was a “dose-response relationship between cumulative occupational exposure to diesel engine emissions and lung cancer,” which was more pronounced for the squamous and large cell subtypes.

E-R trends are marginally significant for squamous and large cell carcinomas (or not significant if multiple testing is taken into account) and E-R associations are uncertain because of weakly positive but statistically non-significant E-R trends. Several limitations are suggestive that the results of this study do not support the diesel hypothesis:

(i) Excess risks occurred among “truck drivers, taxi drivers and railway conductors,” and the risks for squamous cell lung cancers were sometimes increased 3–4 times. But ORs were only about 1.4 times greater for those jobs and DE exposure ranged from 0 to 100% during the early 1980s. Assessing risk by job is an inherent problem with population-based case-control studies because it produces multiple testing of dozens of different jobs (and in this case several cell types as well). Thus some “statistically significant” results will occur by chance and it becomes problematic to determine which results do not constitute “false positives.”
(ii) The cell type results are a sub-type analysis that is inconsistent with other studies of diesel-exposed workers. The only significant E-R trends observed were for squamous and large cell carcinoma, only one of which was an *a priori* hypothesis.
(iii) E-R trends disappeared after adjustments for smoking, exposure to second-hand smoke, and occupational exposures to asbestos and silica.
(iv) As with the Olsson et al., pooled study, low participation rates may bias results, since the controls had higher incomes and more education than cases, while the cases were heavier smokers and included many fewer non-smokers than controls. These differences indicate that the controls were not representative of cases in terms of income, education and smoking and could have biased the results because of the reduced risk of lung cancer associated with higher income and education and reduced smoking. While smoking is adjusted for, adjustments for the potential positive confounding effects of income and education might further reduce the lung cancer risk.

Even so, this is a well-conducted study that attempts to adjust for potential occupational and non-occupational hazard (e.g., silica, asbestos, cigarette smoke). Accordingly, it is noteworthy that there are no apparent associations of diesel emissions with all cases of lung cancer after adjustments for these confounding exposures. ORs for squamous cell and large cell carcinomas are excessive at high DE exposures, but a biological mechanism is unclear and the lack of consistency with other diesel studies weakens any causal attribution.

### NCI/NIOSH Studies of non-metal miners exposed to diesel exhaust (Attfield et al., 2012; Silverman et al., 2012)

These studies include a cohort and a nested case-control study of about 200 lung cancer cases. This is an important cohort because DE is highest among UG miners; quantitative estimates of DE exposure are premised on a seemingly plausible surrogate for DE (respirable elemental carbon or REC); information on potential confounders is available from the nested case-control study; there is unlikely confounding from noncarcinogenic mining exposures; and there is adequate latency for occupational lung cancer to develop.

In the cohort study, lung cancer SMRs were 1.33 for surface workers and 1.21 for ever underground (UG) workers, even though the average REC exposure was eight times greater for UG workers. E-R trends among the particular sub-group of UG workers with >5-years tenure, a 15-year lag, and REC exposures restricted to <1280 µg/m^3^-years were the basis for the authors’ conclusion that these findings “provide further evidence that diesel exhaust increases risk” of lung cancer.

The evidence from the cohort study is considered inadequate for assessing associations of lung cancer and diesel exhaust for several reasons, not least of which is the nested case-control study has additional information on potential confounders such as smoking. The findings are considered inconclusive because the “significant” findings are mostly based on *a posteriori* analyses which include the elimination of the highest exposure group (>1280 µg/m^3^ years); exclusion of workers with <5-years tenure; because associations are weak, inconsistent and often statistically insignificant; and significant E-R trends are model dependent. The potential for exposure misclassification also is considered high.

The nested case-control study consisted of 198 cases and 562 controls from eight non-metal mines that were matched by mine, sex, race/ethnicity, and birth year. Information was collected on other potential confounders, including smoking and education as well as lifetime work histories for employment in other high risk jobs and potentially carcinogenic workplace exposures. Results claimed a “strong and consistent” E-R relationship between lung cancer and REC with about a three-fold increased risk in the highest exposure quartile. The authors concluded DE “may cause lung cancer in mine workers.”

Notwithstanding the authors’ assertions, the results from the case-control study are considered inadequate for assessing lung cancer risk for several reasons:

(i) Current smoking is not a confounder, and the reported lung cancer risk appears to result from incorrect adjustments for smoking that spuriously elevate E-R trends at higher exposures.
  - The contention of “negative confounding” from smoking is based on an assumption of lower smoking rates among high exposed UG workers. But the reported results are based on all study subjects where there is no association of smoking with DE exposure; therefore smoking is not a confounder. As a result, the observed E-R trends are largely based on an incorrect adjustment for a non-existent confounder.
  - Crude ORs indicate flat E-R trends with no excess lung cancer risk at any exposure level.
  - The “adjustment effect” is biologically implausible and displays characteristics of an unstable model.
  - The E-R results require independent re-analyses and confirmation before the results can be considered reliable.
(ii) The exposure assessment of REC is too uncertain for any reliable analyses of E-R trends.
  - Historical estimates of post-1976 REC exposures are based on linear extrapolation of CO → REC. The CO: REC relationship is not linear and the correlations are poor. Moreover, introduction of diesel oxidation catalysts in the year 1970–1980 converted CO → CO_2_ and further weakened any CO: REC relationship. In addition, nearly half the CO measurements were below the LOD.
  - Pre-1976 REC estimates are based entirely on extrapolations of HP → CO → REC, and the relationship of HP and CO is not general, but rather is specific to individual engines and loads, and records of historical engine usage are inadequate.
  - Independent replication of exposure assessment results has not been possible, making further investigation necessary.

## 2. Population-based case-control study: Olsson et al. (2011)

### 2.1 Description

This paper uses a pooled data set from eight population-based, eight hospital-based and one hospital- and population-based case-control studies with 13,304 cases and 16,282 controls. Data were collected during the period 1985–2005 and diesel exhaust (DE) exposures were from 1922 to 2005. The data from 11 of the 17 lung cancer case-control studies are part of the SYNERGY project, which had the primary objective to study the joint effects of exposure to occupational lung carcinogens (asbestos, PAHs, nickel, chromium, silica) and smoking in 13 countries. The SYNERGY project was not specifically directed at assessing exposures to diesel exhaust. Three experts assigned exposure scores of 0 = no exposure, 1 = low exposure, or 4 = high exposure for 202 (11%) low exposure jobs and 27 (1.5%) high exposure jobs. Cumulative exposure was ∑ (intensity score = 0, 1, or 4) × (duration = years) = unit-years.

A general population job-exposure-matrix (GPJEM or ‘DOM-JEM’) approach was used to estimate DE exposure. This method was developed for general population studies with exposure assessment designed to be more general than specific, and was conducted by three occupational exposure experts who rated all job codes by intensity. The method was selected after comparison with two other exposure estimation methods in a study conducted in seven European countries (Peters et al., 2011). One was a population-specific JEM (PSJEM) that used experts to assess exposures of intensities >1 among controls by country. This assessment was then re-applied to all study subjects. Another approach used expert assignment of intensity of exposure on a case-bycase basis. Results were based on assessments of silica, asbestos and DE exposures. Comparisons between methods were based on strength of associations and heterogeneity of risk estimates between countries premised on the assumptions of similar intensities and duration of exposure, and similar biological effects between countries.

Results between countries were significantly heterogeneous for all three methods. The prevalence of DE exposure was generally higher for DOM-JEM (22%) than the other two methods (16 and 19%) and there was excellent agreement between the experts for the DOM-JEM method. However, as Peters et al. point out, this evaluation provides little information on validity of the assessments, but poor agreement is suggestive of considerable misclassification. Risk estimates for DE were comparable (1.08, 1.05, and 1.05) for expert assessment, PS-JEM and DOM-JEM respectively. Case-by-case expert assessment has theoretical advantages such as more accurate exposure estimates, at least for single-center studies. Nevertheless, the DOM-JEM was selected for use in the multi-center study (Olsson et al) because there was said to be little, if any, advantage of case-by-case assessment and DOM-JEM was cheaper and quicker (Peters et al., 2011).

Two sets of odds ratios (OR) were estimated. OR1 = adjustments for age, sex, study (country), and ever employment in high risk job. OR2 = additional adjustments for pack-yrs. and time-since-quitting smoking. Only OR2 will be reported unless noted otherwise.

The demographics of the cases tended toward confounded results, with certain possible exceptions such as fewer former smokers and somewhat better participations rates. Sex and age distribution of cases and controls were similar. Potential confounding biases included participation rate, smoking, working in jobs with lung cancer risk, and potential misclassification of diesel exposure.

**Table:** 

	Cases	Controls
		
*N*	13,304	16,283
Average participations rates	82% (68–98)	67% (41–100)
% Non-smokers	6%	29%
% Former smokers	29%	39%
% Current smokers	65%	32%
% Exposed to diesel exhaust	42%	37%
% Employed in other high risk jobs	12%	8%

### 2.2 Results

**Figure 1:**
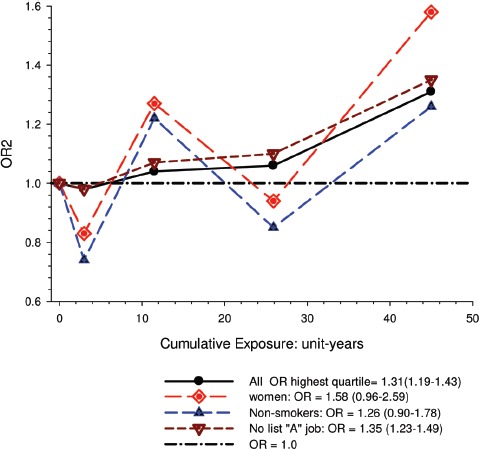
Exposure-response of lung cancer and cumulative DME exposure among all cases and controls, women, non-smokers, those without working in jobs with known lung cancer risk; ORs adjusted for age, sex, study, ever employment in list “A” jobs, packyears, time since quitting smoking (Olsson et al., 2011).

Overall there was a significant linear trend (*p* <0.001) with ORs by increasing quartile of cumulative exposure of 0.98 (95% CI 0.89, 1.08), 1.04 (95% CI 0.95, 1.14), 1.06 (95% CI 0.97, 1.16), and 1.31 (1.19, 1.43). E-R analyses showed significantly increased OR2 in the highest quartile exposure category for all subjects, men, women, never-smokers and those never employed in high risk jobs. There was no increased risk for the lower exposure quartiles (Figure 1).

**Figure 2:**
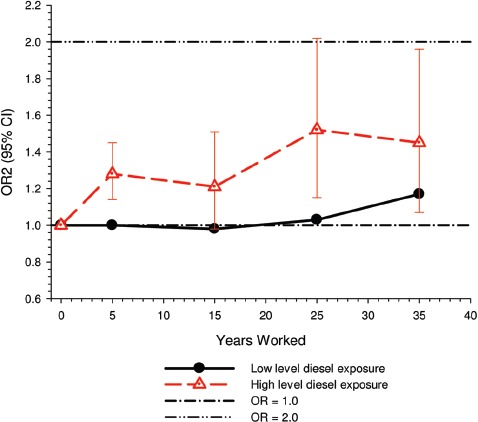
Lung cancer risk by years worked among workers only exposed to low levels and high levels of diesel motor exhaust exposure (Olsson et al., 2011).

E-R among workers with high levels of DE exposure showed excess risk associated with as little as 5-years exposure, while workers exposed only to low levels of DE exposure “showed elevated risk only after 30 years and more” (Figure 2).

The authors concluded that “Our results showed a small consistent association between occupational exposure to DE and lung cancer risk and significant exposure-response trends… This association is unlikely to be entirely explained by bias or confounding.”

### 2.3 Strengths

The studies key strength is its large sample size. Even in the highest exposure quartile there were a large number of cases. The study also benefitted from good quality data on smoking and occupational history, with job exposures assigned by experts.

### 2.4 Limitations

#### 2.4.1 Exposure era is unaccounted for, potentially producing biased and spuriously elevated risk estimates

The era and location when diesel exposure occurred is very important in assessing diesel exposure, as the introduction and proliferation of diesel engines in the workplace varies over time, workplace, and country. Current exposures do not reflect past exposures, and past exposures classified as “diesel exposures” may actually be more non-diesel than diesel, or have a low or negligible probability of diesel exposure unless the calendar time of past exposure is accounted for. The authors specifically note the limitation of the general population job-exposure-matrix (DOM-JEM) used in this study:

“The high prevalence of exposure is also a consequence of the nature of a JEM, namely to assign everybody in a given job code the same exposure, whereas individual assessments give the opportunity for attributing exposure to some people in a job but not others, and to take into account an increasing trend of diesel engines over time. This may contribute to multiple dimensions of exposure misclassification.” (Olsson et al., 2011)

There are several sources for exposure misclassification, and it is unlikely to be non-differential misclassification as suggested by the authors. At least three factors must be considered: (i) reductions in diesel emissions over time due to improvements in diesel engine technologies and fuels; (ii) the associated time lag associated with the introduction of new diesel engines into the workplace; and (iii) the era of diesel engine and fuel technology for the relevant exposures (factoring in 20-years latency) of the study population. If those factors are not carefully considered, a large number (and perhaps a majority) of workers are deemed “exposed” when in fact they were not actually exposed to DE.

(i) Diesel emissions (particulate matter in the US) began to be regulated in 1988, when PM emissions were reduced by 60% from pre-1988 emissions (Hesterberg et al., 2011). Dieselization began earlier in Europe than in the US. For example, since the late 1940s diesel trucks have comprised the majority of the Danish truck fleet (Hansen 1993). In Geneva, Switzerland diesel lorries first started in service in 1928, and became more widely used during the 1950s and 1960s. From 1950 to the mid-1980s, motor vehicles increased from about 12 vehicles per 100 population to 60 vehicles per 100 population. Most of those vehicles ran on petroleum and diesel lorries and declined from 11 to 5% over that time period (Guberan et al., 1992). In Sweden, since 1945 all buses have been diesels (Gustavsson et al., 1990). By the end of WW II, about 85% of buses in London were diesels, and all were diesels by 1950 (Rushton et al., 1983).
  An example of the uncertainty associated with the categorization of workers as exposed or not (i.e., high, medium, low) is provided in an early study by Hall and Wynder who categorized some occupations as “high exposure” when as few as 20% of workers in a job category were exposed to DE. “Moderate exposure” was 10–19% exposed and “low exposure was <10% exposed (Hall and Wynder 1984). It appears that Olsson et al. used a similar misclassification system, although using different cutpoints. Job categories with <33% diesel use were considered “non-exposed” (Peters et al., 2011). As a result, potentially about 1/3 of “non-exposed” workers could be exposed and about 1/3 of “exposed” workers could be “non-exposed” by this criterion. Diesel exposure based on job title is an estimate based on general probabilities that are unreliable with the possible exception of jobs where all engines are diesel for the era of concern.
(ii) Diesel engines generally comprised <50% of the engines used in many jobs through 1980, including motor transport, taxi drivers, truck drivers, mechanics, motor vehicles, industrial trucks, locomotive operators, and dockworkers (Parent et al., 2007).
(iii) DE exposure in this study potentially occurred from 1922 to 2005. After accounting for a 20-year latency, the latest job exposures of relevance for attributing lung cancer to diesel exposure ranges from 1972 (France) to 1985 (Italy, UK).

The probability of DE exposure ranges from 0 to 100%, depending on time and location. For many workers, DE exposure commonly occurred during the last few years of their working lifetimes because few diesels were present in the workplace before then. Parent et al. (Parent et al., 2007) estimated the percent of diesel exposure in different jobs for the years 1979–1985 in Montreal, Canada, which is the effective time period for the end of the relevant diesel exposure for this study. Those Canadian data indicate diesel exposure misclassification will be common for many jobs, and there may be complete misclassification for some jobs, when the era of dieselization is not taken into account. For example, Parent et al., were highly confident that exposure levels were low, despite the frequency of exposure being high for about one of every four locomotive operators. And for time periods going back to 1922, the likelihood of misclassification only increases (Table 1).

**Table 1:** Proportion of workers exposed to diesel emissions in selected occupations and usual exposure coding, Montreal, Canada, 1979–1985 (Parent et al., 2007).

		Diesel emissions		
		
		Usual exposure coding		
		
	% diesel-exposed	Confidence*	Concentration*	Frequency†
Motor transport workers	37	3	1	3
Bus drivers	91	3	2	1
Taxi drivers & chauffeurs	0		1 (1)	3 (3)
Truck drivers (heavy trucks)	39(54)			
Mechanics	28	3	2	2
Motor vehicle & repairers	29	3	2 or 3	3
Industrial trucks	0			
Salesmen	1	2	2	1
Commercial travelers	0			
Route drivers	0			
Railway Transport Workers	72	3	1	3
Locomotive operators	25	3	1	3
Conductors & brake workers	82	3	1	3
Excavators and pavers	56	2 or 3	2	2
Excavating, grading & related occupations	95	2	2	2
Miners and quarrymen	79	2	2	3
Firefighters	95	3	2	2
Dockworkers	27	3	2	2

* Concentration and confidence levels: 1 = low, 2 = medium, 3 = high.

† Frequency levels (% of normal workweek): 1 = <5%, 2 = 5–30%, 3 = >30.

The JEM method “did not take into account changes in the use of diesel engines over time.” Diesel engine use was low or negligible in many jobs as late as the 1980s, and the percentage of exposed workers declines going back in time. Work histories began in the 1920s in five countries, during the 1930s in 10 countries, and in the early 1940s in two countries. Exposure misclassification is nearly assured during those early periods when more than 50% of jobs were non-diesel, and misclassification remains high even up to 20-years before diagnosis when time changes are not taken into account, based on the Canadian data (Parent et al., 2007). The Canadian study is the only study we know of that has assessed the percentage of diesel-exposed workers, and Canada is thought to be similar to Europe.

Unless diesel exposure is individually confirmed, it appears probable that early exposures are a variable mixture of diesel and non-diesel emissions. Exposure misclassification appears less likely for later periods of the occupational history, but even if diesel exposure is correct, the latency will be too short to plausibly attribute lung cancer etiology to diesel exposure occurring within 20-years of diagnosis (Gamble 2010).

An assumption of non-differential misclassification may not be correct as misclassification will vary from job to job since the introduction of diesel engines was not a constant. Coding a job “diesel-exposed” when in actuality there are few or no diesel engines, produces an over-estimation of exposure. Based on the data provided and the estimated time-table for diesel use, it is likely the risk estimates are incorrect and largely inapplicable to diesel emissions. The estimates are also likely non-differential since misclassification increases as one goes back in time and diesel use in the workplace decreases. Exposure misclassification is differentially increased in occupations or jobs where the introduction of diesels occurred over a relatively long time and for workplaces containing <100% diesels. For example, among motor transport workers during 1979–1985, about 37% were exposed to diesels. Exposure misclassification would be greater among truck drivers (39% exposed) than heavy truck drivers (54% exposed) or bus drivers (91% exposed). Similarly, among railroad workers, misclassification would be greater among locomotive operators (with 25% exposed) compared to conductors and brake workers (with 82% exposed) (Table 1).

An analysis of risk among workers who began employment when more than 50% of engines in each job category were diesels would provide some reassurance regarding the validity of DE exposure estimates in this study. In that regard, the following is a series of discussion points concerning the Olsson et al. (2011) paper.

#### 2.4.2 Assumption of non-differential exposure misclassification does not necessarily mean attenuation of E-R

In the original paper and in their reply to Bunn and Hesterberg (2011), the authors argued that exposure misclassification was most likely to be non-differential between cases and controls, because it was done independent of case-control status, and led to attenuation of OR estimates “in most scenarios” (Olsson et al., 2011; Olsson et al., 2012). However, this might not be true for several reasons:

(i) Exposure assessment was based on the application of a job exposure matrix to the occupational histories reported by study subjects. Subjects’ recall of occupational histories can differ systematically between cases and control, as in the case of other environmental exposures (Rothman et al., 2008).
(ii) In the case of multiple exposure categories, non-differential misclassification may lead to an over-estimation of the risk parameters, especially when it occurs among non-contiguous exposure categories (Dosemeci et al., 1990; Birkett 1992; Wacholder 1995)
(iii) Jurek et al. (2005) conducted a simulation study that indicated bias towards the null cannot be assumed. Over-estimation also occurs and many factors, including true OR, exposure prevalence, unexposed risk, misclassification rates, and other factors that influence bias and random error, determine whether the observed OR is underestimated or over-estimated. As the true RR is decreased, the probability of over-estimating the measured RR is increased. This is the situation in this study where ORs are consistently less than 1.5.

#### 2.4.3 Uncertainties associated with qualitative dichotomous categorization of Jobs and selection of indices of intensity

The methods section of Olsson et al. (2011) indicates that scores of no exposure = 0, low = 1, or high = 4 exposure levels of DE were assigned to each ISCO job code. Categorical exposure categories such as these necessarily produce misclassification of intermediate intensities:

(i) Intermediate intensities normally classified as medium exposures but categorized as high in this dichotomous scheme over-estimate the true exposure, thereby spuriously under-estimating risk.
(ii) Intermediate intensities normally classified as medium exposures but categorized as low exposure jobs under-estimate the true exposure intensity, thereby spuriously over-estimating risk.

Morfeld and Erren (2012) questioned the rationale for using 1 and 4 as indicators of low and high exposure in the pooled analysis when intensity levels of 1 = low, 2 = medium and 3 = high were used in the background publication that was cited as support for the exposure assessment (Peters et al., 2011). Morfeld and Erren suggest “results may depend considerably on the chosen numeric interpretation of categories.”

Olsson et al. (2012) indicated that assigned relative scores of 1 and 4 seemed “reasonable” based on reported differences in exposures to elemental carbon (EC) – exposures of 7 µg/m^3^ for low exposed drivers versus 25 µg/m^3^ for high exposed mechanics, and ∼15 µg/m^3^ for low exposed surface miners versus ∼160 µg/m^3^ for UG workers (Pronk et al., 2009). The score is at best a “semiquantitative measure of DE exposure.” Presumably referring to the ranking score used, Olsson et al. (2012) suggest that “different weights for intensity would not have changed these overall findings.”

Table 2 summarizes the results from comparisons of different exposure models using different ranking weights for intensity (Peters et al., 2011). However these comparisons may not be a valid test because factors other than indices of intensity may not be the same. Information provided by Olsson et al. is inadequate to determine if the scoring method makes a substantial difference in the estimated risks. Morfeld and Erren (2012) suggest sensitivity analyses should have been performed to test the effect of changing intensity scores. The sensitivity analysis should be systematic as small changes in “data staging rules” can have profound effects on ORs, and the concern is that the small ORs (less than 2.0) may lack credibility.

**Table 2:** Comparison of ORs based on different DME exposure assessment and different relative scores for low and high exposure jobs (0 to 4) in the Peter et al. (2011) study of INCO countries.

Country	Expert assessment	Population-specific JEM	DOM-JEM-INCO	DOM-JEM pooled highest quartile
Intensity indices				
Non-exposed	0	0 (<33% exposed)	0	0
Low	1	1	1	1
Medium	2	2	2	4
High	3	3		
ORs: country (*n*)				
Czech Repl (285)	2.12 (1.41–3.19)	1.85 (1.24–2.78)	1.89 (1.31–2.73)	1.16(0.64–2.13)
Hungary (361)	0.81 (0.56–1.18)	1.07 (0.74–1.56)	0.88 (0.61–1.27)	1.27 (0.76–2.11)
Poland (539)	1.18 (0.56–1.37)	1.20 (0.89–1.64)	1.15 (0.87–1.53)	1.77 (1.08–2.90)
Romania (181)	0.62 (0.35–1.09)	0.68 (0.40–1.16)	1.08 (0.64–1.82)	0.99 (0.40–2.48)
Russia (506)	0.89 (0.66–1.22)	0.87 (0.65–1.16)	0.74 (0.56–0.98)	1.17 (0.74–1.86)
Slovakia (346)	1.27 (0.84–1.93)	1.07 (0.72–1.59)	1.43 (0.97–2.12)	1.60 (0.80–3.18)
UK (192)	1.00 (0.61–1.63)	0.71 (0.43–1.18)	0.65 (0.92–1.08)	0.93 (0.59–1.46)

We suggest actual EC data might be applied for ranking individual jobs instead of simply assuming that all high exposure jobs have 4 times more exposure to EC than all low exposure jobs. Even in the cited example, a rating of 4 for UG workers vs. above ground workers is inaccurate as UG workers have an 11-fold greater EC exposure than low exposed surface workers (Olsson et al., 2011).

A more accurate semi-quantitative measure of DE exposure would be to use scores based on sample data that reflect the reported differences in low, intermediate and high exposure jobs. For example, EC is “highly variable” in high exposure UG jobs so a score of 4 does not accurately represent UG jobs. To further emphasize the problem of variability, Pronk et al. (2009) reported EC exposures ranging from 27 to 658 µg/m^3^ in high exposed jobs, less than 50 µg/m^3^ in intermediate exposed jobs, and less than 25 µg/m^3^ in the lowest exposure jobs. If this wide range of difference between low and high exposed jobs exists in this study, a single weight for all high exposed jobs provides an inaccurate estimate of exposure.

In the UK, Groves and Cain (2000) sampled DE in 7 different work groups. They found that the 95th percentile values for EC in high exposed vs. low exposed groups were 17:1 for the 90th percentile and 9.5:1 for average EC exposures in the seven job groups. Given these results, a dichotomous approach of low and high exposures appears unacceptably variable for any accurate representation of actual DE exposure.

It is interesting to note that Villeneuve et al. (2011) had initially intended to use a JEM-like exposure assessment of DE exposure based on already assigned job titles and industry codes. However, when they attempted to verify the job codes, the accuracy was so low that they switched to an expert-based exposure assessment approach, which was considered the best available method for population-based case-control studies (Bouyer and Hemon 1993).

If exposure misclassification is high in contemporary jobs, the problem is amplified for exposures occurring more than 20 years ago when diesel exposures in the workplace tended to be markedly reduced and varied.

Olsson et al. (2011) note that the original studies estimated diesel exposure “using expert case-by-case assessment” by local experts and took into account the increasing use of diesels over time, which was considered a strength of the method. Nonetheless, in selecting the DOMJEM methodology, the diesel time variable was not adjusted for, since the same exposure was assigned to everybody in the same job. A consequence of assigning “everybody in the same job the same exposure” produced a high prevalence of DE exposure. In addition, not taking into account an increasing trend of diesel engines over time may contribute to multiple dimensions of exposure misclassification.” But because these factors are “not related to disease status it was claimed that they most often lead to an attenuation of the OR estimates (Olsson et al., 2011).

Jurek et al. point out that non-differentiality of exposure misclassification is not an adequate justification for suggesting that estimated risks are under-estimates, since many other factors must be considered (e.g., independence of errors, confounding, selection bias, mismeasurement of covariates) and quantitative methods such as sensitivity analysis, uncertainty analysis and bias modeling must be employed to account for systematic errors (Jurek et al., 2005).

#### 2.4.4 Latency was not taken into account

If a work-related lung cancer requires a latency of approximately 20-years, the diesel exposure period of interest in this study was during or before 1965–1985, or 20 years or more before the beginning and end of the cancer data collection period of 1985–2005.

Diesel exposures began in 1922 (Italy) and 1945 (The Netherlands). In Germany, the probability of DE exposure during 1988–1994 was less than 1% for farmers and more than 90% for drivers. For high exposed railway workers it was less than 25% (Bruske-Hohlfeld et al., 1999). But the relevant exposure periods still occurred well before 1988.

In Italy, the estimated probability of DE exposure for locomotive drivers was less than 33% in 1990. For forklift drivers, the probability of DE exposure was estimated between 33 and 66% in the 1990s and more than 66% during 1960–1980 (Richiardi et al., 2006). In Sweden, response data collection was in the period 1985–1990, so the relevant exposure period is before 1965–1970 (Gustavsson et al., 2000). Diesel-powered trucks were introduced in the 1950s and were dominant in the 1960s (Boffetta and al 2001). Nevertheless, most of the truck driver cases were retired, so the bulk of their work history was before the introduction of diesel engines (Gustavsson et al., 2000).

These three studies comprise about two-thirds of the participants in the pooled analysis (Olsson et al., 2011). Since time period was not considered in the exposure assessment, the occurrence of exposure misclassification and the too short latency period will be common throughout the study results as evidenced by the differing rates of dieselization in Germany, Italy and Sweden.

#### 2.4.5 Potential inadequate adjustment for confounders

**Figure 3:**
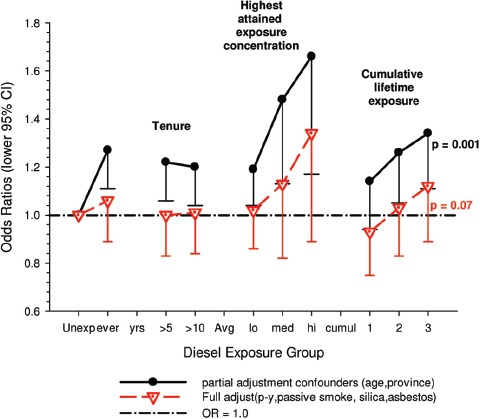
Adjusted odds ratios of lung cancer in relation to occupational exposure to diesel engine emissions with 5-year latency and men ≥40 years; partial adjustment for confounders is age and province; full adjustments for confounders is age, province, pack-years, second-hand smoke, silica and asbestos (Villeneuve et al., 2011).
  Data from the INCO country component of the pooled study (Peters et al., 2011) indicate asbestos and silica are potentially important occupational confounders. Those countries contribute 23% of the cases to the total of more than 13,000 cases. The DOM-JEM protocol was used for exposure assessment in the pooled analyses, and adjustments of lung cancer ORs were for center, age, smoking status, gender, and List A jobs similar to the other pooled analysis. The only apparent difference is the rating of high exposure jobs. Table 3 summarizes the prevalence of exposure and risk of lung cancer associated with silica, asbestos and DE in those countries (Peters et al., 2011).

There are also a series of potential confounders in this paper:

(i) *Occupational confounders* : OR1 and OR2 were adjusted for (Y/N) ever-employment in “List A” jobs. List A jobs represent a list of occupations and industries identified as presenting an excess risk of lung cancer (Ahrens and Merletti 1998; Mirabelli et al., 2001). There are several other important questions relating to the adjustments. How are adjustments made when exposures to list A chemicals differ; for example when exposures were to asbestos alone; or to asbestos + silica; or to asbestos + silica + non-diesel PAHs? Is adjustment the same even though the risk is presumably different in each instance? How are different risks associated with each substance accounted for?
  A stated object of the pooled study was to study the effects of exposure to occupational lung carcinogens including asbestos, PAHs, nickel, chromium and silica. It would seem more appropriate to have made adjustments for individual carcinogens rather than consider them all as a group. An individual approach would provide an improved adjustment with a greater reduction of residual confounding. Since the exposure data are available for these particular exposures, and in a number of cases it appears adequate to do these adjustments, it is unclear why this was not done. Or more to the point, it raises the question whether any adjustments were omitted that potentially change the effect of DE exposure. The next paragraph suggests that the answer to this question is “yes.”
  Villeneuve et al. (Villeneuve et al., 2011) suggest that no adjustments were made for potential confounding from silica and asbestos in the pooled analysis (Olsson et al., 2011), which might explain why ORs in the European and Canadian pooled study were higher. In the Canadian population-based case-control study (Villeneuve et al., 2011), adjustment for workplace exposures to silica and asbestos (in addition to pack-years and secondhand smoke) reduced ORs by 20–30%. The consistent effect was to change statistically significant results to non-significant results (Figure 3). For example, overall OR for “ever” exposure to diesel exhaust was 1.27 (1.11–1.44), and was reduced to a non-significant 1.06 (0.89–1.25) when fully adjusted. Similar adjustments in the pooled case-control study could have plausibly reduced overall OR and E-R trends to non-significance.

**Table 3:** Percentage of job periods exposed and risk estimates for lung cancer between three methods of exposure assessment in the INCO studies in Czech Republic, Hungary, Poland, Romania, Russia, Slovakia, UK from Peters et al. (2011).

	Expert assessment Peters et al. (2011)	Population-specific Peters et al. (2011)	GEM DOM-JEM (Peters et al. 2011) (used in pooled analysis)	DOM-JEM (Olsson et al. 2011)
Indices for rating intensity	0 = none; 1 = low; 2 = medium; 3 = high	0 = <33% exposed; exposed = >33% exposed to intensity ≥1	0 = non-exposed; 1 = low exposure; 2 = high exposure	0 = non-exposed, 1 = low; 4 = high
Asbestos				
% exposed (range)	7% (4–21%)	8% (3–20%)	26% (16–35%)	
OR (95% CI) (range)	1.12 (0.93–1.37) (0.77–2.23)	1.04 (0.86–1.26) (0.45–1.49)	1.19 (1.05–1.36) (0.96–1.5)	
Silica				
% exposed (range)	26% (10–54%)	31% (8–69%)	11% (4–26%)	
OR (95% CI) (range)	1.26 (1.10–1.44) (1.00–1.76)	1.24 (1.08–1.43) (0.89–2.11)	1.26 (1.08–1.47) (1.10–1.83)	
DME				
% exposed (range)	16% (9–27%)	19% (12–28%)	22% (12–33%)	
OR (95% CI) (range)	1.08 (0.94–1.25) (0.62–2.12)	1.05 (0.91–1.21) (0.71–1.85)	1.05 (0.92–1.20) (0.65–1.89)
  These data indicate the percentage of the study population exposed to silica (range of averages 11–31%) and asbestos (7–26%) was comparable to the percentage exposed to DE (16–22%), and the risk estimates for silica (OR = 1.24–1.26) and asbestos (OR = 1.04–1.19) are greater than for DME (OR = 1.05–1.08) (see Table 3). In this situation residual confounding is of particular concern because “noise” (from confounders) is greater than the signal (DE exposure). As a result part of the “noise” or effect from confounders may be counted as “signal” and thereby produce spurious risk estimates from unadjusted confounders. For example, confounding effects of smoking are always of concern because the risk of lung cancer from smoking is so much greater than any other lung cancer risk factor (i.e., a 20-fold increased for smoker vs. less than a two-fold for DE) (Table 3). These facts suggest that to avoid positive confounding and biased risk estimates in the pooled analysis, there should be individual-based adjustments and country-based adjustments for silica and asbestos. Thus, residual confounding from silica and asbestos seems likely.
  On the other hand, in Germany (Bruske-Hohlfeld et al., 1999) and Sweden (Gustavsson et al., 2000), adjustment for asbestos exposure tended to reduce ORs, but only to a small extent and did not appear to be a significant occupational confounder. But in Finland, adjustments for asbestos and quartz removed the observed effect of DE on lung cancer risk (Guo et al., 2004), so adjustments for those risk factors were needed to produce relatively unbiased risk estimates.
(ii) *Residual confounding by smoking* : adjustment for tobacco smoking had an important impact on the estimate of the association between DE exposure and lung cancer risk. In the main analysis (Table 3 in (Olsson et al., 2011) the adjustment reduced the OR in the highest quartile of exposure from 1.42 (1.31–1.54) to 1.31 (1.19–1.43), that is the degree of confounding from smoking is 1.08, or smoking accounted for about 8% of the excess risk. However, misclassification of tobacco smoking is likely to occur in retrospective case-control studies, leading to an under-estimate of the confounding effect and to incomplete adjustment (Savitz and Barón 1989). The lack of an effect among never-smokers (see below) further supports the hypothesis of an important role of confounding by tobacco smoking.
(iii) *Lack of association in never-smokers* : The results of the analysis restricted to 801 cases and 4,773 controls classified as never-smokers do not support the hypothesis of an association between DE exposure and lung cancer risk. Compared to unexposed workers, the OR1 in the four quartiles of cumulative exposure show no association with DE exposure and the *p* value of the test for linear trend was 0.28 (Table 3 in (Olsson et al., 2011)) (See Table at end of paragraph). These results are entirely consistent with randomness, and all CIs include 1.0.

**Table:** 

	Exposure Quartiles				
	
	Unexposed	1	2	3	4
OR (95% CI)	1.0	0.74	1.22	0.85	1.26
		(0.52–1.05)	(0.90–1.65)	(0.57–1.26)	(0.90–1.79)
  The authors justify this anomaly by citing the low statistical power of this analysis. This does not seem to be correct. Although it is not possible from the data reported in the publication to properly estimate the statistical power of the analysis among never-smokers after adjustment for covariates, it is possible to provide an estimate based on a crude analysis of DE exposed versus unexposed individuals. Although no results for ever versus never exposure are reported in the publication, a weighted average of the results reported in their Table 3 for the four quartile of cumulative exposure yields an OR of 1.20 (95% CI 1.15, 1.25). Given the number of never-smokers reported (187 exposed and 614 unexposed cases; 1287 exposed and 3486 unexposed controls), the analysis restricted to never-smokers would have had a statistical power of 80% to detect an OR of 1.23, which is close to the actual value of 1.20. It is worth noticing that the crude OR of the analysis of ever- versus never-exposed among never-smokers results in an OR of 0.82, with 95% CI 0.69, 0.98. In other words, there appears to be a statistically significant decrease in lung cancer risk among never-smokers exposed to DE.
(iv) *Confounding and education* : Mohner (Mohner 2012) pointed out that preliminary analysis showed adjustment for education status “halved the estimate of excess relative risk,” which suggests confounding in the final analysis because of the lack of adjustment for education.

As noticed by Möhner (Möhner 2012), the initial analyses of the pooled dataset included adjustment for education (Straif et al., 2010). Adjustment for education reduced the OR in the highest quartile of cumulative DE exposure from 1.27 (95% CI 1.14, 1.41) to 1.14 (95% CI 1.03, 1.26), a confounding effect if 1.11. Möhner (2012) provides evidence that in the German studies selection bias by education might have occurred. This evidence shows the need to adjust for education, even if education is not a good indicator of socioeconomic status as argued by authors of the paper in their reply (Olsson et al., 2012). But, in fact, education is associated with the likelihood of DE exposure, since low-skilled jobs are more likely to entail DE exposure. An under-representation of controls with low education would therefore result in an overestimate of the association between lung cancer and DE exposure. Based on the positive confounding from education in the preliminary results, the lack of adjustment in the final results suggests that residual confounding from SES is probable.

There is a strong correlation between educational attainment and exposure to most hazardous occupational substances; that is, subjects with less education have more hazardous exposures than subjects with more education. In the original analysis of the two German studies (Mohner et al., 1998), it was determined that controls without formal education training had 6.7 times more DE exposure than controls with a university degree, while the controls who had finished vocational training had only 3.5 times more DE exposure. Further, compared to the general population, the response rates were 1.8 times greater than expected for those with a university degree compared to 0.96 and 0.6 times expected for those with vocational training and without formal training, respectively (from (Mohner et al., 1998), summarized in Table 4).

**Table 4:** Associations of educational attainment with exposure to DME (Mohner et al., 1998).

Educational attainment	% exposure to DME	% of controls	Expected % controls (based distribution in general population)
No formal vocational training	24.7	10.0	16.9
Finished vocational training	13.0	68.6	71.5
University degree	3.7	21.4	11.6

Mohner (1998) also noted: “Therefore, if manual workers are underrepresented in the sample of controls, it follows that the exposure prevalence [to DE and list A chemicals] in the control group underestimates the true exposure prevalence in the reference population. Consequently, the *risk associated with a certain exposure* [DE] *is overestimated*.” [*Italics* added.]

The authors (Olsson et al., 2012) indicated that they were not certain “what attained education level reflects and if it is a real causal factor associated with lung cancer, after adjustment for other life-style factors such as smoking and occupational exposures to lung carcinogens, or that it is a correlate to DE exposure.” They commented that education was included in some models, but reduced ORs only slightly and had no effect on E-R patterns or significance. And they claimed Mohner (1998) himself reached the same conclusions regarding the lack of effect of adjustment on SES and response, citing the same study Mohner cited in his letter (Mohner et al., 1998).

There are several significant disagreements concerning the issue of confounding in this paper that we will try to sort out.

(i) The authors do not dispute the findings from the preliminary analysis where education reduced the reported results to non-significance. However, the authors claim that adjustments for education showed only a “moderate” effect, producing “slightly lower” ORs but similar patterns. A more informative response would be to show the adjusted and unadjusted ORs. Associations in this study are weak enough that “moderate” residual confounding may be enough to tip the evidence toward a conclusion of biased ORs and a conclusion of no causal association. There are enough questions about whether smoking and occupation exposures adequately remove confounding effects associated with education (or SES as suggested by the authors), and that conclusions measuring limited aspects of SES (including income, wealth, education, occupation, socioeconomic characteristics) may need to be reassessed (Braveman et al., 2005).
(ii) Inclusion of education in the model alone is not an adequate test for Mohner’s argument regarding natural selection (Möhner 2012). That is, lower educated controls have lower participation rates, making the referent group biased with fewer smokers and less occupational exposures to carcinogens, in addition to a higher average level of education. Distribution of controls compared to census data was suggested as a method for determining whether lower educated subjects are under-represented in the referent group.

Olsson et al. (2011) responded that the exclusion of Germany reduced the lung cancer OR to 1.22 (1.10–1.35) while the significant E-R trend (*p* < 0.01) remained. Presumably this is a reduction from the overall highest quartile OR, which in all subjects is 1.31 (1.19–1.43) in Table 3, and 1.26 (1.14–1.40) in their Figure 1. The major natural selection effect was from AUT-Germany, which had a 41% participation rate among controls. Participation rates were also low for Hda-Germany (68%), Canada (69%) and Italy (63%). Those countries contributed 22% of the controls after exclusion of AUT-Germany.

As noted, the authors stated they were not certain what education level reflects, or if it is a “real causal factor associated with lung cancer,” or if it is a correlate of DE exposure. Notwithstanding their uncertainty, what education level reflects is a correlation with important risk factors that cannot be directly adjusted for because they are unknown or unmeasured, and therefore cannot be adjusted for directly. Thus, educational attainment should be adjusted for in this study, particularly when the referent group is biased toward over-representation of those with higher education levels which in turn produces biased over-estimates of risk. In a Finnish study (Guo et al., 2004) the participation rate was essentially 100%, but removal of confounding by education, quartz, asbestos and smoking was still needed to adjust for residual confounding and to remove the upward bias in unadjusted ORs.

#### 2.4.6 Effect of study quality

In their Figure 1, Olsson et al. (2011) report study-specific ORs for the highest quartile of DE exposure. In sensitivity analyses, they classified the studies as population-based and hospital-based, and conducted separate analyses for the two groups: the resultant ORs were 1.30 (95% CI 1.17, 1.44) and 1.31 (95% CI 1.09, 1.59), respectively. Hospital-based case-control studies are more prone to bias than population-based case-control studies, and the lack of heterogeneity in the results of the two groups of studies can indicate robustness of overall results. However, this comparison ignores the fact that several population-based studies had low response rate, in particular among the controls. As noted, in the AUT study from Germany, the response rate among controls was as low as 41%. If one considers the studies with the highest quality (population-based studies with response rates of 80% or more in both cases and controls), the meta-analysis of results presented in their Figure 1 results in a significantly reduced OR of 1.14 (95% CI 0.95, 1.37). Along the same lines, Möhner (2012) showed a negative relationship between response rate among controls and study-specific ORs. Morfeld and Erren (2012) raised a similar criticism, but concentrated their argument on the inclusion of the AUT study: exclusion of that study reduced the overall OR for the highest quartile of DE exposure from 1.31 to 1.22 (i.e., that study alone contributed 29% of the excess risk found in the pooled analysis).

#### 2.4.7 Comparison with the study of US railroad workers

In their response to the criticisms by Bunn and Hesterberg (2011), Olsson and colleagues argue that their pooled analysis is more informative than the study of US railroad workers (Garshick et al., 2004), since their study includes more than 5600 lung cancer cases exposed to DE, as compared to less than 3400 cases in the US railroad study.

Olsson and coworkers, however, neglect two basic facts. First, the most important results in their study are based on workers in the highest quartile of cumulative exposure, which includes less than half the number of cases of the study of US railroad workers. Second, they note that their study would have been less prone to the healthy worker effect because it included the whole occupational history of study subjects. This would be a valid explanation only if railroad workers were exposed to lung carcinogens in jobs outside the railroad industry, but there is no evidence for that. Furthermore, there is weak evidence of a healthy workers effect for lung cancer. An alternative explanation is that no association exists between DE exposure and lung cancer risk, as found in the study of US railroad workers, and that the association found by Olsson and coworkers is the results of bias or confounding.

### 2.5 Summary

The overall OR for DE exposure in the pooled analysis of case-control studies is comparable to that found in previous studies and meta-analyses of DE exposed workers. This is not surprising, since similar biases are likely to have occurred in this analysis and in most previous studies. Although the overall results of the pooled analysis are suggestive of an association between high-level DE exposure and lung cancer risk, the results are not robust with respect to potential biases (e.g., low response rate in controls, possibly correlated with higher probability of exposure) and residual confounding (e.g., lack of association in never-smokers).

Exposure misclassification appears to be high for many participants’ lifetime work history. Probable misclassification occurs for pre-1970 jobs classified as diesel-exposed since the actual probability of diesel exposure for most jobs was low during that time period (i.e., less than a 50% probability of diesel exposure). Accordingly, diesel exposure is likely over-estimated and may be incorrectly represented as diesel exposure.

In addition, latency is too short to attribute increased risk to DE exposure when the bulk of the exposure occurred after the early 1970s. Before that time DE exposure was likely to be misclassified because there were relatively few diesel engines in the workplace and exposure assessments did not take time and dieselization rates into account.

Moreover, the strength of the overall association is weak and different ORs are reported for the highest exposure quartile, namely 1.31 (1.19–1.43) for all subjects in their Table 3, 1.26 (1.14–1.40) for all subjects in their Figure 1, and 1.22 (1.10–1.35) excluding AUT-Germany (because low participation rates).

Selection bias potentially produces spurious associations that should be adjusted to test the authors’ conclusions. Given the potential for substantive exposure misclassification and weak associations, this study is considered inadequate to attribute causality without analysis to adjust or correct for these biases.

Overall, this study does not provide consistent evidence of an association between DE exposure and lung cancer. Although its results are compatible with the diesel-lung cancer hypothesis, the results could also be due to residual confounding from occupational exposures (e.g., silica, asbestos), and low participation rates among controls, which produces residual confounding related to education or SES. The inability to differentiate between these and other factors suggests that the results are indefinite with regard to the diesel-lung cancer hypothesis.

## 3. Population-based case-control study: Villeneuve et al. (2011)

### 3.1 Description

This paper reports on a population-based case-control study of 1681 lung cancer cases and 2053 population controls from eight Canadian provinces, similar to an earlier Canadian population-based case-control study (Parent et al., 2007). Information from self-reported questionnaires included smoking and exposure to second-hand smoke, physical and demographic information, and complete work histories including potential exposures to silica, asbestos, gasoline and diesel emissions. Two industrial hygienists blindly coded occupations and job titles for gasoline and diesel emissions. For gasoline emissions, jobs were ranked for low (e.g., farmers), medium (e.g., taxi drivers, chauffeurs) and high (e.g., motor vehicle mechanics) concentrations. For diesel emissions typical jobs at low exposures included railroad conductors and brake workers; at medium concentrations jobs such as truck, taxi, and bus drivers in urban areas; and high exposure jobs such as garage diesel mechanics and UG miner workers. The frequency of exposures were coded as low frequency (less than 5% of work time), medium frequency (6–30% of work time), and high frequency (more than 30% of time). Reliability is based on the confidence that DE exposure was actually present in the job, and ranged from low (possible exposure), medium (probable exposure) and high (certain exposure).

Cases were from registries with histological confirmation and restricted to men over 40-years in age. Controls were identified from provincial health insurance plans and frequency-matched on age and sex. The most common cell types in this study were squamous cell carcinoma (36%, *n* = 602), adenocarcinoma (28%, *n* = 478) and small (16%, *n* = 267) and large cell carcinomas (10%, *n* = 166). Separate E-R trends were analyzed for each cell type.

Controls on average had a higher socio-demographic status and higher levels of education than cases. There were strong associations of lung cancer with passive and active smoking (pack-years, cigarettes/day, years smoked). For example, more than 60 packyear smokers had a 40-fold increased risk of lung cancer. Ever-exposure to silica and asbestos were associated with ORs of 1.19 (1.04–1.35) and 1.24 (1.09–1.43), respectively.

### 3.2 Results

**Figure 4:**
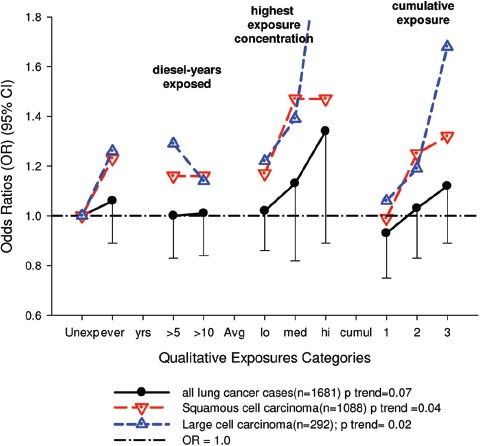
Adjusted odds ratios of lung cancer in relation to occupational exposures to diesel emissions, men 40+ years Canadian hospital-based case-control study (OR adjusted for age, province, pack-years, second-hand smoke, silica (Y/N), asbestos(Y/N)(*p* values refer to cumulative exposure) (Villeneuve et al., 2011).

**Figure 5:**
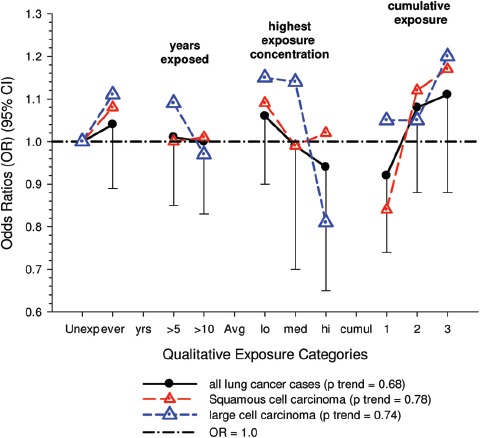
Adjusted odds ratios of lung cancer in relation to occupational exposures to gasoline emissions, men 40+ from Canadian hospital-based case-control study; ORs adjusted for age, province, pack-years, second-hand smoke, silica (Y/N) and asbestos (Y/N) (*p* values refer to cumulative exposure) (Villeneuve et al., 2011).

There were no significant associations between “ever exposed” to diesel emissions and lung cancer, and trends were “consistent” with an E-R pattern for highest attained exposure and cumulative exposure. Stratification by cell type suggested positive cumulative E-R associations for squamous cell and large cell types; *p* values for trend were 0.04 and 0.02 respectively. A reviewer commented that multiple testing by cell types changes the critical *p* value to 0.01. The only other significant association was for large cell carcinoma with an OR = 1.68 (1.03–2.74) at the highest tertile cumulative exposure category (Figure 4). There were no apparent associations between lung cancer and gasoline emissions (Figure 5). Most jobs involving exposure to diesel emissions had ORs greater than 1, and the associations were stronger for squamous cell lung cancers than for all lung cancers.

### 3.3 Strengths

Adjustments were made for potential confounders (active and second-hand smoke, silica, asbestos) that are often not made in other studies. Those confounders were shown to spuriously elevate ORs to statistical significance, which became statistically non-significant when adjusted for in the analyses (Figure 3).

Expert-based exposure assessment as used in this study is among the best methods for population-based case-control studies. The exposure assessments were made on a case-by- case basis and took into account the era of employment to account for the shift from gasoline to diesel engine use. This methodology was previously applied in the Montreal case-control study (Parent et al., 2007).

Attempts were made to account for the era of employment by consulting with local experts and industry associations with regard to the probable mix of diesel and gasoline engines in the workplaces. The exposure periods of participants ranged from the 1920 to 1997, so this effort to account for the particular era of dieselization at issue was essential to ameliorate exposure misclassification. 56% of cases were considered “ever” exposed to diesels. Essentially, all of the relevant diesel exposures were from Traditional Diesel Exhaust (TDE) before emission-control regulations took effect and began reducing levels of particulate emissions in diesel exhaust.

### 3.4 Limitations

#### 3.4.1 Gasoline engine emissions

There are clearly no associations of lung cancer with gasoline emissions in this study. These results are similar to the earlier population-based study in Montreal where ORs were consistently less than 1.0 for all levels of gasoline exposure (Parent et al., 2007). The authors concluded gasoline engine emissions were not related to lung cancer, but that risks may have been under-estimated because of exposure misclassification and nonoccupational exposure to gasoline. Limitations will be discussed in the section on diesel emissions.

#### 3.4.2 Diesel engine emissions

The authors (Villeneuve et al., 2011) concluded there was a “dose-response relationship between cumulative occupational exposure to diesel engine emissions and lung cancer. This association was more pronounced for the squamous and large cell subtypes.”

The E-R trends appear marginally significant for squamous and large cell carcinomas (or not significant if multiple testing is taken into account), but the association is uncertain because the E-R trends are only weakly positive and statistically non-significant (Figure 4). Associations with DE were based on several factors, but those factors do not add substantial weight to an interpretation of a causal association. The following points are suggestive that the results of this study do not support the diesel hypothesis:

(i) Excess risks occurred among “truck drivers, taxi drivers and railway conductors,” and the risks for squamous cell lung cancers were sometimes increased 3–4 times. This finding was said to be consistent with other studies, and “highlighted the need” to reduce exposures in these workplaces. The authors noted the strongest associations were for taxi drivers at 4.02 (2.03–7.97), excavators, graders etc. at 3.56 (1.86–6.83) and truck drivers at 2.83(2.0–4.0).
  In general, squamous cell carcinoma has a stronger association with tobacco smoking than all other cells. However, small cell carcinoma also has a strong association with smoking.
  For these jobs mentioned, the ORs for squamous cell cancers were all 1.4 times greater than for all cancers (see their Table 8). And these jobs have variable amounts of diesel exposure despite the similarity in ORs. According to Parent et al. (2007), diesel exposure without gasoline exposure is common for conductors. Truck drivers had 39–54% exposure to diesel emissions versus 100% exposure to gasoline emissions; taxi drivers had 0% exposure to diesels and 100% to gasoline emissions; excavating and grading had 100% exposure to gasoline and 95% to diesels during the period 1979–1985 in Montreal. Further, Parent et al. rated conductors and truck drivers with low exposure concentrations to diesel emissions (Parent et al., 2007), while in this study truckers, taxi and bus drivers were considered to have medium diesel exposure. Pronk et al. (2009) also found relatively low EC levels (<25 µg/m^3^) for drivers of on-road vehicles.

**Table 8:** Association of smoking prevalence by cumulative REC exposure among controls. Similar results obtained for cases and total cases + controls (calculated from Table 6 (Silverman et al., 2012).

	0–8 µg/m^3^ years	8–304 µg/m^3^ years	≥304 µg/m^3^ years
Non-smokers	10.5%	13.2%	8.0%
<1 pack/day	7.3	8.7	6.9
1–2 packs/day	13.9	15.3	11.2
≥2 packs/day	3.9	3.9	5.0
Unknown	4.5	4.1	2.1
  UG miners are among the highest diesel-exposed jobs (Pronk et al., 2009) and are considered highly exposed in this study. However their assessed diesel exposure is inconsistent with higher lung cancer risk associated with lower exposure jobs. The OR for miners and quarry-men is 2.12 (1.49–3.02) for all lung cancers, while the risk of squamous cell lung cancer is only 1.07 times greater, OR = 2.26 (1.37–3.72) (Villeneuve et al., 2011).
  This kind of data exemplifies a problem inherent to population-based case-control studies based on job categories. Multiple testing of dozens of different jobs (and in this case several cell types as well) may produce “statistically significant” results by chance, and it becomes problematic to determine “false positives.”
(ii) The cell type finding is based on a sub-type analysis that does not carry much weight and is inconsistent with other studies of diesel-exposed workers. The only significant E-R trends observed were for squamous and large cell carcinomas.
  It is not clear why these associations increase the weight of evidence implicating DE as a plausible etiological agent. Villeneuve et al. note that smoking is a strong risk factor for squamous and small cell carcinomas, and radiation increases risk of small cell carcinoma more than other cell types. They indicated squamous cell carcinoma risk estimates “were sometimes three to four times higher than the reference group of office workers.” Their source for this statement is not cited, but other diesel studies with analysis by cell types do not show the patterns displayed in this study (Figure 6). An *a priori* hypothesis was that stronger associations would be observed with squamous cell carcinoma and differential associations, if they existed, might have contributed to “discrepancies” in the results from previous studies (Villeneuve et al., 2011). The *a priori* hypothesis appears to be true, but there is no apparent consistent pattern in the limited amount of diesel literature on this issue (Figure 7).

**Figure 6:**
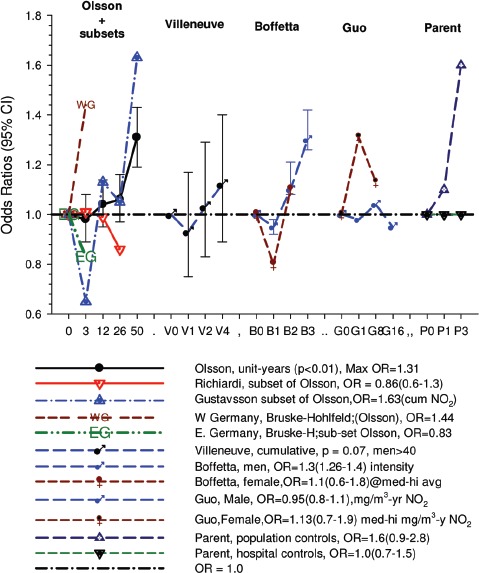
Odds ratios for the association of lung cancer and exposure to diesel emissions in population-based case-control studies in Europe and Canada (Olsson et al., 2011) including subsets from Italy (Richiardi et al., 2006), Stockholm (Gustavvson et al., 2000) and Germany (Bruske-Hohlfeld et al., 2000), Canada (Villeneuve et al., 2011); Sweden (Boffetta et al., 2001); Finland (Guo et al., 2004), and Montreal, Canada (Parent et al., 2007).

**Figure 7:**
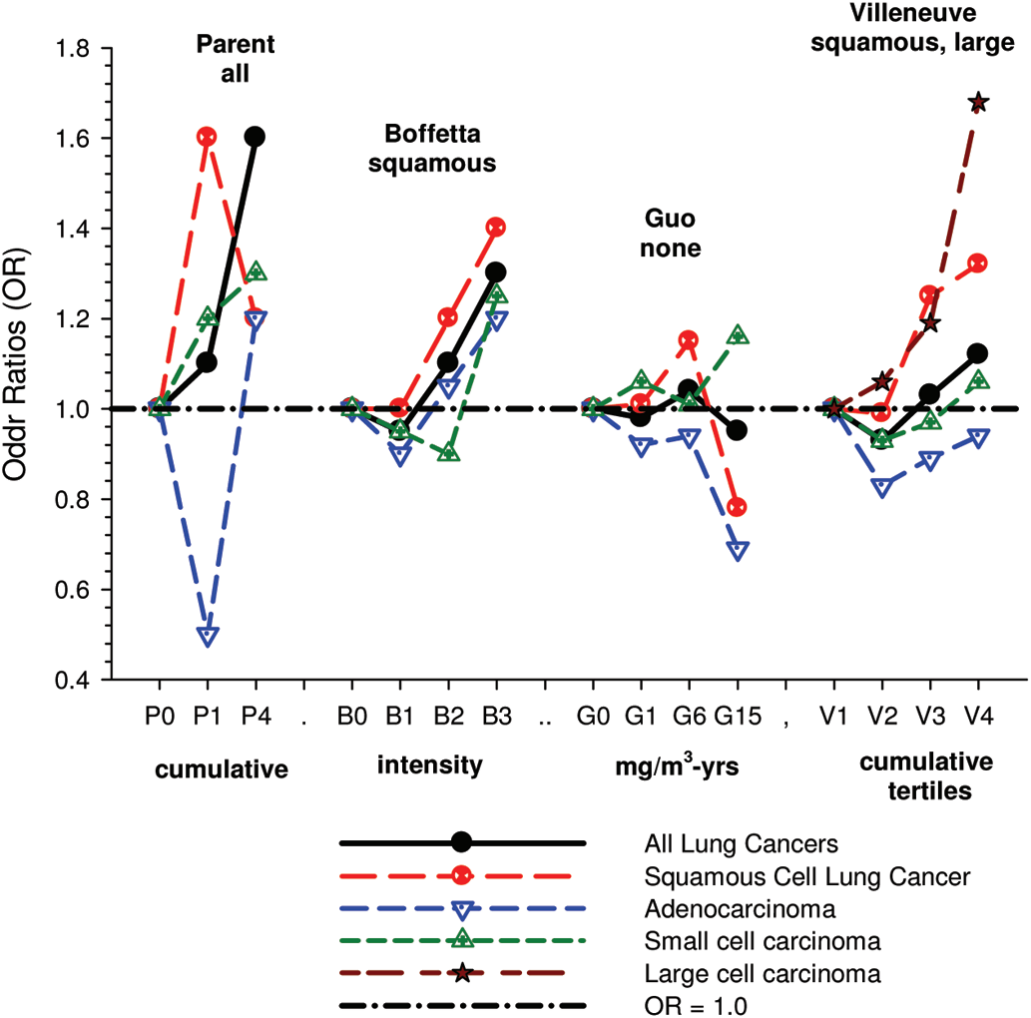
Odds ratios for the association of qualitative estimates of diesel exhaust exposure with all lung cancers and by histologic types from population-based case-control studies in Montreal, Canada (Parent et al., 2007), Sweden (Boffetta et al., 2001), Finland (Guo et al., 2004) and Canada (Villeneuve et al., 2011).
  Villeneuve et al. noted that Olsson et al. (2011) found positive E-R associations with both small cell and non-small cell lung cancers. Significant E-R trends (*p* < 0.01) and highest exposure quartiles were significantly elevated for both small and non-small cell lung carcinoma, except for non-small cell lung carcinoma among women where neither the trend nor the excess OR in the high exposure group were significant. However, the cell type categories are not the same. Comparisons with Canadian men showed among non-small cell lung cancers that adenocarcinoma (often in outer area of lung) was non-significant (*p*_trend_ = 0.60), squamous cell (usually in center of the lung next to a bronchus) was marginally significant (*p*_trend_ = 0.04) and large cell cancer (in any part of the lung) was significant (*p*_trend_ = 0.02). Small cell carcinoma was not significant (*p*_trend_ = 0.28) in the Canadian study but was significant in the pooled analysis (*p*_trend_ < 0.01).
  Villeneuve et al. also noted additional results consistent with their reported results of stronger associations with squamous cell and large cell carcinoma. Boffetta et al. (2001) was said to have “observed stronger associations between diesel exposure and squamous cell carcinoma “in a hospital-based case-control study. However, this Boffetta citation is a census study in Sweden that did not evaluate cell type and reported indeterminate E-R trends for lung cancer and DE.
  Moreover, other Boffetta diesel studies also do not suggest such an association. A 1989 hospital-based case-control study (Boffetta et al., 1989) reported ORs = 0.89 for Kreyberg Cell Type I (epidermoid or small cell or large cell cancer) and 0.83 for Kreyberg Cell Type II (adenocarcinoma) for occupations with probable diesel exposure. Another hospital-based case-control study (Boffetta et al., 1990) reported an adjusted OR of 0.95 (0.78–1.16) for probable exposure to DE. There were no analyses of risk and cell type, although 57.6% of cases had lung cancer KI and 37.5% had KII.
  In Montreal, Canada, Parent et al. (2007) did not find significant associations of diesel exposure with any cell type; ORs for squamous cell carcinoma were 1.6 (1.0–2.8) for non-substantial DE exposure, and 1.2 (0.6–2.5) for a substantial level of DE exposure.
  Siemiatycki et al. (1988) conducted a population-based case-control study in Montreal investigating associations of engine exhausts and combustion products with different types of cancer. They reported an increased lung cancer risk, particularly squamous cell cancers, associated with DE. Results were non-significant even with 90% confidence intervals with ORs of 1.2 (1.0–1.5) for squamous cell, 1.1 (0.8–1.5) for oat cell, 0.9 (0.6–1.2) for adenocarcinoma and 1.0 (0.8–1.4) for other. More detailed analysis of DE and squamous cell, including additional adjustments for confounding, showed the same overall OR of 1.2 but a wider 90% CI (0.9–1.6). Mining and quarrying was the occupation with the highest OR = 2.8 (1.4–5.8), but most of those workers had only short exposures to DE so there “may have been confounding due to some factor which was not adequately adjusted for.” There was no apparent E-R trend as substantial DE exposure had a lower OR of 1.2 (0.6–2.4) than non-substantial DE exposure at 1.9 (1.0–3.5). Siemiatycki et al. rate these results as statistically weak (non-significant even with wider 90% CI) and the inverse E-R results as evidence against an association.
  Thus, the cell type findings in this study are inconsistent with the results from the literature and do not provide evidence supporting the diesel hypothesis.
(iii) Lack of association with years worked also does not support a causative association and may be produced by adjustments for confounders. Villeneuve et al. found no (or negative) trends by years worked (Figure 4). There appears to be potential confounding that may in part explain the lack of positive E-R trends. For example:
  - When workers with “ever” exposure to gasoline were excluded, ORs were reduced in each cumulative exposure category and E-R trends of total lung cancers and cumulative exposure were reduced from *p* = 0.07 to *p* = 0.13), even though there was no E-R trend with gasoline (*p* = 0.68).
  - The observed trends are of marginal statistical significance (*p* = 0.04 and 0.02) and readily reduced to non-significance when statistically non-significant confounder exposures were excluded.
  - The E-R by years exposed is unclear. This analysis excludes men with less than 5-years exposure. The low exposure category is ≥5-years and includes the ≥10-year exposure category. With no or inverse trends, does this mean the increased risks associated with diesel exposure mostly occur in short-term workers with <5-years exposure? This again suggests a lack of any true association.
(iv) Low Participation Rates appear to bias the results. Participation rates were similar for cases and controls but low for both, at 64 and 61%, respectively. The authors point out the potential for bias and the need for cautious interpretation of the results. Nonetheless, they suggest two reasons why bias might not “fundamentally change” the results.

One proffered reason was that the observed associations with smoking were similar in direction and magnitude to the risk estimates from other studies. The second explanation offered is that potential bias might be expected due to the reduced participation of cases with more aggressive types of lung cancer (i.e., small cell lung cancer), which might contribute to risk differences by cell type. The authors argue that distribution by cell type in this study is similar to population-based distributions in North America (Wynder and Muscat 1995). And 5-year survival rates of squamous cell, adenocarcinoma, and large cell subtypes are similar (Gloecker Ries and Eisner 2007). As a result, the authors assert that, “participation bias is an unlikely explanation for the stronger associations observed with squamous cell.”

But the observed stronger associations with squamous cell cancer does not provide convincing evidence supporting the diesel hypothesis as no biologically plausible reason for squamous cell susceptibility is provided and a stronger association for squamous cell is inconsistent with the diesel literature depicted in Figure 7.

On the other hand, potential bias may well occur because of a biased sample of controls, which is the point raised by Mohner (2012) with regard to low participation rates in the pooled study (Olsson et al., 2011). The potential positive bias in this instance centers on several interrelated factors, including higher exposures to DE and other occupational hazards among manual workers who tend to have lower education and lower response rates.

In this study the distribution of cases and controls indicates that controls have higher incomes and more education than cases, while cases are heavier smokers with many fewer non-smokers than controls (their Table 2). These differences again indicate that controls are not representative of cases in terms of income, education and smoking which may bias results because of the reduced risk of lung cancer associated with higher income and education and reduced smoking (Mao et al., 2001). While smoking adjustments remove a positive confounding effect, adjustments for the positive confounding effects of income and education might further reduce the lung cancer risk. The magnitude of these potential biases is not known, but the higher incomes and education among controls compared to cases will likely bias the OR upward.

### 3.5 Summary

This is a well-conducted study that adjusts for potential occupational and non-occupational hazards (e.g., silica, asbestos, cigarette smoke). There are no apparent associations of gasoline emissions with lung cancer. There also are no apparent associations of diesel emissions with all cases of lung cancer, but ORs for squamous cell and large cell carcinomas are excessive at high DE exposures. It is not clear why these cell types would show a greater risk than, say, small cell carcinoma. In addition, there is potential residual confounding from misclassification of diesel exposure, which should be ameliorated by the expert exposure assessment used in this study. Low participation rates may bias risks upwards because of the differential natural selection of more highly educated controls. A reviewer recommended an analytical strategy that focuses on testing for associations with lung cancer per se, and cautioned against proceeding to further analyses if the initial results are not significant. If further analyzes are conducted (e.g., by cell type) then the issues of multiple testing, including bias and chance, become material concerns.

## 4. Summary of population-based studies

The two recent population-based case-control studies (Olsson et al., 2011; Villeneuve et al., 2011) are similar in design to the previously reviewed (Gamble 2010) studies (with qualitative exposure estimates) of populations in Montreal, Canada (Parent et al., 2007), Turin, Italy (Richiardi et al., 2006), and Stockholm, Sweden (Gustavsson et al., 2000); and two studies using census data in Finland (Guo et al., 2004) and Sweden (Boffetta and al 2001). Overall there are five studies at issue, since an Italian (Richiardi et al., 2006), a Swedish (Gustavsson et al., 2000) and combined German studies (Bruske-Hohlfeld et al., 1999) were incorporated into the Olsson et al. study (Olsson et al., 2011) (Figure 6).

There appears to be a fairly consistent pattern of positive E-R trends, which is complemented visually by the larger ORs in the highest exposure groups and negative or flat trends at lower exposures. Several studies had separate E-R analyses for men and women (Boffetta and et al., 2001; Guo et al., 2004) and one study had two sets of controls, population and hospital-based (Parent et al., 2007). In the analyses by sex, the Swedish men showed a significant association but Swedish women did not (Boffetta and et al., 2001), while in the Finnish study (Guo et al., 2004) neither sex showed a significant association. In the Montreal study (Parent et al., 2007) population controls showed a significant association but hospital controls did not.

A primary strength of population-based case-control studies is they consistently adjust for potential life-style confounders (e.g., smoking, education, SES). A primary limitation is their exposure assessment methods. Information on job history is second-hand (such as from relatives) and exposures are based on expert opinion using occupational or job titles often without exposure measurements or first-hand knowledge of individual workplace conditions. They are ecological assessments where a particular job is rated with the same exposure for all participants, often irrespective of time (which can determine the presence or absence of diesel engines) or place.

Other limitations of these studies relate primarily to the fact that a majority of subjects were not exposed to DE 20 or more years before death, thereby making it improbable that diesel emissions are causally related to lung cancer. It is also difficult to compare results because exposures are qualitative and, therefore, not comparable quantitatively.

The search for patterns reveals great heterogeneity and little convincing evidence with regard to the lung cancer-DE hypothesis (Figure 6). We summarize our conclusions based on the current reviews of Olsson et al. and Villeneuve et al. (Olsson et al., 2011; Villeneuve et al., 2011) and past review (Gamble 2010) of the other studies.

(i) Boffetta et al. (2001) is a Swedish census study with indeterminate results regarding a causal association because:
  - Contrasting trends of men and women detract from the diesel-lung cancer hypothesis.
  - Lack of information on potentially confounding exposures (smoking, SES, silica, asbestos) that could change E-R trend as occurred in Finland (Guo et al., 2004) and lack of years worked cast additional doubts on any reported trends.
  - Older workers diagnosed before 1980 probably had less than 20-years after initial diesel exposure, but years worked is unknown so the actual latency could not be determined.
(ii) Guo et al. (2004) is a Finnish census study that tends to distract from the lung cancer-diesel hypothesis because:
  - The E-R trend is negative for men and non-significant for women, and the lack of consistent increases in risk by job or cumulative NO_2_ exposure led the authors to conclude that “occupational exposure to engine exhausts was not consistently associated with lung cancer in this study.”
  - Exposure misclassification may be reduced because of prospective exposure sampling by job since 1945, but the job matrix is still unable to assess DE exposure for individual jobs.
  - The probability of diesel exposure is less than 50% up to 1984, which time period is well within the 1971–1995 follow-up, indicating that a majority of workers had no diesel exposure. It was not possible to determine who actually had diesel exposure and who did not.
(iii) Parent et al. (2007) is a community-based case-control study in Montreal that the authors concluded offered limited evidence for the DE-lung cancer hypothesis because:
  - Associations were weak and inconsistent, with only one statistically significant association in the high concentration group with population controls.
  - A strength of this study was the recognition of the transition from gasoline to diesel engines with a fair number of jobs having less than a 50% probability of exposure. Because DE had to be assigned on the basis of probability (ecological or group assignments were necessary), some exposure misclassification occurred. The late introduction of diesel engines into the workplace indicates latency was inadequate for some workers.
(iv) Villeneuve et al. (2011) were part of the research group conducting the study by Parent et al. (2007). The strength of this study is the exposure assessment and the attempt to adjust for the transition from gasoline to diesel engines in the workplace. Nonetheless, this study provides little support for the lung cancer-diesel emissions hypothesis because:
  - The lack of significant E-R trends for all lung cancers detracts from the diesel-lung cancer hypothesis (Figures 3, 4).
  - The participation rate is low, and the control group appears weighted toward a reduction in workplace exposures and a potential upward bias toward spuriously higher ORs.
(v) Olsson et al. (2011) is quite a large study in terms of number of cases, investigators, and geographic area. Still, this study provides little support for the lung cancer-DE hypothesis because:
  - The large size may have contributed to a limitation of exposure assessment where a job matrix was used without consideration of time or place when ranking exposure. With all job titles ascribed the same rating, there is potentially substantial exposure misclassification.
  - The misclassification is unlikely to be non-differential as jobs associated with the early introduction of diesel engines in the workplace will be misclassified less frequently than jobs where diesel came into the workplace more slowly and later in time, and the time frame for changes may differ from country to country. The Italian study contributed about 8% of the cases, and the published results do not support the lung cancer-DE hypothesis because of the lack of E-R trends (Richiardi et al., 2006). This study appears to have more adequate latency than many studies. The evidence supporting differential DE exposure misclassification is observed in meta-analyses published before 2000, which concluded that the evidence supported a causal association between lung cancer and DE exposure (Bhatia et al., 1998; Lipsett et al., 1999). But the evidence for this conclusion was based on studies without adequate latency, and exposure misclassification occurred because workers were classified “exposed” when they were more likely “non-exposed” to DE because of the relatively rare occurrence of DE in the workplace (see Table 6 in Gamble, 2010).

**Table 6:** Summary of exposure-response slopes and HRs for underground (UG) workers using untransformed and log transformed regression models over full cumulative exposure range for unlagged and 15-year lags (Table S7) (Attfield et al., 2012).

	Untransformed HR (95% CI), *p*, HR at 1000 → 4000 REC	Log transformed HR (95% CI), *p*, HR at 1000 → 4000 REC	Untransformed <1280 REC HR (95% CI), *p*, HR at 1000 → 1280 REC, Exclude <5-years tenure
Un lagged	1.01 (0.89–1.14), (*p* = 0.89), 2.7 → 57 (35–96)	1.15 (1.0–1.31), (*p* = 0.046), 9.2 → 11.4 (8.3–16)	
15-year lag	1.03 (0.83–1.28), (*p* = 0.12), 2.8 → 62 (28–165)	1.07 (0.97–1.19), (*p* = 0.08), 7.9 → 9.6 (7.8–12.4)	4.06 (2.1–7.8), (*p* = <0.001), 58 → 181 (15–21676)

These are primary results for authors' conclusions following the procedures outlined in the protocols (NCI/NIOSH 1996; NCI/NIOSH 1997). UG workers with restricted exposure range (<1280 µg/m^3^-years) and excluding workers <5-years tenure (Figure 11 or 15) are included for comparison to demonstrate biased results produced by this *a posteriori* analysis.
  - Many jobs have inadequate (too short) latency periods.
  - Potentially large proportions of jobs are misclassified regarding DE exposure because job histories began in the 1920s, and even into the 1970s a large proportion of jobs did not involve DE.
  - Risk by job did not correlate well with the presumed levels of DE associated with that job, which detracts from the observed E-R association.
  - The Swedish study (Gustavsson et al., 2000) contributed about 8% of the cases, and the authors concluded that, at cumulative NO_2_ exposures around 5.5 mg/m^3^ years of exposure increased the risk of lung cancer and thus supported the lung cancer-DE hypothesis. The previous review (Gamble 2010) suggests that those results are indeterminate because: possible downward biases in exposure estimates produce upward bias in risk; crude sensitivity analyses suggest that adjustments for bias reduce ORs to a null value; inadequate latency among retirees over 65 years; and much higher risks than reported in the Finnish study (Guo et al., 2004) which had much higher NO_2_ exposures but no increased lung cancer risk.

There also were inconsistent patterns in the analyses by histological cell type (Boffetta et al., 2001; Guo et al., 2004; Parent et al., 2007; Villeneuve et al., 2011) (Figure 7). Stronger associations with squamous and large cell carcinomas were observed in the Canadian study (Villeneuve et al., 2011) and may have been considered evidence that supported the lung cancer-DE hypothesis. However, cell type data from other studies provide little support for a consistent pattern of association with any cell type (Figure 7).

## 5. NCI/NIOSH cohort mortality study of non-metal miners: Attfield et al. (2012)

### 5.1 Description

This is a cohort of 12,315 workers with at least 1-year of diesel exposure in eight non-metal mining facilities in US (one limestone, three potash, one salt and three trona). End of follow-up was December 31, 1997. Diesel engines were introduced in the mines during the period 1947–1967; the individual range of first exposure was 1947–1996; and the average range by mines was 1967– 1976. Personal samples were collected in the period 1998–2001.

E-R analyses used respirable elemental carbon (REC) as the metric for cumulative (µg/m^3^ years) and average intensity (µg/m^3^) of exposure. Surrogate estimates of REC levels were based on extrapolations from CO sample data and presumed DE-related determinants, including diesel engine horsepower and ventilation rates (Coble et al., 2010; Stewart et al., 2010; Vermeulen et al., 2010a,b). Estimated exposures were developed for potential confounders including silica, radon, asbestos, non-diesel PAHs, and respirable dust with semi-quantitative values 0–3 assigned to silica and asbestos exposures. Separate analyses were by worker location: surface only and ever underground (UG). For the analyses there were 200 cases where lung cancer was the underlying cause of death (COD) and 212 cases where lung cancer was seen as contributing to the COD.

The E-R analyses used a series of exposure metrics:

- Categorical: (i) quartiles using *n* lung cancers as cutpoints; and (ii) categorical analyses using expanded cutpoints 2, 4, 8 …… 128 μg/m^3^. The expanded cutpoints were from “15-year lagged average REC intensity (where the REC level of the least exposed surface workers formed the basis for the reference category, with a doubling in exposure level thereafter).”
- Continuous: adjusted for confounders: (iii) continuous regression analyses used cumulative and average REC; (iv) two secondary (*a posteriori*) analyses “based on the patterns of data” were added for UG workers analyses; (iva) exposure was restricted to <1280 µg/m^3^ year to “improve the characterization of the E-R trend at the lower and middle sections of the cumulative REC exposure range;” and (ivb) log transformed power models were added to accommodate leveling-off of E-R at highest exposure levels.
- Other metrics: exclusion of workers with less than 5-year tenure was used to account for the differential mortality pattern among short-term workers. This is *a posteriori* exclusion that was not in the protocol nor used in the case-control analyses. It is stated in the methods section that a criterion for inclusion into the cohort was employment for at least 1-year after dieselization at the study facility, presumably to account for differential mortality of short-term workers. Thus, the subsequent selection of less than -year employment after analyses of other short-term employment exclusion periods (i.e., <2-, <5- and <10-years employment) was made *a posteriori*.

### 5.2 Results

The only significantly increased SMRs were for lung cancer with an SMR of 1.21 (1.01–1.45) among UG workers (*n* = 122 cases and 8.8% mortality) and 1.33 (1.06–1.66) among surface workers (*n* = 81 cases and 10.2% mortality). If DE is increasing the risk of lung cancer, the slightly higher mortality for surface workers compared to UG workers is highly unexpected based on the much larger exposures of UG workers.

The estimated mean REC exposure for UG workers was 75 times higher UG at 128 (126–130) μg/m^3^ than for surface workers at 1.7 (1.6–1.7) μg/m^3^; respirable dust was 2.9 times greater for UG workers at 1.93 (1.91–1.93) μg/m^3^ versus 0.67 (0.67–0.68) μg/m^3^ for surface workers. Estimated average exposures for potential confounding exposures were low, with no differences between UG and surface workers for asbestos and non-diesel PAHs. Silica was 1.3 times higher for UG workers, and radon was 0.011 working level vs. 0 working level. There was some evidence of a radon effect in Mine A for UG workers with more than 40 years employment and hire dates before 1947.

#### 5.2.1 Exposure-response

Cumulative exposure (µg/m^3^ years) to REC was considered the most relevant exposure metric for chronic disease, and will be the focus of this review. In this section we will consider two sets of E-R results. Results as presented in the mortality study are difficult to follow since they are comprised of intermingled *a priori* and *a posteriori* analyses in the same models, forcing the reader to look for only *a priori* results, or a facsimile of their *a priori* results in their supplementary tables (NCI/NIOSH 1996; NCI/NIOSH 1997). Therefore, we have taken the unusual step of summarizing our interpretation of the authors’ primary results. These results will be followed in the next section with our summary interpretation of *a priori* results, largely from their supplementary tables. Note that at the beginning of the Cox E-R analyses the authors comment: “Initial (i.e. *a priori* defined) analyses from the complete cohort did not reveal a clear relationship of lung cancer mortality with DE exposure” as different patterns of lung cancer mortality between surface and UG workers “had obscured exposure-response in the complete cohort.” Therefore numerous subsequent analyses adjusted for worker location were undertaken.

We are concerned with the large number of models and determinations of statistical significance. As the number of tests increases, the true meaning of the statistical significance level (*p*) begins to lose any meaningful interpretation. This is particularly true of the *a posteriori* analyses where the data led the investigator to “better” or “more significant” models. This point is discussed further in Section 5.4.7.

E-R results are presented as categorical models and continuous regression models. Our plots of hazard ratios in this review that refer to the continuous model are based on two assumptions. The first assumption is that the hazard function is described by the equation near line 285 of the online version of the paper:


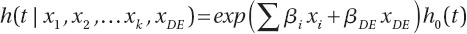


where the DE subscript refers to the exposure. From this equation we can develop the hazard ratio as exp(β_DE_χ _DE_). The second assumption is that the hazard ratio (HR) values associated with the continuous models in Tables S4, S5, S6 in the main paper (and similar tables in their Supplement) represent the term β_DE_.

Based on these two assumptions we develop the HR plots from exp(β_DE_χ _DE_) for the untransformed exposure model, and from (χ_DE_)^β^^DE^ or the log transformed exposure model. If the assumptions are correct, this type of plot represents the E-R for the hazard ratio not considering confounders. An interpretation of the differences between the continuous model plots and the categorical result plots represents the effect of the confounders. The categorical models include the effect of the confounders – the categorical are based on observed data and include exposure and confounders. The full statistical model includes a term for exposure and terms for confounders. But they have only presented the HR, which is only the exposure effect term so full model results have not been provided. Without the confounder terms we cannot plot the full model with confounders.

This interpretation presents an anomaly between the untransformed and log transformed models. For the UG workers (their Table 4), the untransformed model is not statistically significant (*p* > 0.05) and the log transformed model is statistically significant *p* < 0.05). Since the form of the HR is (χ _DE_)^β^^DE^ and the exposure is in 1000 µg/m^3^ year, then the HR will be below 1 for all exposures less than 1000 µg/m^3^ year in log transformed models. For surface workers (their Table 5) the significance is reversed (the untransformed model is statistically significant and the log transformed model is not statistically significant) so the HR is greater than 1 for all exposures greater than 0. Thus, the continuous models do not seem to present a reasonable or consistent biological pattern.

**Table 5:** Summary of exposure-response slopes and HRs for surface and underground (UG) workers using untransformed log-linear and log transformed regression models over full exposure range and restricted exposure range (<1280 µg/m^3^ years) for UG cohort for workers with 15-year lags and excluding workers with <5-year tenure (Attfield et al., 2012).

	HR slope (95% CI) per µg/m^3^ years, HR at highest µg/m^3^ years in exposure range		
	
	Surface	UG	UG
Exposure range (µg/m^3^ years)	0–160	0–2000	<1280
Untransformed log-linear model	1.02 (1.0–1.03), 23.8	1.0001 (0.99–1.0003), 1.14	1.0014 (1.0007–1.002), 6.01
Log transformed	1.03 (0.75–1.4), 1.07	1.19 (1.04–1.37), 1.78	–

Primary results for authors' conclusions.

The hazard ratio values from both models (untransformed and log transformed) presented in the Tables are statistically significant, but result in different response patterns for a given data set, as seen in the subsequent figures. In Section 5.2.1.1 we will summarize primary results on which the authors focused and based their conclusion. The discussion and plots, while intricate, describe the rationale for the authors’ conclusions. In Sections 5.2.1.2 and 5.2.1.3 we will present, using a similar description, our view of what the primary results should be and why we are taking this unusual set of descriptive steps.

##### 5.2.1.1 Primary results from author's perspective

**Figure 8:**
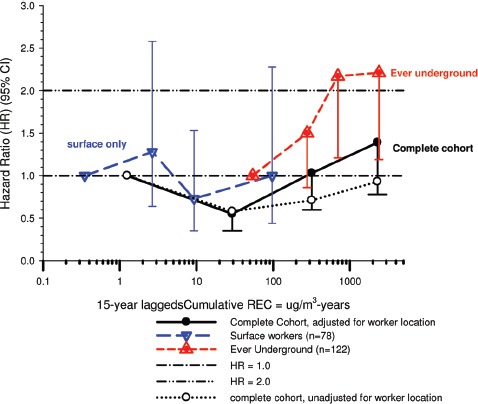
Proportional Hazards ratios on lung cancer mortality for 15-year lagged REC cumulative exposure of surface worker, UG workers and the complete cohort adjusted and unadjusted for worker location in DEMS cohort study (Tables 4, 5, 6 in Attfield et al, 2012).

**Figure 9:**
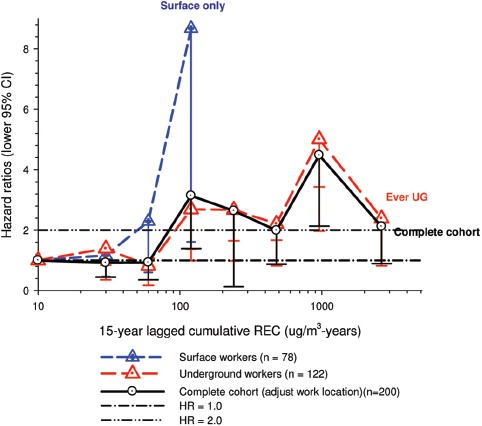
Proportional hazard ratios (HR) on lung cancer mortality for 15-year lagged REC cumulative exposure of surface workers, UG workers and complete cohort expanded categories excluding workers with ≤-years tenure, Tables 4, 5, 6 in Attfield et al. (2012).

**Figure 10:**
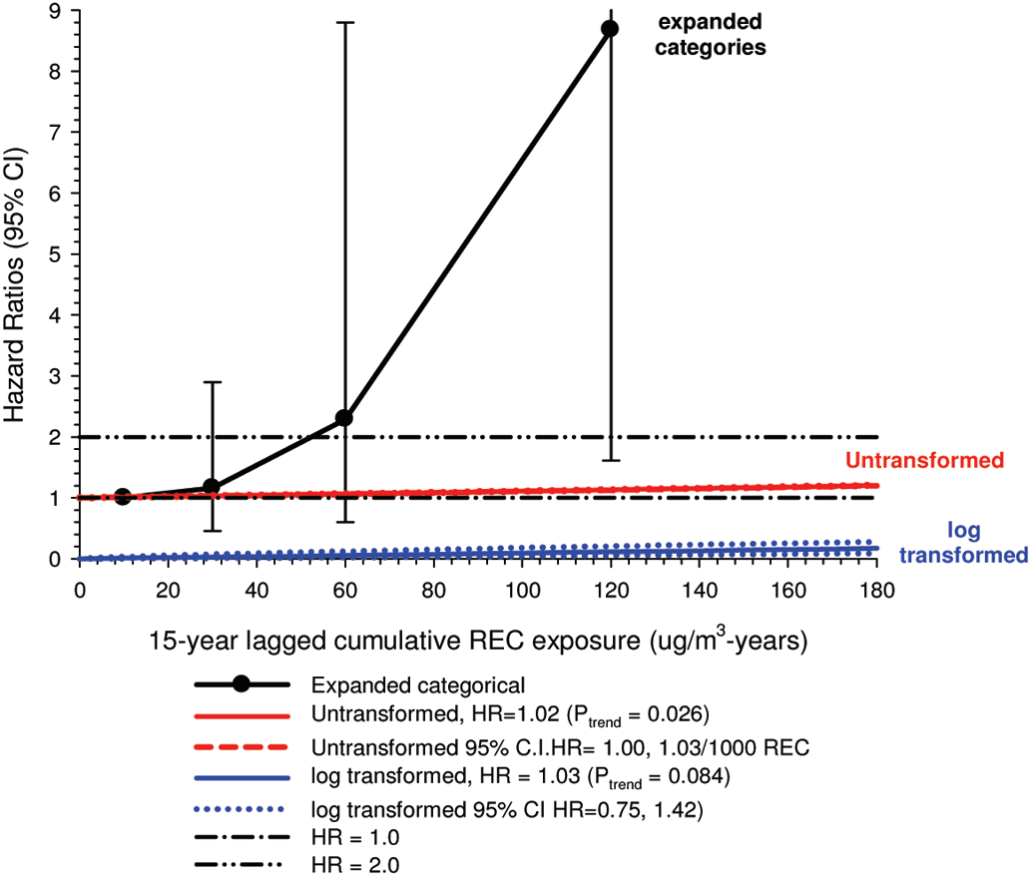
Proportional hazard ratios for lung cancer and cumulative REC among surface workers with 15-year lags and exclude workers with <5-year tenure in expanded categories, untransformed and log transformed regression models, Tables 5 and S8 from Attfield et al. (2012).

**Figure 11:**
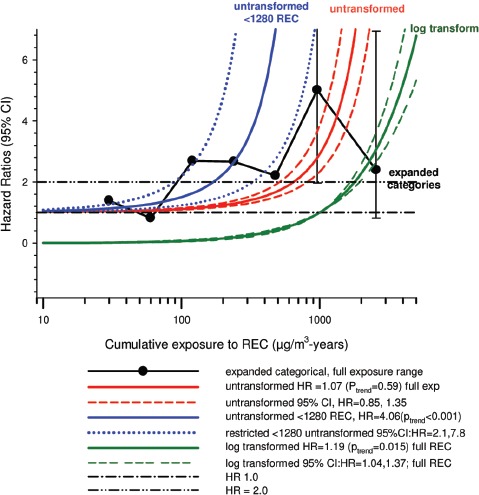
Proportional hazard ratios (HR) on lung cancer mortality for 15-year lagged REC cumulative exposure in UG workers excluding workers >5-years tenure; Expanded categorical model plus log-linear regressions for full and restricted exposure range <1280 μg/m^3^-years; log transformed model with full exposure range (Table 4) (Attfield et al., 2012).

The initial *a priori* analysis of the complete cohort without adjustment for worker location did not show an E-R trend (dotted line in Figure 8). Subsequent analyses stratified by worker location then resulted in no trend for surface workers, a clear trend for UG workers, and a nonsignificant trend for the complete cohort when adjusted for worker location (Figure 8). The subsequent analyses focused on 15-year lagged REC exposures and exclusion of workers with less than 5-years tenure (Figures 8–11).

Figure 9 shows expanded categorical analyses of the complete cohort – UG and surface workers with 15-years lags and excluding workers with less than 5-years exposure. Exclusion of workers with shorter tenure was introduced because the resultant E-R trend was “more pronounced.” The total cohort is adjusted for workplace location and is quite similar to the UG worker pattern.

Surface workers show a steep E-R pattern with two-fold and significant 8.7-fold increased HRs in the two highest expanded exposure categories of 40–80 and 80–160 µg/m^3^ years with four and two lung cancer cases respectively (15-year lags, exclude workers <5-year tenure). The untransformed E-R slope for surface workers is 1.02 (1.00–1.03)/µg/m^3^ year (*p* = 0.03). The log transformed slope has a slope of 1.03 (0.75–1.42) (*p* = 0.84). Both continuous regression models show flat slopes and log transformed HRs are <<1.0 as REC is ≤180 µg/m^3^ years (Figure 10).

The complete cohort and UG workers show similar expanded category E-R trends, so E-R trends for the complete cohort and UG workers should be similar (Figure 9). Among UG workers, HRs are elevated in the last 5 cumulative exposure categories (>80 µg/m^3^ years). They are significantly increased in the penultimate exposure category [HR = 5.04 (1.97–12.8)] but decline in the last work category to 2.39 (0.82–6.9), which is similar to work categories 4–6.

Among UG workers and over the full exposure range none of the E-R trends from the continuous models fit the expanded categories (Figure 11). The continuous models have similar shapes rising above ORs of 2 at about 2000 and 600 µg/m^3^ years for the log transformed and untransformed models. The restricted model (restricted to exposures less than 1280 µg/m^3^ years) reaches OR = 2 at 200 µg/m^3^ years before ascending nearly straight upward.

Excluding workers with more than 1280 µg/m^3^ years produces a highly significant (*p* < 0.001) model with an E-R slope of 4.06 (2.11–7.83) per 1000 µg/m^3^ years (or 1.0014), and is the only significant model after exclusion of high exposures and workers with <5-years tenure. These continuous models are not biologically plausible and do not follow the expanded category model trends, and for most of the exposure range are either below or above the ORs of the expanded categories. And the least significant untransformed model visually fits the categorical model better than the more statistically significant log transformed and restricted models (Figure 11).

The surface workers' E-R pattern shows much higher HRs and steeper E-R slopes than for the UG workers despite the much lower exposures of the surface workers. In the expanded categories the maximum HR of 8.68 (1.61–49.9) among surface workers is nearly twofold higher than the maximum HR of 5.01 (1.97–12.76) among UG workers at 640–1280 µg/m^3^ year in the 7th exposure category of the expanded categorical analysis (their Tables 4 and 5). This HR of 5.01 is the highest HR in any of the expanded category models, indicating the strong effect of the *a posteriori* analyses, which excluded all workers with less than 5-years tenure:

**Table:** 

	15-year lags		Unlagged	
HR at penultimate	Exclude	No	Exclude	No
Exposure category	<5-year tenure	exclusions	<5-year tenure	exclusions
	5.01	2.42	4.09	2.62

The authors' conclusion is based on workers with 15-year lags, exclusion of workers less than 5-years tenure, and cumulative exposures restricted to <1280 μg/m^3^ years for UG workers. These same restricted data provide the weight of evidence for their specific finding of “an increased risk of lung cancer in both underground and surface workers” associated with DE exposure (Figures 9–11, Table 5).

##### 5.2.1.2 Primary results from reviewers’ perspective

Our review of the E-R results is based on the following considerations:

(i) The exposure range restriction of excluding exposures greater than 1280 µg/m^3^ years is an *a posteriori* addition not outlined in the protocol. It was added to the analysis after finding the lack of significant E-R trends from *a priori* defined regression models. Restriction of the exposure range is considered an exploratory descriptive exercise where statistical significance is generally considered to be meaningless. A *posteriori* analysis should be interpreted differently from *a priori* results, and should not contribute to the interpretation, conclusions and weight of evidence regarding the lung cancer – diesel hypothesis.
(ii) Results from unlagged and 15-year lags may be exploration of the data preparatory to E-R analyses as the protocol indicated “lagged estimates of exposure will be explored” (NCI/NIOSH 1997). We consider using goodness of fit as a criterion to select the ‘best’ lag (or to justify it) is profoundly wrong: this choice should be made *a priori* and should be biologically driven. An exploratory, data-driven approach may be justified if a causal association exists and underlying biologic mechanisms need to be investigated. To give preference to the results of *a posteriori* analyses without a strong rationale for it (as exemplified by the sentence in the protocol ‘exploratory analyses’) is in our opinion conceptually wrong.
  Both unlagged and 15-year lagged data were presented and 15-year lags tended to be favored. A 15-year lag period cuts off the last 15 years of exposure because they are thought unlikely to have any effect on lung tumor development. This gives emphasis to a possible DNA-damaging effect of DE: indeed DE contains PAHs and nitro-PAHs which are likely to be genotoxic. However, as for other complex mixtures – namely tobacco smoke – other carcinogenic mechanisms are also plausible, including those involving chronic inflammation. If inflammation is a causal driver of lung cancer the last 15-years of exposure are probably important to maintain the inflammatory response. If the mechanism is similar to that of smoking, unlagged or shorter lags would seem to be consistent with the rapid declines in lung cancer risk following cessation of smoking. Therefore we cannot exclude the possibility that recent exposure to DE might be linked to cancer risk.
  Both lagged and unlagged analyses are commonly found in occupational epidemiology. We find the use of a 15-year lag to be an acceptable criterion based on common usage, but would prefer biological reasons rather than model fit or statistical significance as reasons for use and selection of particular lag periods. Inconsistencies in results from lagged and unlagged analyses, both internally and between cohort and case-control studies, cast further doubts on the analyses.
(iii) Workers with less than 1-year diesel exposure were excluded from the total cohort as outlined in the protocol. The authors added further analyses excluding several categories of “short-term” workers with longer exposures (<2-years, <5- and <10-years). They reported that as tenure exclusions increased, progressively higher HRs was produced. Exclusion of less than 5-years exposure was selected by the authors specifically to maximize HRs and minimize loss of power. This data exploration was not included in the protocols as short-term worker effects were presumably accounted for by only including workers with more than 1-year tenure. Added exclusions should not be part of the regular results and it is inappropriate to attach statistical significance to any of these analyses. Our conclusion is supported by the HEI statement (Bailar et al., 1999) that such added analyses may supplement the primary results but “lack full statistical justification” and may “bias the results.” In fact, these added analyses excluding workers with less than 5-years tenure were not supplements to primary results; they became the core of the primary results.
(vi) We consider the added analyses to be outside the realm of primary data and have not used them for our interpretation or conclusions because, in summary:
  - Only short-term workers with less than 1-year exposure were *a priori* selected for exclusion.
  - Analysis of additional exclusions was not part of the protocol, are *a posteriori* selections based on maximum estimated risks and minimal loss of subjects, and therefore potentially biased.

Our focus will be on the total cohort as this was the plan outlined in the protocol and accomplished in the nested case-control study. The obfuscation of the E-R trend for the total cohort led to separate analyses of surface and UG cohorts. This was unnecessary as the positive E-R trend among surface workers disappeared when adjustment was made for worker location as noted by the authors and observed in Figure 9. Thus it is unclear why the focus off the primary results turned onto the UG workers separately instead of focusing on the total cohort as in the case-control study.

Regression results for the total exposure range are generally not reported, which is considered a limitation and eliminates direct comparisons with the total cohort case-control results.

##### 5.2.1.3 Primary results from reviewers' perspective

**Figure 12:**
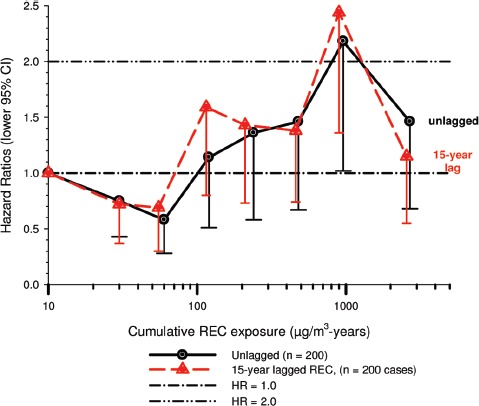
Proportional hazards ratios (HR) for lung cancer by 15-year lagged and unlagged REC cumulative exposure (μg/m^3^-years) for the complete cohort adjusted for worker location using expanded categorical cutpoints (Tables S5 and S6) (Attfield et al., 2012).

**Figure 13:**
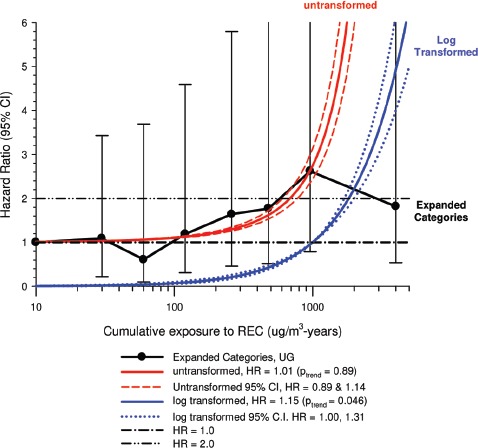
Proportional hazards ratios for lung cancer mortality by unlagged REC cumulative exposure (μg/m^3^-years) among Underground workers with expanded categories, untransformed (HR = 1.01) and log transformed regression (HR = 1.15) models for UG workers from Tables S6 and S8, Attfield et al. (2012).

**Figure 14:**
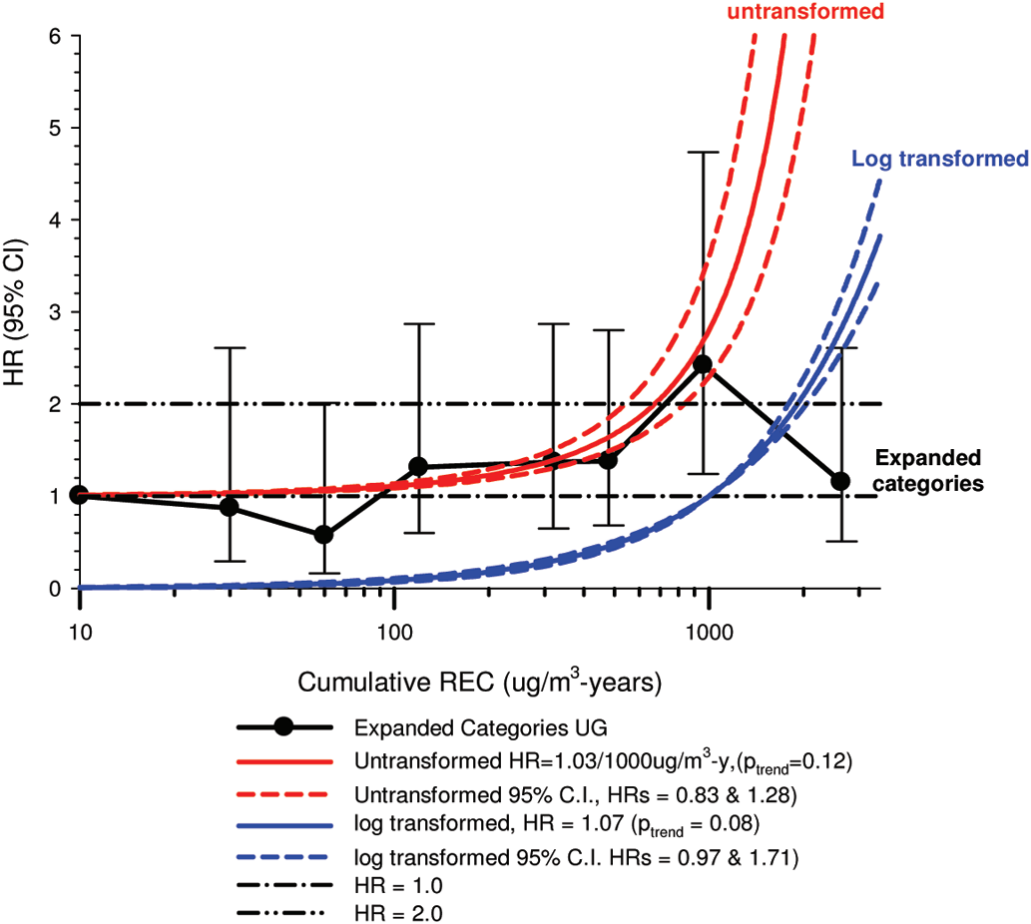
Proportional hazards ratios (HR) for lung cancer among underground workers by 15-year lags REC cumulative exposure (μg/m^3^-years) in expanded category untransformed (HR = 1.03/1000 μg/m^3^-year) and log transformed (HR = 1.07) regression models from their Tables S5 and S7 in Attfield et al. (2012).

We consider many of the results in Figures 9–11 to be potentially biased sub-group analyses because of the exclusion of workers with <5-years tenure and the restriction of the exposure range to <1280 µg/m^3^ years. These sub-group analyses appear to have been conducted after sifting through exploratory data from analyses not outlined in the protocol. These data (presented in their text Tables 4–6) may be useful for descriptive purposes, but p-values are inappropriate for *a posteriori* analyses (Bailar, Gilbert et al., June, 1999) and it is not clear how they can be used for interpreting this study (Figures 12–14).

We will now review the available data that largely adhere to the analyses outlined in the protocol. Since E-R slopes for the complete cohort were not reported for the entire exposure range, UG workers will be surrogates for the total cohort. We summarize 15-year lags and unlagged exposures for the entire exposure range and without exclusion for tenure.

There are 200 lung cancer cases in the total cohort of surface plus UG workers without exclusions. There are 55 cases in the unlagged referent group and 85 in the lagged analysis. Figure 12 shows E-R trends in the total cohort for unlagged and 15-year lags and expanded categorical cutpoints and adjusted for work location. E-R trends are similar. Both show a decline in the slope in the first three exposure category (<80 µg/m^3^ years). Both show possible plateaus with significant >two-fold increased HRs in the penultimate exposure category, and declines in HRs to <1.5 in the last exposure group.

We will now review the lagged and unlagged data using expanded categorical analyses for the total cohort for comparisons with UG workers where continuous model results are available. The pattern of E-R trends is quite similar as lags have little effect on the results as observed in Figure 12.

There are 122 cases in the UG cohort. There are three cases in the unlagged referent group (0–20 µg/m^3^ years) and 20 cases in the 15-year lagged cohort. Figure 13 shows unlagged E-R trends for UG workers using expanded categorical cutpoints and untransformed and log transformed regression models (from their Tables S6 and S7). The maximum HR is 2.62 (0.79–8.72, *p* = 0.12) in the penultimate exposure group (640–1280 µg/m^3^ years) of the UG workers. The untransformed regression slope is highly non-significant (*p* = 0.89) (HR = 1.00001) but has a similar shape as the log transformed model which is marginally significant (*p* = 0.05) with a slope of 1.15 (1.00–1.31). A major difference is the HRs at zero exposure (1.0 and 0) and the exposure level where the HRs = 2.0, which are 1000 and 2000 µg/m^3^ years REC for untransformed and log transformed models, respectively. The untransformed regression model is a relatively good fit with the expanded category model up to the penultimate exposure category around 1280 µg/m^3^ years, but then it veers upward while the HR declines in the categorical model. The lower CIs of the log transformed model are outside the lower 95% CI of the categorical model. The only significant model (log transformed) is the least biologically plausible with ORs <1.0 below 1000 µg/m^3^ years REC. Thus, these unlagged models show no apparent association between lung cancer and DE exposure (Figure 13).

Figure 14 shows E-R trends for UG workers with 15-year lags using expanded categories, untransformed and log transformed regression models (from Tables S5 and S7). The maximum HR is 2.42 (1.24–4.73, *p* = 0.01) in the penultimate exposure group. As in the unlagged UG cohort analyses (Figure 13), the untransformed regression model fits the categorical model well up to the final exposure category (*p* = 0.12), while the log transformed regression does not fit the categorical data at all (*p* = 0.08). The lagged categorical model is the most biologically plausible model but shows no statistically significant E-R association between lung cancer and 15-year lagged cumulative REC (Figure 14).

**Figure 15:**
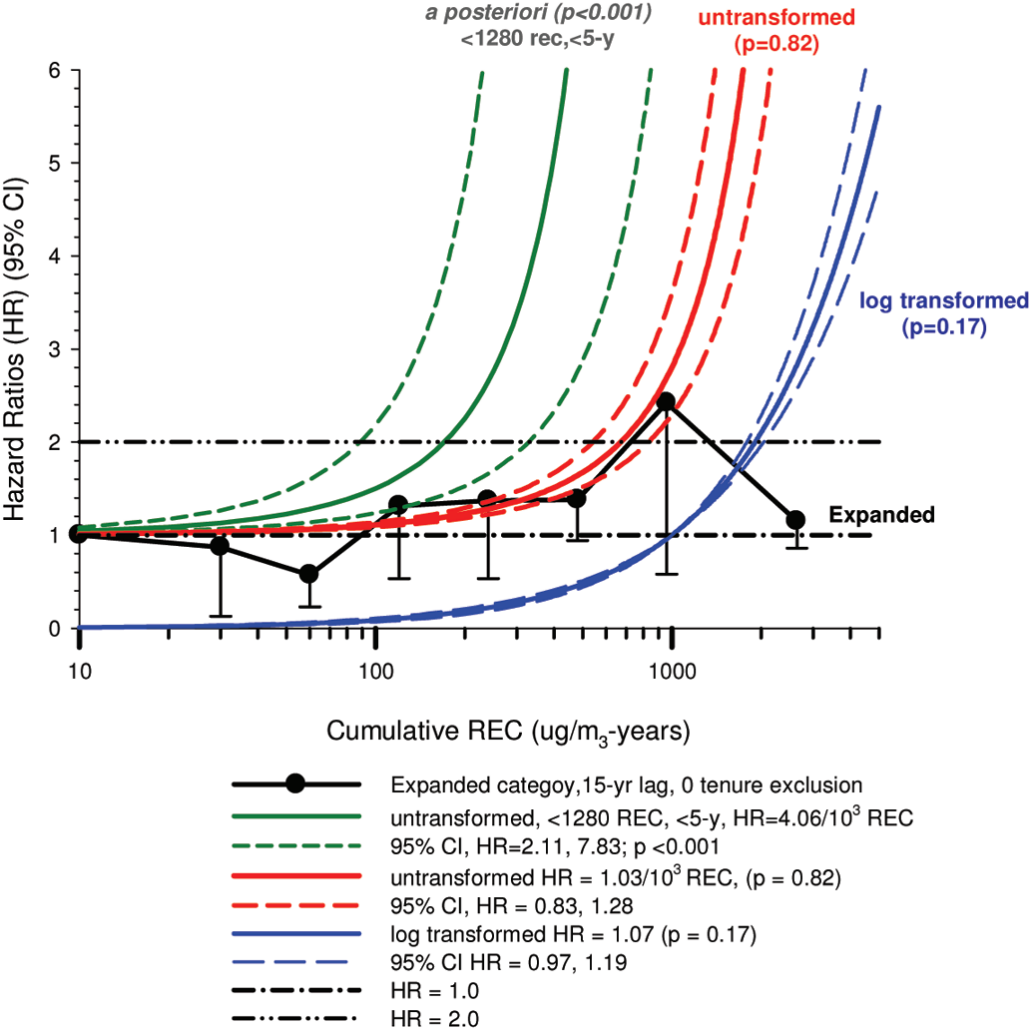
HR for lung cancer and 15-year lagged cumulative REC for UG workers in expanded categories as referent: Attfield primary results = untransformed HR=4.06/1000 REC (exclude <5-year tenure, restrict <1280 REC) versus *a priori* results of untransformed HR=1.03/1000 REC and log transformed HR = 1.07 (no exposure restriction or tenure exclusions) Tables 4 and S7 in Attfield et al. (2012).

Results from lagged and unlagged HRs without the exclusion of workers and the deletion of high exposures show little evidence of E-R trends with any of the E-R models, whether expanded, categorical or continuous (Figures 13 and 14 and Table 6). Figure 15 compares primary findings from *a posteriori* findings versus *a priori* findings using as a referent the expanded categorical model with 15-year lags and no exclusion by tenure.

**Table:** 

*A posteriori*	Association?	*A priori*	Association?
15-year lags, Exclude <5 year tenure, restrict REC ≤1280 μg/m^3^-years, untransformed HR = 4.06(2.11–7.8)/10^3^ REC	Yes, *p* < 0.001	15-year lags, No tenure exclusion, No exposure restriction, untransformed HR = 1.03 (0.83–4.3)/10^3^ REC, log transform HR = 1.07 (0.97–1.19)	No, *p* = 0.82, *p* = 0.17

Figure 15 displays the sharp contrast between the *a posteriori* analyses that restrict the exposure range to <1280 µg/m^3^ years and exclude workers <5-year tenure, compared to the *a priori* analyses that use the full exposure range with exclusions by tenure. The *a priori* evidence does not support the lung cancer-DE hypothesis. Similar results were observed in the unlagged analyses.

### 5.3 Strengths

This is among the most important cohorts for investigating lung cancer and cumulative exposure to diesel exhaust for several reasons:

(i) It has an adequate number of cases and self-reported sufficient power to detect a twofold increased risk at the highest exposure levels.
(ii) Quantitative estimates of DE exposure are derivable from past and recent IH samples and did not have to rely exclusively on surrogates such as job or tenure.
(iii) DE exposures among UG miners are considerably higher and have a wider range than other studies and most workplaces. For example, Pronk et al. reported average REC levels in UG non-metal mines of 148 and 202 µg/m^3^ (Pronk et al., 2009); average UG exposure was estimated as 128 µg/m^3^ in this study, with a high of 216 µg/m^3^ in one of the potash mines.
(iv) Latency, or time since first exposure, is adequate (>20 years) to assess associations of lung cancer risk and exposure to DE. Most previous studies had a substantial proportion of workers with <20 years latency and/or few diesels in the workplace, which reduced the biological plausibility of a causal association. Exposures in this study are in part extrapolated from estimates of diesel HP and ventilation rates in the mines so initial exposures to DE can be estimated and latency determined.
(v) There was extensive information on potential workplace confounders, and when potential confounders were present (e.g., radon, silica, asbestos) exposures were low.
(vi) There were eight different locations in different geographic locations in the US and four different commodities that were mined (limestone, three potash, salt, three trona), none of which are suspected of increasing risk of lung cancer.
(vii) Results were said to be robust and consistent “across multiple analyses using alternative exposure estimates and modeling approaches.”
(viii) Results are not subject to healthy worker selection bias arising from workers leaving work because of respiratory irritation from workplace exposures.

### 5.4 Limitations

This study had limitations typical of cohort mortality studies. These are noted by the authors and listed here. We will then discuss in some detail what we consider to be the more serious hidden uncertainties relating to problems of *a posteriori* (not Bayesian) analyses, statistical significance and excessive comparisons, and high HRs and low exposures in surface workers versus lower HRs and much higher exposures in UG workers. We will also discuss how restricting results to *a priori* analyses significantly changes the interpretation and results.

(i) Uncertainties in exposure assessments are always a concern in epidemiology studies, especially for retrospective exposures. This issue for this study has been in part addressed by others (Borak et al., 2011) and will be discussed further in the case-control review.
(ii) There is no information on smoking, and complete work histories with information on potentially hazardous workplace exposures are lacking. These limitations are rectified in the nested case-control study (Silverman et al., 2012).
(iii) We suggest that there are limitations in the data analyses, some of which have been discussed previously. Based on data largely provided in their supplementary tables, we present study results that are consistent with the guidelines laid out in the study protocol, and thus better reflect the primary results on which we base our interpretation and conclusion.

The following seven sections are the major discussion points of the limitations in the cohort study.

#### 5.4.1 Incomplete reporting of data on complete cohort

Incomplete reporting of data on the complete cohort, so the UG worker data are used as a surrogate. Figures 13–15 display the weight of evidence regarding cumulative exposure based on analyses described in the protocol. The evidence is considered incomplete since regression data on the complete cohort are not provided, so UG workers are used as a surrogate. This lack of data on the complete cohort is considered a limitation because:

(i) Analysis of the complete cohort was indicated in the protocol and was the primary focus in the case-control study.
(ii) Adjustments for worker location produce similar E-R patterns of UG workers and the complete cohort. This comparison suggests that analysis of the complete cohort would have been appropriate.
(iii) Power is reduced as there are 78 fewer workers at the lower exposure levels <160 µg/m^3^ years; and
(iv) The complete cohort includes surface workers with low exposures who were envisioned as referents as demonstrated in the nested case-control analysis (Silverman et al., 2012). In the expanded category analysis of UG workers there are eight referents, while for the complete cohort there are 52 referents (Tables 4 and 6). Thus, restricting the analysis to UG workers results in a very small low exposed referent group.

#### 5.4.2 Restriction of exposures

Whether E-R trends exist at higher exposures is entirely dependent on the authors’ restriction of exposures to <1280 µg/m^3^ years. The lack of statistical significance for E-R trends over the entire exposure range led to inappropriate additional analyses.

The elevated HRs at >80 µg/m^3^ years could be interpreted as a plateau (apart from the peak HR in the penultimate exposure category) as indicated by the authors. Because of this plateau “we undertook analyses omitting the highest exposures to provide risk estimates pertinent to the lower range.” A log transformed regression model was fitted for the complete range of REC cumulative exposure (presumably to resolve the plateau), but this model “fitted the data less well than the restricted exposure model” and was “not statistically significant.”

These results led the authors to rely on the restricted exposure model and an incomplete reporting of regression model results for the whole exposure range. Strong E-R trends were produced by excluding exposures >1280 µg/m^3^ years, and always produced highly significant results (see Tables 6 and 7; Figures 11 and 15).

**Table 7:** Summary of increases in E-R slopes that always occur when deleting the high exposure category (>1280 µg/m^3^ years) (From Table S7 in Attfield et al., 2012).

Characteristics of UG cohort	Change in slope (HR per 1000 µg/m^3^ years) from entire exposure range to exposures <1280 µg/m^3^ years
No lag, 0 tenure exclusions	HR = 1.01 (*p* = 0.89) to 2.37 (*p* < 0.004)
No lag, <2 year tenure excluded	HR = 1.02 (*p* = 0.71) to 2.98 (*p* < 0.001)
No lag, <5 year tenure excluded	HR = 1.05 (*p* = 0.47) to 4.07 (*p* < 0.001)
No lag, <10 year tenure excluded	HR = 1.07 (*p* = 0.39) to 4.90 (*p* < 0.001)
15 year lag, 0 tenure exclusions	HR = 1.03 (*p* = 0.82) to 2.79 (*p* < 0.001)
15 year lag, <2 year tenure excluded	HR = 1.04 (*p* = 0.72) to 3.19 (*p* < 0.001)
15 year lag, <5 year tenure excluded	HR = 1.07 (*p* = 0.59) to 4.06 (*p* < 0.001)
15 year lag, <10 year tenure excluded	HR = 1.10 (*p* = 0.49) to 5.19 (*p* < 0.001)

The authors commented that HRs in this study “declined or reached a plateau” above 1280 µg/m^3^ years, which has been observed elsewhere and may be due to “misclassification at high exposures, worker selection effects, and enzyme saturation.” No evidence is provided that any of these suggested justifications are applicable to this cohort. The authors do indicate that the healthy worker effect should not occur, which is consistent with the absence of worker selection effects.

No convincing scientific reason is provided for restricting E-R analyses to exposures <1280 µg/m^3^ years. The primary basis given for this restriction was to achieve better fitting models and statistically significant E-R trends. These rationales are scientifically inappropriate. Simply stated, evidence from analyses using arbitrarily deleted exposures is an *a posteriori* analysis and should not be used for interpreting results of this study.

#### 5.4.3 Changes in HR based on tenure

“HRs were generally greater after exclusion of workers with shorter tenure” but it “was not necessary to restrict the analyses on tenure for a statistically significant exposure-response finding to arise.”

The examples given for finding statistically significant E-R trends without exclusion of short-term workers included:

(i) lagged and unlagged HR restricted exposures to <1280 µg/m^3^ years (*p* = <0.001 and 0.004) (Note that the exclusion of <5-years tenure changed *p* values to <0.001);
(ii) a log transformed HR trend (*p* = 0.046) over the full exposure range had a lower confidence interval that included 1.0.

None of the other models without exclusion by tenure or deleting high exposures were statistically significant. This is critically important inasmuch as excluding workers with <5-years tenure (or any years tenure) is an *a posteriori* analysis that should not be used for interpreting the results of this study.

A more appropriate methodology is the exploratory analyses that were conducted using an REC x tenure interaction among UG workers. Results were similar to exclusion, but the data were not shown so the reader cannot make an evaluation. Workers with longer tenure had “lower absolute risk but greater REC exposure-response slopes compared with short-term workers.”

#### 5.4.4 Emphasis on UG workers because of high risk among surface workers

The authors opined that high mortality in surface workers “initially obscured a positive diesel exhaust exposure-response relationship” in the complete cohort. As the authors note, this postulated obfuscation disappeared when adjustments were made for worker location. It is not clear why the analysis did not then follow the original plan to analyze the complete cohort with adjustments being made for worker location. In that regard, it is noteworthy that the complete cohort was used in the case-control study (Silverman et al., 2012).

The apparent effect from quartile analysis of the complete cohort without adjustment for worker location (Figure 8) led to an emphasis on surface workers and UG workers as separate cohorts. The implausible E-R pattern among the surface workers led to the stated conclusion of “an increasing trend in risk of lung cancer mortality with increasing DE exposure for surface workers with longer tenures” and that DE may be hazardous in open spaces.

The lung cancer SMR was higher for surface workers than for UG workers, and thus higher than expected if the lung cancer-DE hypothesis is correct. Lung cancer SMRs were 1.22 and 1.33 for UG and surface workers, respectively, and could be due to smoking. This is unlikely however as smoking prevalence among cases was similar for surface and UG workers: non-smokers 7.3 versus 8%; former smokers 29 versus 37.5%; and smokers 60.9 versus 54.5%, respectively (Silverman et al., 2012).

If DE is the cause of elevated lung cancer risk, one would expect to find the higher HR among UG workers, who on average had 75 times higher REC exposure than surface workers (128 vs. 1.7 µg/m^3^) and nearly three times higher exposure to respirable dust (1.93 vs. 0.67 µg/m^3^). There is no ready explanation for the counter-intuitive findings in this study, since, among other relevant points, smoking and workplace exposures overall do not appear to be associated with increased HRs in surface workers.

The high HR among surface workers at cumulative exposures ≥40 µg/m^3^ years resulted from only six lung cancer cases. These excess risks may be due to chance, smoking, or some other unknown cause, but are clearly inconsistent with lower HRs at much higher REC exposures among UG workers.

The E-R trend among surface workers is inconsistent and implausible. There was a statistically significant E-R trend in the untransformed regression model (*p* = 0.03) but not in the log transformed model (*p* = 0.84) and there was no trend below about 40 µg/m^3^ years in the expanded categorical model (15-year lag, exclude <5-tenure; Tables 5 and S11). Above this threshold, HRs increase two-fold in the next exposure category culminating in a 8.7-fold (1.61–46.9) increased HR point estimate for the highest exposure category of 80–160 µg/m^3^ years. This estimate is almost two times greater than the highest estimate of 5.01 (1.97–12.8) at 640–1280 µg/m^3^ years for UG workers at 1/8 the exposure level. The most significant result was with the untransformed model with 15-year lag and excluding workers with <10-years tenure (Table S8) (*p*_trend_ = 0.01).

#### 5.4.5 Results were said to be “robust to variations in methodological approach”

The authors reported that results were robust and consistently positive for categorical and continuous regression models. For UG workers, the results for all untransformed models with restricted exposure range were significant, and nearly all log transformed models were significant for 15-year lagged and unlagged REC with and without exclusions for tenure. However, none of the untransformed models were significant for UG workers (Table 7).

More significantly, the *a priori* analyses showed positive but generally non-significant E-R trends, while *a posteriori* results from restricting the exposure range and minimum years of working experience were highly significant with implausibly high HR at the maximum exposure (Table 6).

#### 5.4.6 Statistical significance is misleading

Over 400 comparisons were made in this study, and the reference p-value should therefore be less than the conventional 0.05. It is now technically easy to perform a large number of sophisticated statistical model fittings across a large number of models and/or variables to identify associations of potential scientific interest from a single set of data. Even with a single compound and a single response, it has become standard practice to consider a potentially large number of models in an effort to adjust for differences among the exposed and the unexposed (Peng et al., 2006). From this large set of results, the researcher will usually choose a single model, or maybe a few models, based on some goodness of fit statistic and declare that model to be the true description of the exposure-response. This phenomenon has been described with several names including: model-shopping, cherry-picking, *a posteriori* choice, post-hoc selection, and “eligendi cerasus.” We will use *a posteriori* choice with the understanding that it is not related to Bayesian analysis terms.

The *a posteriori* choice is useful when investigating a previously unexplored relationship. The process will help identify relationships that exist in the data set under investigation, but will not provide information on the generalizability of the relationship to other data sets, especially if the criterion for model selection is the significance of model statistics, and cannot be used to infer causality. An underlying assumption about the significance level is that the estimate is developed from a model that was specified before the statistical analyses were performed. The reader can imagine that within 20 sets of two random numbers it is likely at least one set will have a statistically significant correlation at *p* < 0.05.

When describing the relationship in a data set that will be used for policy, the relationship needs to be validated to show it is not a random or chance occurrence. These relationships have to be statistically tested on a pre-specified model, not on subsequent models developed to generate a relationship. The significance level from the aforementioned analysis of pre-specified models is the one that should be used to assess the efficacy of the modeled relationship.

In practice, models often are modified in ways that violate the basic assumption of a completely pre-specified model in order to maximize model efficacy (or maximize the ability to produce a desired result). These violations include such acts as choosing different forms of background effects, selecting various combinations of confounders, selecting alternate exposure metrics, or choosing different lags for exposure variables. Such *a posteriori* choices may produce a spuriously inflated significance level or narrowed confidence interval that often overstates the significance of the predictors unless there is some adjustment. Hodges (1987) pointed out that reporting only the ‘best’ model result and essentially ignoring the uncertainties associated with model assumptions may lead to overconfident predictions and policy decisions that are riskier and more uncertain than one might otherwise suspect. The degree of overstating is related to the number of models tested. Chatfield commented, “It is indeed strange that we [statisticians] often admit model uncertainty by searching for the best model but then ignore this uncertainty by making inferences and predictions as if certain that the best fitting model is actually true” (Chatfield (1995).

Bayesian model averaging (BMA) is one method that can help eliminate the concern of multiple models and provide more realistic estimates of the uncertainty of relative risks (Clyde 2000). BMA works on the principal that it is possible to calculate the Bayes probability that a model is the correct model for a given data set. After considering all possible models one can estimate a common parameter of interest and its standard deviation and take into account multiple testing. A cruder method to deal with problems of multiple model testing is to change the criteria for significance, as for example from *p* < 0.05 to *p* < 0.005. This method was suggested by the HEI Health Review Committee (2003) for revised analyses of the ACS and Six Cities cohort studies (Krewski et al., 2000).

Neither of these methods (BMA or changing the level at which significance is declared) has been universally applied, so the concern remains about minimizing the stated importance of an exposure-response model associated with multiple testing. In this regard, Hill’s advice (Hill 1965) remains sound: when interpreting for causality, do not over-emphasize statistical significance tests, as systematic error is often greater than random error. He questioned the usefulness of statistical significance in situations where differences are negligible, and specifically cautioned against methods where the “glitter of the *t* table diverts attention from the inadequacies of the fare.”

Statistical significance is considered less informative than confidence intervals, but chance needs to be a factor in interpreting study results. If the E-R patterns are not definitive, or *p* values are marginal, then the evidence from those results are given less weight than if they are highly significant. This study raised concern regarding chance and statistical significance, but this concern applies to all studies.

Primary results presented in Figures 13 and 14 for the complete cohort and UG workers fail to demonstrate conclusively that excess lung cancer is statistically associated with DE exposure. The untransformed regression models are non-significant with or without 15-year lags. The log transformed regression model is non-significant in the 15-year lagged analysis, and a marginally significant E-R trend (*p* = 0.045) appears in the unlagged analysis. The strongest HRs are observed using the untransformed model, which at high exposures (4000 µg/m^3^ years) produce HRs of 62 (28–165) (*p* = 0.12) in the 15-year lagged analysis and 57 (35–96) (*p* = 0.89) in the unlagged analysis. The 15-year lagged *a posteriori* untransformed HR was 181 (15–21,676) (*p* < 0.001) at 1280 µg/m^3^ years when exposure range was restricted to <1280 REC and workers <5 years tenure were excluded (Table 6).

It should be noted that using log transformed power models to “accommodate leveling-off at highest exposure levels” might be useful for getting a better fitting model with smaller p-values, but there is no plausible biological reason why the dose-response would level off if DE were a real causative agent or why HRs would remain below 1.0 below 1000 REC. Moreover, both untransformed and log transformed regression models produce implausibly elevated HRs that bear little or no resemblance to the categorical data or to biological reality. For example, the regression models with restricted exposure range estimate that HRs increase 180-fold at 1280 µg/m^3^ years. Untransformed and log transformed regression with 15-years lag and no tenure exclusions produce 62-fold and 10-fold increased HRs at the highest exposure of about 4000 µg/ m^3^ years (Table 6). But even with these very high HRs, the models are not statistically significant.

### 5.5 Summary

In summary, the weight of evidence regarding E-R from the UG and complete cohorts indicate elevated HRs at higher cumulative exposures, but the evidence shows no clear relationship regarding an E-R trend based on the *a priori* guidelines for analysis.

The expanded categorical analysis is more like regression models and is preferable to the quartile models. The initial quartile analysis was of the complete cohort, but without adjustment for worker location (surface vs. UG) there was no association of lung cancer and DE. The lack of an E-R association in the total cohort was thought to be caused by the surface worker cohort somehow obscuring the association. It was only subsequent and separate analyses that split the cohort by work location that led to the conclusion of “an increased risk of lung cancer in both underground and surface workers.”

Analysis of the UG cohort with restricted exposure range and exclusion of short-term workers with <5-years exposure produced E-R trends that led the editors to conclude “DE may be hazardous in both confined and open spaces and may represent a public health as well as industrial hazard.”

The authors’ conclusion of support for the lung cancer – DE hypothesis for UG workers was based on secondary *a posteriori* analyses that are very problematic in the scientific sense and produced misleading and inaccurate conclusions based on biased data. These unplanned analyses produced significant E-R trends in UG workers based primarily on 15-year lags, excluding workers with <5-years exposures, and fitted models with exclusion of high cumulative exposures. The regression models produced three-fold greater HRs at the highest restricted exposure level than the expanded category model. Conclusions should not be based on restricted models, and the resultant regression models that were used have produced implausible results.

*A priori* data reported in their Supplementary tables do not support the lung cancer-DE hypothesis. This conclusion is derived from lagged and unlagged regression models of the UG cohort without the *a posteriori* deletion of high exposures or the exclusion of short-term workers.

## 6. NcI/NIOSH nested case-control study of non-metal miners: Silverman et al. (2012)

### 6.1 Description

This nested case-control study had 198 lung cancer cases and 582 controls matched by facility, gender, race/ethnicity and birth year. Eligible workers must have worked at least 1 year after introduction of diesel engines into the mine (1947–1967) until the end of follow-up, December, 31, 1997. Telephone interviews were conducted with controls and next-of-kin for information on demographics, smoking history, occupational history, medical history and usual diet. The CO-surrogate-based estimates of REC exposures were the same as in the mortality study.

Quartile and tertile cutpoints were based on a similar number of cases in each category, and metrics included cumulative exposure (µg/m^3^ years), average intensity (µg/m^3^), and years exposed. Conditional logistic regression models included terms for potential confounders including smoking × location, smoking (packs/day), high risk job for lung cancer of >10 years, and history of NMRD (i.e., pneumoconiosis, emphysema, COPD, silicosis, or TB) diagnosed more than 5-years before death. Continuous models included log-linear, power, linear, and linear-exponential models. The optimal lag period was 13–17 years for average intensity and 15-years for cumulative exposure; however results for both zero lags and 15-year lags were presented.

Methods were simplified compared to the cohort analysis (Attfield et al., 2012) in that no additional workers were excluded beyond the <1 year tenure criterion for inclusion in the cohort. Results for the restricted exposure range (i.e., restricted to exposure levels less than 1280 µg/m^3^ years) were reported, but apparently not used for the conclusion.

### 6.2 Results

**Figure 16:**
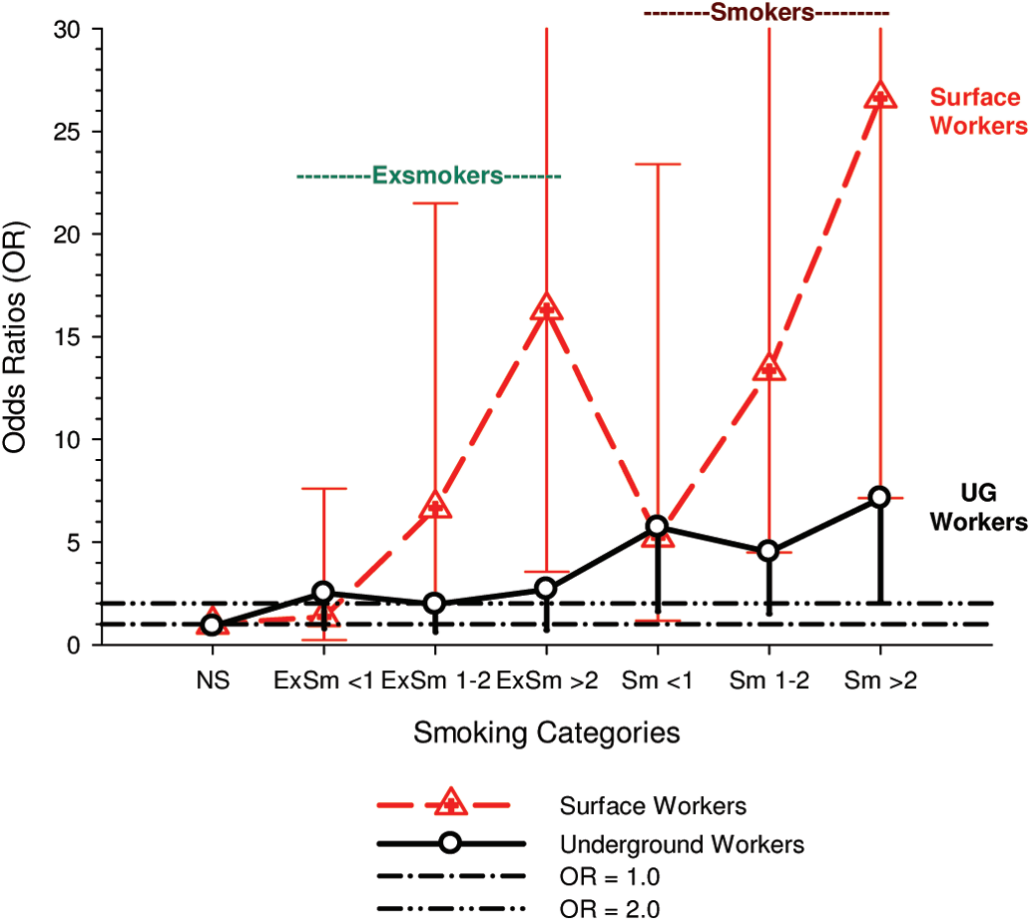
Lung cancer odds ratios for smoking status/smoking intensity for surface workers (REC = 0–8 μg/m^3^) versus UG workers (REC = 1–423 μg/m^3^) in case-control study (Silverman et al., 2012, Table 2).

Smoking was associated with increased risk of lung cancer, but the effects were dissimilar by work location, with surface workers showing a stronger association than UG workers (Figure 16). Adjustment for smoking intensity/workplace interactions were made in the E-R models as well as adjustments for history of respiratory disease and ≥10-years in a high risk job for lung cancer.

#### 6.2.1 Underground workers

Quartile unlagged and 15-year lagged analyses of cumulative REC exposure showed positive trends with no tendency to level off at higher exposures. The 15-year lagged E-R was significant (*p*_trend_ = 0.004) and 1.5 to 2.5fold greater than the unlagged trend, which was not significant (*p*_trend_ = 0.12). The E-R among the cohort of UG workers was intermediate with a tendency to level off in quartiles three and four in the 15-year lagged analysis.

**Figure 17:**
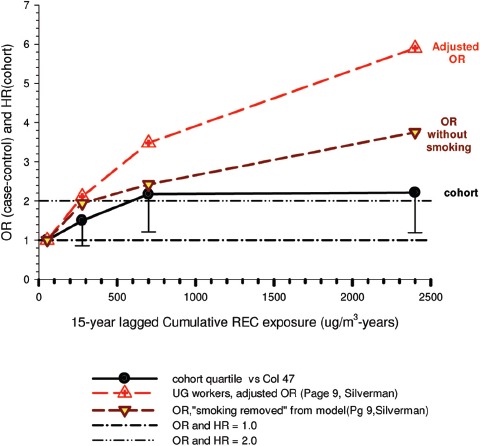
HRs and ORs of lung cancer and 15-year lagged cumulative REC among UG workers from cohort (Table 4 in Attfield et al., 2012), adjusted ORs with smoking removed from model (ORs at cohort exposure cutpoints) (page 9 from Silverman et al., 2012).

The higher ORs in the case-control study “may be partly due to negative confounding from cigarette smoking because current smoking was inversely related to diesel exposure in underground workers, namely “36 and 21% for current smokers in lowest vs. highest cumulative REC tertile, respectively.” This inverse relationship between DE exposure and smoking is shown when smoking adjustments are removed from the quartile 15-year lagged model, thereby reducing the smoking adjusted ORs and making the slope intermediate between the smoking adjusted ORs and HRs from the cohort (Figure 17).

#### 6.2.2 Surface workers

**Figure 18:**
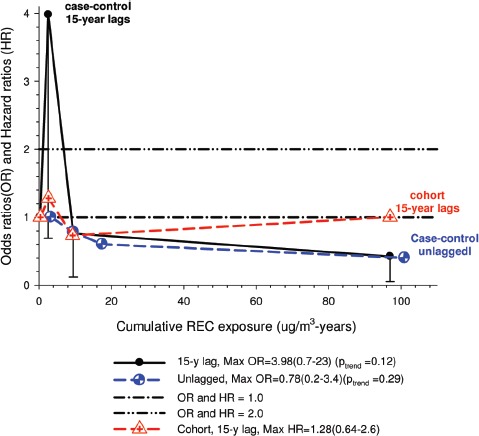
Relative risks of lung cancer with cumulative REC exposure among surface workers with and without 15-year lags in cohort study (15-year lags, Table 5 in Attfield et al., 2012) and casecontrol study (unlagged and 15-year lags) Table 5 in Silverman et al. (2012).

There were no E-R trends among surface workers in the quartile models in both the case-control and cohort studies. There is a four-fold increased OR in the 2nd quartile with 15-year lags. The reason for this elevated OR is unclear as it is not reflected in the unlagged analysis and does not appear to be related to smoking. All other ORs are <1.0 showing nonsignificant negative E-R trends. In the cohort study there are no trends in the 15-year lagged quartile analyses, although there was a positive slope for the 4th quartile (Figure 18), which in the expanded categories with <5-year tenure showed two-fold increased HRs, but with few workers.

All subsequent analyses include surface workers as the referent group in the combined group of all cases and controls.

#### 6.2.3 All workers

**Figure 19:**
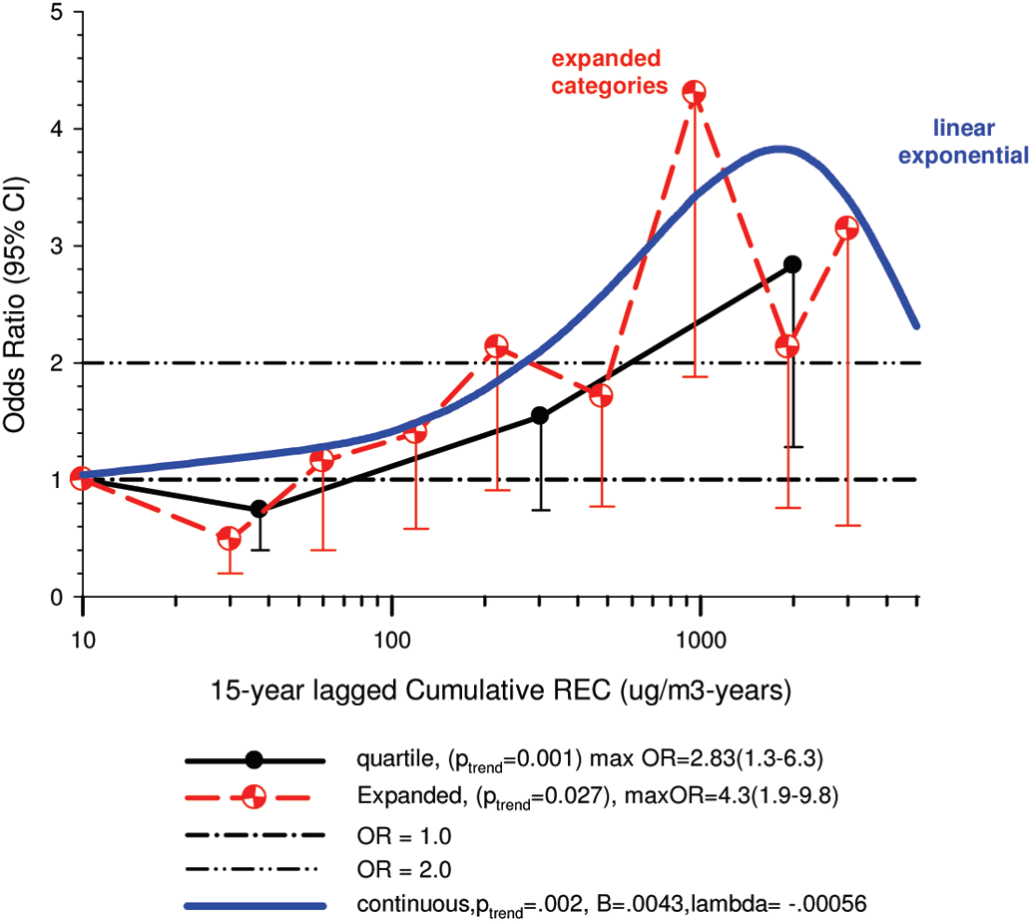
Lung cancer odds ratios for 15-year lagged cumulative REC for quartile, expanded categorical with split highest exposure category and continuous linear-exponential regression models for entire study population in case-control study (Tables 3, S1 in Silverman et al., 2012).

The categorical analyses for all workers show clear positive and significant slopes. The quartile 15-year lagged model showed the strongest association (*p* = 0.001) of all E-R trends; when the 4th quartile was split at the median of 1000 μg/m^3^ years the *p*_trend_ became 0.002 (Figure 19). Note that the *p*_trend_ is the significance level associated with the test for trend in the exposure groups. The best fitting continuous model was the linear-exponential model which showed a significant positive slope (β = 0.0043, λ = −0.00056, *p*_trend_ = 0.002) with a “leveling off of risk for exposures above 1000 μg/m^3^ years” and a subsequent decline in risk as exposure increased further. It is unclear why the continuous model begins to decline at higher cumulative exposures when the categorical models indicate a rise in risk (Figure 19).

**Figure 20:**
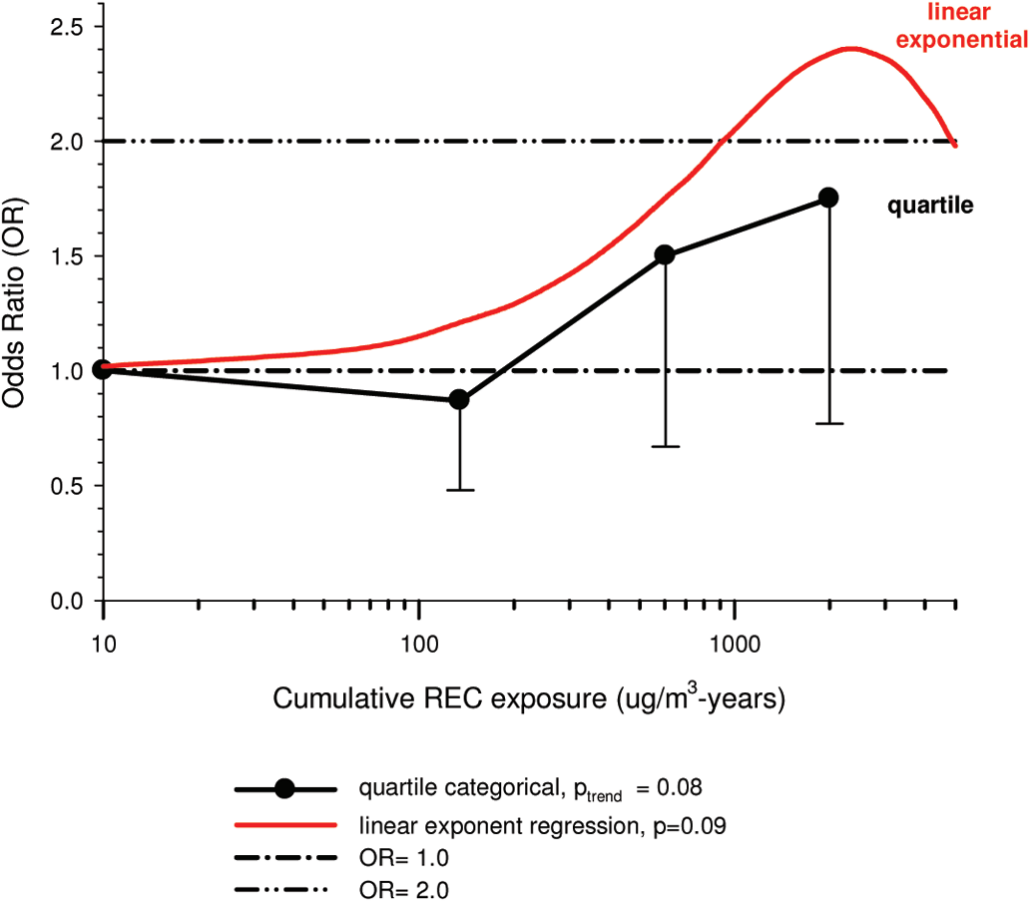
Odds ratios (ORs) and 95% confidence intervals for unlagged cumulative REC exposure and lung cancer in quartile and linear-exponential (β = 0.0016, λ = −0.00042) regression models in DEMS nested case-control Tables 3 and S3 in Silverman et al. (2012).

In the unlagged models the slopes are positive but not statistically significant. In the quartile categorical analysis the *p*_trend_ is 0.08 with elevation of ORs in the 3rd and 4th quartiles only. The linear-exponential regression was the best fitting model with *p*_trend_ = 0.09. The unlagged model also shows the peak of elevated ORs to occur near 2000 μg/m^3^ years, with a sharp decline at higher cumulative REC exposure levels (Figure 20). The authors unconvincingly attributed this decline to “exposure misclassification because recent exposures may not have had sufficient time to contribute to lung cancer risk.”

**Figure 21:**
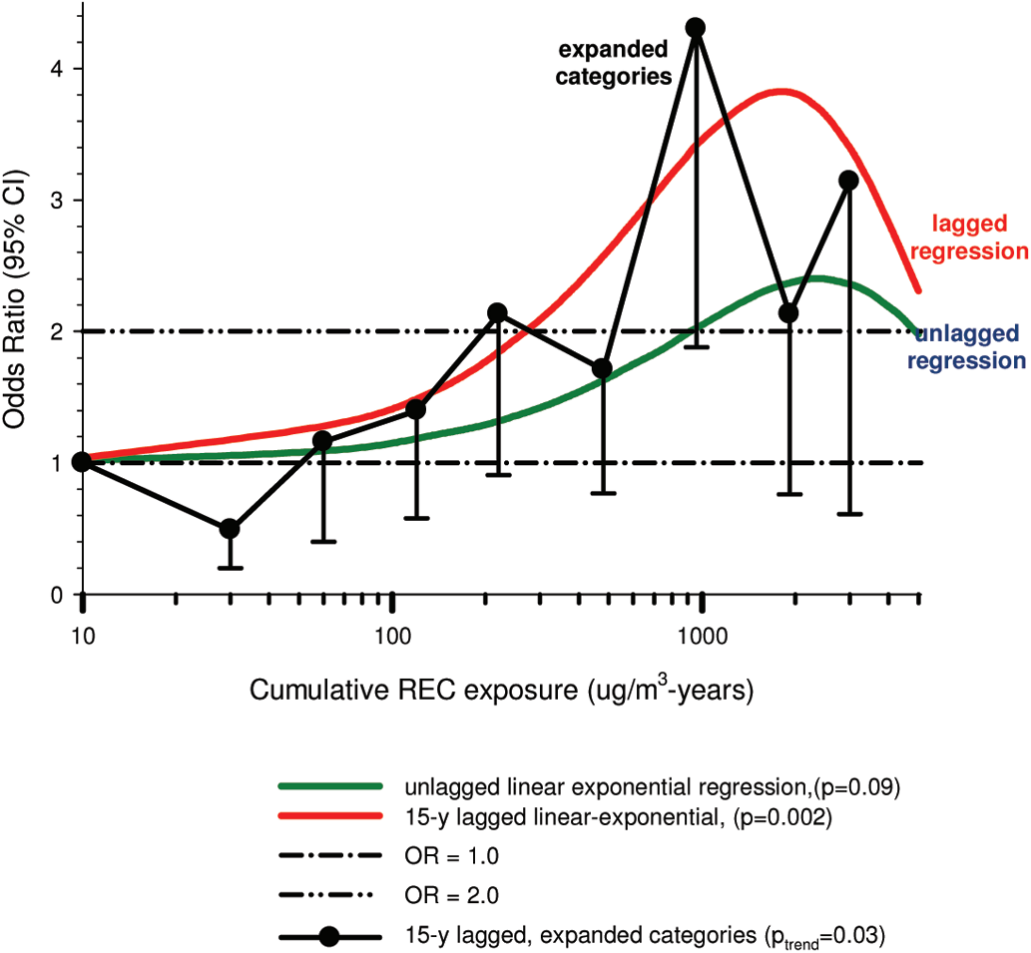
Odds ratios for lung cancer and cumulative REC in expanded category and linear-exponential (β = 0.0043, λ= −0.00056) models lagged 15-years and unlagged linear-exponential (β = 0.0016, λ = −0.00042) regression models from Tables S1, S2 and S3 in Silverman et al. (2012)

The best fitting continuous models from the case-control study are linear-exponential, with *p*_trend_ values of 0.09 and 0.002 for the unlagged and 15-year lagged models respectively. It is not clear why the continuous model ORs continue to decline despite the elevated OR in the last exposure category (Figure 21). The noteworthy feature in Figure 21 is the shallower and non-significant slope of the unlagged regression model. The peak is shifted to the right perhaps 500 μg/m^3^ years. Shifting from unlagged to 15-year lagged exposures reduces cumulative exposure so the referent quartile is reduced from <19 μg/m^3^ years unlagged to <l3 μg/m^3^ years in the 15-year lagged analysis. No expanded categorical model was provided for the unlagged analysis.

**Figure 22:**
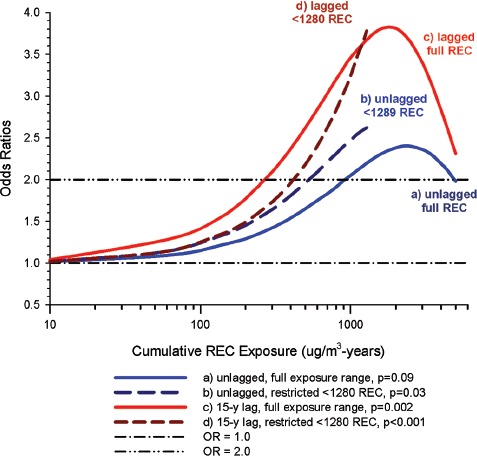
Lung cancer and cumulative REC with linearexponential models unlagged with (a) full REC (β = 0.0016, λ = −0.00042) and (b) REC <1280 μg/m^3^-year (β = 0.0025, λ = −0.00011) versus 15-year lagged models with (c) full REC (β = 0.0043, λ = −0.00056) and (d) REC <1280 μg/m^3^-year (β = 0.0025, λ = +0.00053) from Tables S1 and S3 in Silverman et al. (2012).

Figure 22 shows the marked changes in E-R patterns when the exposure range is restricted to <1280 μg/m^3^ years. The unlagged linear-exponential model over the full exposure range is non-significant *p* = 0.09), while the 15-year lagged model has a steeper slope with peak OR increased nearly four-fold (*p* = 0.002). Both restricted models produce statistically significant E-R slopes. As noted in the discussion of the cohort analysis, analyses restricting the exposure range to <1280 μg/m^3^ years are considered *a posteriori* analyses and should not be considered when interpreting results. It is unclear why these data are presented in the case-control study as they were not used by the authors in their conclusions. They used the 15-year lagged analyses shown in Figures 21 and 22.

The authors conclude “Our findings provide further evidence that diesel exhaust exposure may cause lung cancer in humans and may represent a potential public health burden.” The pattern in the E-R trend was “a steep increase in risk with increasing exposure at low-to-moderate levels followed by a plateauing or perhaps a decline in risk among heavily exposed subjects.” We feel that the authors’ conclusion is based primarily on results from the 15-year lagged analyses displayed in Figures 19, 21 and 22.

### 6.3 Strengths

**Figure 23:**
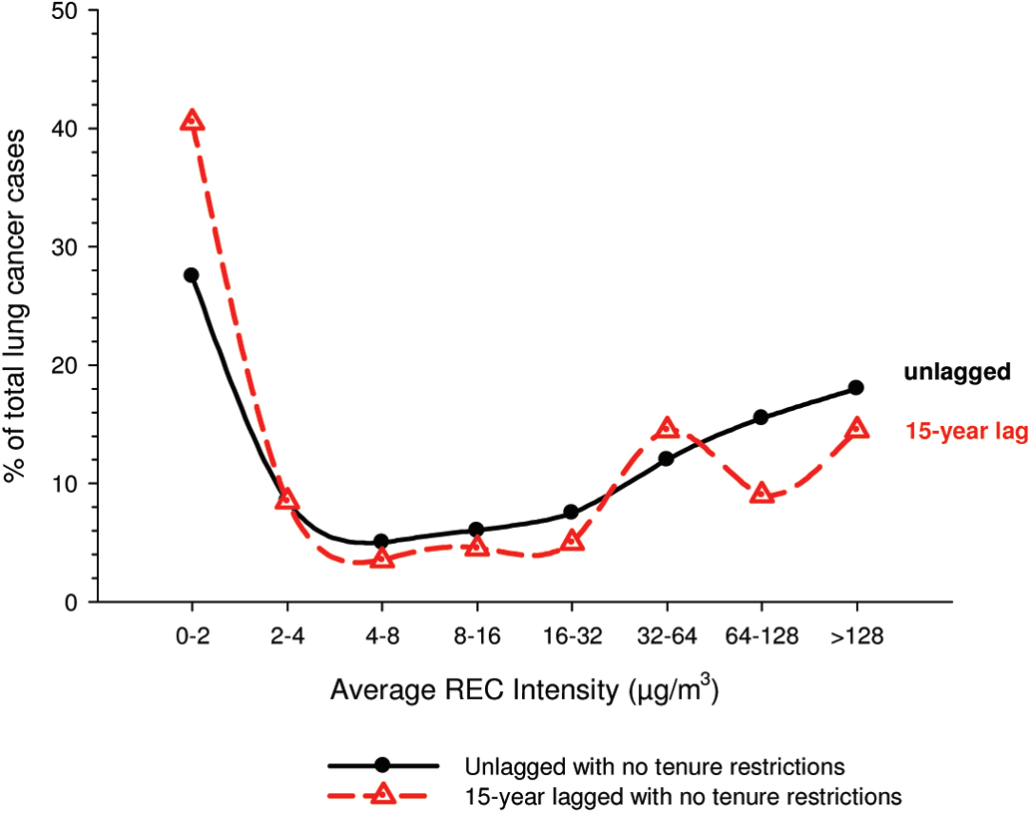
Distribution of 200 lung cancer cases in complete cohort by 15-year lagged and unlagged average REC intensity (µg/m^3^) with no tenure restrictions (Tables S5 and S6 from Attfield et al., 2012).
- Rate of participation in interviews for work and smoking history was unusually high for both cases (98%) and controls (94%).
- Completed questionnaires on work and smoking histories allowed adjustments for potential confounding from smoking and other lung cancer risk factors. Adjustments for potential confounding from workplace exposures (e.g., silica, radon, asbestos) could also be made although exposures were low. Salt, trona, and potash products of mining activities pose no apparent lung cancer risk.

Major strengths of this study include:

- The large study size allows detection of statistically significant exposure-response relationships in the 15-year lagged models.
- There is an adequate latency period for the development of lung cancer that is potentially attributable to the exposure of interest. Cumulative exposure is not estimated until diesel engines are introduced into the mine. An adequate latency period makes this study unique as no other study can make this claim.
- Detailed work histories and surrogate-based quantitative reconstructions of DE exposure are based at least in part on extrapolations from recent sampling results. The range of DE exposure was quite wide from low to negligible among surface workers to quite high among UG workers. Pronk et al. (2009) reported that highest exposures to EC occur in UG mining, where the range of reported EC levels was 27–658 µg/m^3^. Historical REC estimates for this cohort were estimated to be around 600 µg/m^3^ (Vermeulen et al., 2010a,b). Surveys conducted during 1998–2001 found average REC exposures were 2–6 µg/m^3^ among surface workers, 31–58 µg/m^3^ for UG workers at the mine with the lowest average exposures, and 313–488 µg/m^3^ at the mine with the highest average REC air concentrations (Coble et al., 2010). The distribution by exposure among lung cancer cases in the complete cohort with no tenure restrictions shows 28% of cases have no exposure (=referents) in the unlagged cohort, and 43% with 15-year lags by average intensity; the distribution is reversed to 18 and 7%, respectively, in the highest exposure category of ≥128 µg/m^3^ (Figure 23).

### 6.4 Limitations

#### 6.4.1 Smoking and other potential confounders

Three categories of potential confounding risk factors are adjusted for in the case-control analysis. These are smoking x worker location interaction (16 categories), history of respiratory disease (four categories), and employment in other high risk occupations (five categories).

Smoking is of particular concern because of the strong associations (higher ORs) and because of the marked difference in risk between surface and UG workers. The risks of lung cancer among surface workers show typical E-R patterns with ORs rising steeply with increased cigarettes/day among both ex-smokers and current smokers. This expected pattern is not observed among UG workers as light and heavy smokers have about the same ORs, and this is true for light and heavy ex-smokers as well (Figure 16). A possible explanation for this is that smoking information was subject to misclassification (the smoking information for cases was mainly from next-ofkin) and potentially resulting in imperfect adjustment and residual cofounding by smoking. The risks of smoking are considerably greater overall with >90% of cases having 3 to 12-fold increased ORs (their Table 2) (Figure 16).

In addressing the issue of confounding we remind the reader that two criteria must be met for a variable to be a confounder. These are (i) it must be a risk factor, and (ii) it must be associated with exposure.

The first criterion is clearly met in this study, as potential confounders (e.g., smoking, history of respirable disease, employment in high risk jobs) are risk factors as shown in their Tables 1 and 2.

The second criterion is only partially met. Smoking is associated with exposure among UG workers as shown by the lower prevalence of smoking among higher exposed UG workers. The only other data provided on criterion 2 are found in their Table 6 which indicates little or no association of current smoking with DE exposure in all study subjects. This second finding, summarized in Table 8, indicates little or no need for adjustment for confounding from current smoking in the major analyses involving all cases and controls because the second criterion for being a confounder is not met. Therefore, there should be little or no adjustment effect for smoking. Herein lays a major limitation in the results of this study.

We will discuss the second criterion as well as the unexpected size and direction of the confounding effects reported in this study. The authors indicated that smoking is a negative confounder among UG workers, so when smoking adjustments are made the effect is to increase the crude ORs. On page 9 the authors indicate there is “negative confounding from cigarette smoking because current smoking was inversely related to diesel exposure in underground workers. That is the prevalence of smoking was 36 and 21% among lowest and highest cumulative REC tertiles respectively.” The negative confounding effect of smoking with these prevalences is calculated to be about 0.7; that is the confounded OR will be about 0.7 times the true OR if the strength of association for smoking is five (McNamee 2003), which approximates the RR among UG current smokers (Figure 16). The observed negative confounding effect of smoking among UG workers lagged 15-years is calculated as: (confounded OR) ÷ (unconfounded OR) = 3.75 ÷ 5.9 = 0.64 (Page 9). In this instance, the estimated and observed apparent effects of confounding (presumably mostly from smoking) appear similar. This negative confounding effect from current smoking is shown by “somewhat higher” adjusted ORs compared to ORs without smoking in the model. The differences between ORs without smoking in the model and HRs from the cohort study are effects from other confounders (Figure 17).

**Figure 24:**
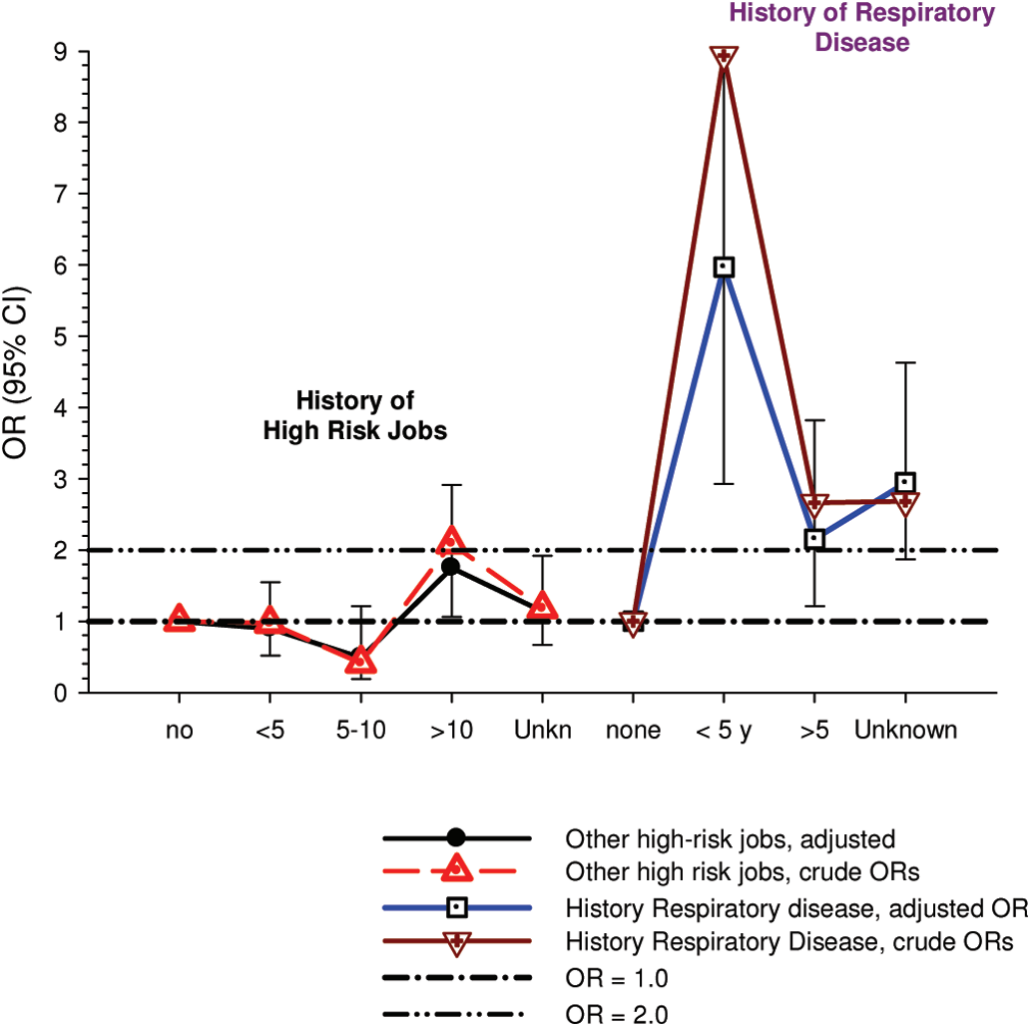
Adjusted and crude lung cancer ORs stratified by confounders from employment in other high risk occupations and history of respiratory disease (Table 1 in Silverman et al., 2012).

“Smoking” in this instance presumably includes current and former smoking. The differences between adjusted ORs and HRs shown in Figure 17 are presumably due largely to confounding from smoking × work location plus history of respiratory disease and employment in high risk jobs (Figure 24).

**Figure 25:**
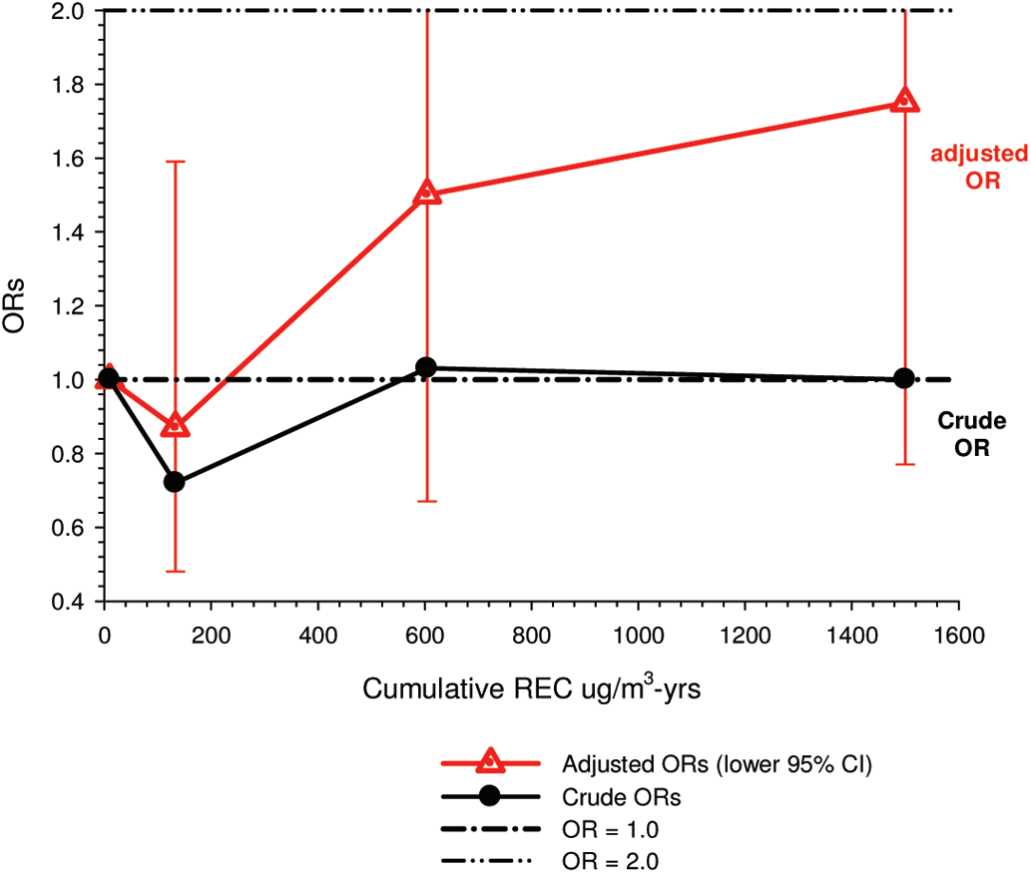
Crude and adjusted ORs for lung cancer and unlagged cumulative REC; ORs adjusted for 24 smoking × mine location, history of respiratory disease >5-years and history of high risk job for lung cancer >10-years; Crude ORs calculated from Table 3 in Silverman et al., 2012.

**Figure 26:**
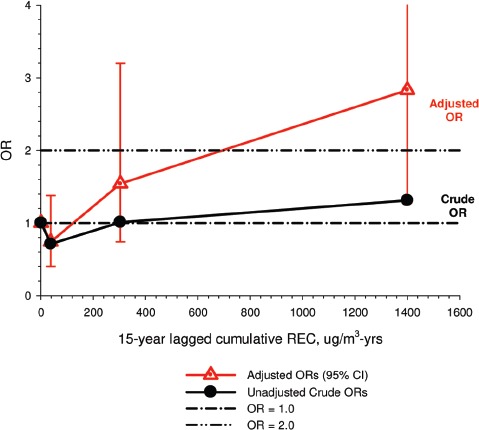
Crude and adjusted ORs for lung cancer and 15-year lagged cumulative REC; ORs adjusted for smoking × mine location interaction; >5-year history of respiratory disease and >10-year history of high risk jobs for lung cancer; crude ORs calculated from Table 3 in Silverman et al. (2012).

**Figure 27:**
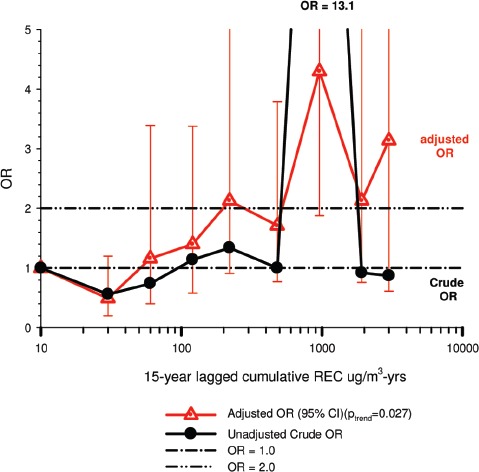
Crude and adjusted ORs for lung cancer and 15-years lagged cumulative REC for expanded exposure categories; ORs adjusted for smoking × mine work location, >5-year history of respiratory disease, and >10-year history of high risk jobs for lung cancer; crude ORs calculated from Table S2 in Silverman et al. (2012).

The confounding effects of smoking and other confounders may also be observed by comparing adjusted and crude ORs among UG workers as shown in Figures 25–27. The maximum difference occurred with 15-year lagged REC exposures where the adjusted OR is 2.8 times higher than the crude OR in the highest exposure quartile; that is the confounding factor is 0.35 (1.80 ÷ 5.10) among UG workers.

**Figure 30:**
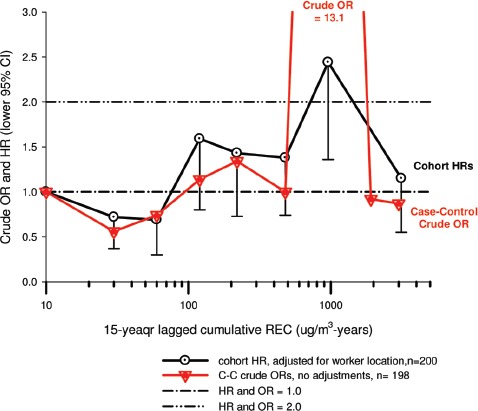
HR versus crude OR for Lung cancer and 15-year lagged REC cumulative exposure in expanded categories for complete cohort: HRs from Table S5 in Attfield et al. (2012) and crude ORs calculated from Table S2 in Silverman et al. (2012).

The differences between crude OR and adjusted ORs are also quite large among the total cohort, but in the same general range as the differences observed among UG workers. For example, the crude ORs are 46 and 28% of the adjusted ORs in the quartile and expanded categorical analyses lagged 15-years (Tables 3 and S2, Figures 26 and 30).

As a side note, effects of residual confounding from risk factors other than smoking appear comparable to the apparent effect of smoking (Figure 17). Whether they are confounders cannot be evaluated because we don't know whether they are associated with DE exposure. If they are associated with DE exposure their potential confounding effect is expected to be relatively small compared to smoking because relative risks and prevalences are less. For example, >10-years employment in a high risk job is associated with <two-fold increased OR, and all other years show no increased risk (Figure 24). With six cases in this exposure category, the potential confounding effects may be minor. Risks of respiratory disease are greater, with 9-fold and 2.5-fold increased ORs for 13–14% of cases with <5-years and >5-years of respiratory disease, respectively (Figure 24).

A critical factor in the finding of a “negative confounding effect” of smoking is that it is applicable to UG workers only; it is not applicable to the overall results. The prevalence of smoking among controls is 23, 27.9 and 25% among high, medium and low exposure tertiles among all current smokers (Table 8, their Table 6). This contrasts with prevalences of 21 and 36% among UG current smokers in the highest versus lowest cumulative REC tertiles. The similarity of smoking prevalences by DE exposure among all controls indicates negligible association between smoking and DE, and therefore small or negligible confounding among all participants. Moreover, the primary focus in the case-control study is on all participants, not just UG workers. In the complete cohort of all workers there is no apparent confounding from current smoking in the case-control study.

If true, this limitation dramatically changes the results and conclusions derived from this study. Confirmation of this hypothesis of negligible confounding from smoking requires more information and analyses from the authors and independent investigators, but we will present a rationale for our conclusion that there is negligible confounding from current smoking and the purported E-R trends associated with DE exposure are largely due to incorrect adjustments for non-existent confounding from smoking.

Since smoking is commonly shown to be a positive confounder in occupational SMR studies of lung cancer, the usual adjustments for smoking (if attempted), reduce the SMR (as workers generally smoke more than the general population and so smoking is associated with exposure). But this is a nested case-control study, so the referent group is not the general population but is comprised of workers with lower DE exposure (but not necessarily less exposure to cigarette smoke). In E-R analyses it is commonly assumed that smoking prevalence is largely independent of exposure. That is, smoking prevalence is often similar at high, medium and low exposure levels. When this occurs smoking is not a confounder, or the differences in distribution may be small so effects of confounding will also be small. But the literature also indicates that the association of risk factor and exposure has rarely if ever been considered (or at least data are rarely presented) in E-R analyses where adjustments are made for smoking.

Data shown in Table 6 from Silverman et al. were used to calculate the prevalence of smoking among controls by exposure to REC. These data indicate that current smoking cannot be a strong confounder, and at most is a very weak confounder, because current smoking is not associated with REC exposure. The prevalence of smoking among controls does not vary significantly by cumulative REC tertiles (i.e., 23 vs. 27.9 vs. 25%). Therefore there is no association of current smoking and DE exposure, and current smoking cannot be a significant confounder (Table 8).

If current smoking is not a confounder there should be little change in crude ORs when adjustments are made for smoking. There could be confounding effects from ex-smokers, history of respiratory disease and employment in other high risk jobs, as those risk factors could be associated with REC. While those data are unavailable, it seems likely that their distribution may be similar enough to the distribution of current smoking to produce relatively weak associations with DE exposure and therefore weak adjustment effects.

The evidence on the lack of association between smoking and REC in current smokers leads to the conclusion that smoking adjusted ORs in the E-R analyses are unreliable and too large. If the E-R trends are unreliable, then what is the association between lung cancer and REC exposure? If current smoking is not a confounder, then the closest approximation to the ‘true’ relationship is more likely to be the crude ORs.

Calculated crude ORs show a consistent lack of E-R trends (Figures 25–27). These are crude ORs without matching of cases and controls, so the E-R patterns are an approximation of actual trends. But this approximation is likely to be similar to the E-R pattern based on matched calculations. If the distribution of former smokers was similar to that of current smokers, one would expect E-R trends to be similar to the crude E-R trends. If former smokers and cases with other risk factors are much more prevalent at low REC exposure there will be a negative confounding effect and adjusted ORs will be larger than crude ORs. If the reverse occurs there is a positive confounding effect and adjusted ORs should be less than crude ORs. Or there may be little association of formers smokers with DE exposure and negligible confounding from former smoking and negligible adjustment effect on ORs. In this instance, the E-R pattern from crude ORs likely approximates the “true” E-R pattern.

The actual confounding effects should be confirmed as the only data on distribution of risk factors by exposure was for current smokers. The distribution of other risk factors is undoubtedly different than that of current smoking among all participants, but unless markedly different adjustments for their confounding are unlikely to produce large changes in the crude ORs. Until the associations of risk factors and REC are confirmed, E-R trends from this study are unreliable.

**Figure 28:**
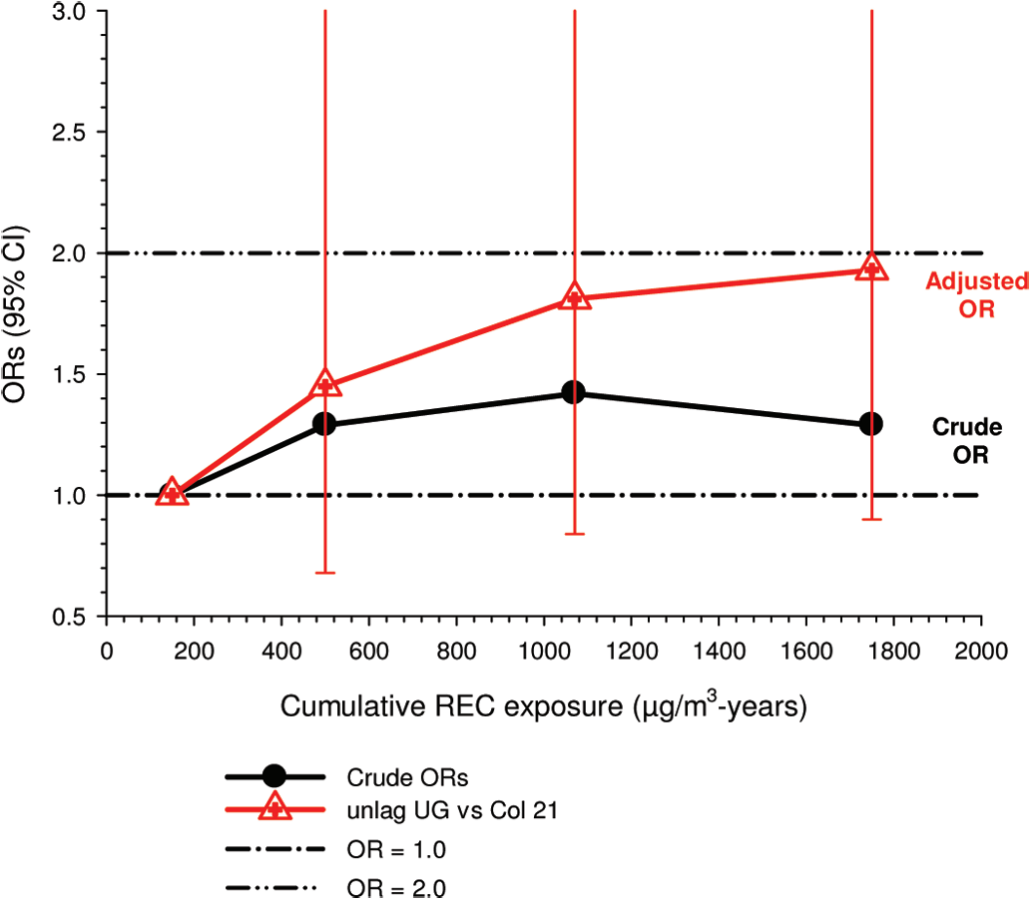
Crude and adjusted ORs for lung cancer and unlagged cumulative REC among underground (UG) workers; ORS adjusted for smoking × mine location, >5-years respiratory disease and >10 years history high risk job for lung cancer; crude ORs calculated from Table 4 in Silverman et al. (2012).

**Figure 29:**
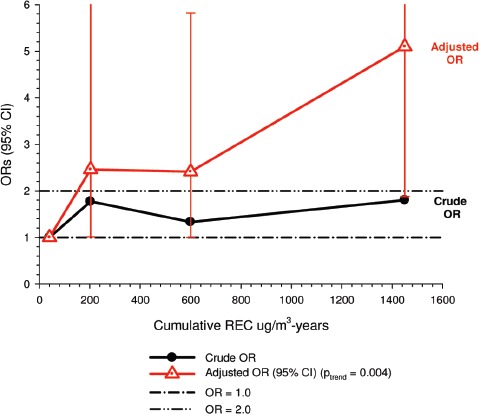
Crude and adjusted ORs for lung cancer and 15-year lagged cumulative REC among underground (UG) workers; ORs adjusted for smoking × mine work location, >5-year respiratory disease and >10-year history high risk job for lung cancer; crude ORs from Table 4 in Silverman et al. (2012).

The virtual absence of a confounding effect from current smoking and potential minor confounding effects from other variables suggests that the smoking x worker location is the primary cause of the large positive effect on the adjusted ORs. This conjecture is consistent with a similar “adjustment effect” in UG workers where there is a large and negative confounding effect between crude OR and adjusted OR (Figures 28, 29). But there should be only a small adjustment effect because in the absence of surface workers the smoking x work location adjustment effect is zero.

The smoking × worker location adjustment effect appears to be incorrect, and two possible sources of error come to mind.

- Perhaps smoking prevalences for UG workers were used instead of prevalences for the complete cohort as shown in Table 8. This possible error is suggested by the use of UG prevalences in the authors' comment regarding negative confounding.
- The statistical model may be unstable because of empty cells as suggested by the large number of adjustments and wide confidence intervals.

Our conjecture suggests that the E-R trends in the case-control study are largely due to upward-biased smoking adjustments and that residual confounding effects from other risk factors are relatively minor. If so, HRs from the complete cohort will be similar to crude unadjusted ORs from the case-control study. Figure 30 shows similar E-R patterns for HRs and crude ORs for 15-year lagged exposures. The primary differences are that HRs are adjusted for worker location, age, race and sex and the referent group are all eligible members of the cohort, while the referent group in the calculation of crude ORs is comprised of 562 randomly selected controls matched by mining facility, sex, age and race.

The implausibly large and positive adjustment effects for confounding that produced the unreliable E-R trends remains unexplained. The authors' conclusions appear to be based on E-R trends in the complete cohort produced by smoking adjustments based on confounding among UG workers. Current smoking is not associated with cumulative REC exposure among all cases and control, so among the complete cohort there should be a negligible adjustment effect for current smoking. Until the anomaly of a large and negative confounding effect is sorted out and confirmed, these case-control results should be considered inconclusive and the authors conclusions potentially unsupported by the data.

Results from the simple comparison of HR and crude OR are consistent with a result of no association of lung cancer and cumulative REC exposures in the DEMS study population. The apparent E-R trends in the case-control study may be due to incorrect adjustments for a negative confounding effect that in large part does not appear to exist.

Note: The NCI website said non-smokers at the highest level of DE exposure were seven times more likely to die from lung cancer than non-smokers in the lower exposure category (Lacey and Hegstad 2012). The only data provided in the published report compares non-smoking UG and surface workers with ORs of 0.90 (0.26–3.09) and 1.0 (referent) and REC intensities of 1–423 versus 0–8 µg/m^3^, respectively.

#### 6.4.2 Exposure misclassification

Quantitative estimates of exposure appear to be a strength of the DEMS studies and are described in detail in previous publications (Coble et al., 2010; Stewart et al., 2010; Vermeulen et al., 2010a,b; Stewart et al., 2012). However, exposure misclassification may still be an important limitation based on questionable accuracy of those estimates as summarized, analyzed and discussed in recent articles and letters to the editor (Borak et al., 2011; Clark et al., 2012; Crump and van Landingham 2012). Among the issues calling the DEMS exposure estimates into question are the following:

- CO has never been used before as a surrogate for DE exposure.
- CO colorimetric indicator tubes are imprecise and unreliable at low concentrations.
- Results from CO indicators are reasonably precise (±25% or greater) at high exposures, but are more imprecise and unreliable at concentrations <5 ppm (Borak et al., 2011). The majority of CO measurements in DEMS reports are <5 ppm, with 20–60% below the limit of detection (about 1 ppm) at the face of the mine with highest CO concentration. Nearly all CO measurements are in the range where precision is generally worse than ±35%.
- Prior to 1976 there were few CO measurements available so estimates of diesel horsepower (HP) in the mines along with mine ventilation rates were used to estimate CO concentrations. Thus, there are two uncertain extrapolations at issue: one from HP to CO, and the other from CO to REC. But there is no consistent relationship between CO and HP (or EC) (Crump and van Landingham 2012).
- Correlations of REC and CO are too low and variable for use in exposure assessment.
- Correlation of REC and CO is highly variable and low; the mean reported correlation was 0.41 ranging from 0.05 to 0.77 in the DEMS mines. Diesel oxidation catalysts (DOC) were introduced into mines in the 1970s and 1980s (Hesterberg et al., 2012). DOCs oxidize CO to CO_2_, which greatly decreases CO air concentrations and reduces CO:REC ratios (Hesterberg et al., 2012). Decreases in CO are immeasurable at low concentration. At higher REC levels CO will also be higher, and when CO levels are above the LOD the effect of DOCs will be measurable over time by measured reductions in CO levels down to the LOD. Under these circumstances the CO being measured will be less than the actual CO emitted before oxidation and will under-estimate REC levels. Unadjusted effects of this technology may produce biased underestimates of REC, with greater bias at the high end of exposure and decreasing bias as exposures decline. Adjustment for DOC may reduce this bias, but when CO levels are below the LOD, it is not clear how adjustments can be made.
- Confidence intervals for historical levels of CO indicated that more than 60% of the estimates were not statistically different from zero (Crump and van Landingham 2012).

These findings present interesting anomalies. The capability of differentiating job exposures should be greatest at higher exposures where the CO indicator tubes are most reliable. Thus, the confidence intervals should be narrower for those jobs and relatively wider as exposure decreases. If true, there would be greater exposure misclassification among lower exposure jobs than higher exposure jobs. However, greater exposure misclassification at the highest exposures was mentioned as a possible explanation for the attenuation of risks at the highest levels of cumulative exposure in both the cohort study and case-control studies. Presumably the DEMS authors are referencing an increased over-estimation of exposure at highest exposures which could reduce estimated ORs. But the authors provided no basis for any increased misclassification at higher exposures, although this is a possible basis for their rationale. Conjectures of the potential for increased under- or over-estimation of exposure and for increased misclassification at higher concentration needs verification.

The ultimate question of concern is whether the unreliability in the exposure estimates changes the E-R patterns or biases the estimated risks. A consistently biased under-estimate (or over-estimate) of exposures produces spuriously over-estimated (or under-estimated) ORs, but probably does not affect overall E-R patterns. On the other hand, a systematic bias at different exposure levels can affect E-R patterns. Limitations in exposure assessments may have larger effects on E-R patterns at higher exposure levels. If the misclassification is related to unadjusted effects of DOCs on CO, under-estimation of REC levels is a plausible outcome. Multiple factors suggest exposure misclassification is probable as discussed below.

(i) Estimated REC exposures are based on extrapolations from samples collected during 1998–2001, many years after the relevant era for estimating exposure levels and after the end of follow-up in 1997. What was being sampled was transitional DE, and the sampled levels were undoubtedly lower than the historical levels of TDE in the mines. DE emissions were progressively reduced by 99% in transitioning from TDE to NTDE, although the decreases in criteria pollutant emissions were probably proportionately greater than CO reductions. Historical REC levels were undoubtedly higher, in part because of the post-1990 diesel engine technology changes, as well as the reduced levels of sulfur in diesel fuel, which all came about due to increasingly stringent regulations applicable to off-road diesel engines (Hesterberg et al., 2012).
(ii) CO samples collected at low exposure levels are inaccurate and CO concentrations from diesel emissions were reduced with the introduction of DOCs, thereby reducing the CO:REC ratio to an unknown extent. DOCs reduce CO:REC ratios, so using CO as an indicator of REC may produce under-estimates of exposure. The proportion of CO exposures below the LOD is too high, so imputation of CO is necessary at these low exposure levels. And different methods for imputation of non-detectable CO levels produce different results (Crump et al., 2012).

If these facts produced under-estimated REC levels, the bias is expected to be more pronounced at higher exposure levels. This follows from the assumption that CO reductions via DOC at low diesel exposures reduce CO emissions to levels near the LOD, which amounts to a small decrease in absolute CO levels. At high diesel exposures, however the CO levels are higher, and the post-DOC CO levels remain above the LOD. Because CO can still be measured by CO indicator tubes at higher exposures, the absolute reduction in CO via DOC oxidation is greater than the reductions to the LOD. For example, if 50% of CO emissions are oxidized to CO_2_, the measurable air concentration is reduced by 20 ppm if the starting point is 40 ppm, but is reduced by 50 ppm if the starting point is 100 ppm CO.

A recent reanalysis indicates additional cause for concern about exposure misclassification. Although the possible direction of bias is unclear, this review suggests that there are unanswered questions regarding the reliability and accuracy of the exposure estimates used in the DEMS studies (Crump and van Landingham 2012). These authors outline the difficulties of estimating REC exposures which began with the introduction of diesel engines into the mines in 1940s to 1960s. REC estimates are based on samples collected largely during 1998–2001. Because of the lack of REC data, surrogates were used. CO indicator tube data were fairly numerous 1976–2001, but few samples were available before 1976. As a result, a second surrogate of horsepower (HP) was used to estimate CO levels before 1976, with extrapolations of HP to CO, and then CO to REC. We will list some of the major uncertainties discovered in this analysis and their attempted replication of NCI/NIOSH results.

- The NCI/NIOSH assumption of a linear relationship (exponent = 1.0) between REC and CO does not appear to be valid. That assumption is based on data (Clark et al., 1999; Yanowitz et al., 2000) which do not show a linear relationship (Yanowitz et al., 2000). NCI/NIOSH claimed the Yanowitz et al. exponents amounted to 0.58 (with upper confidence limit <1.0), Crump et al. (Crump and van Landingham 2012) reported exponents ranging from 0.39 to 0.44, and calculated an exponent of 0.30 using an improved method of dealing with CO values <LOD. Other evidence from 11 different types of diesel engines and seven different sites showed no universal relationship between CO and PM (with PM being a surrogate for REC). If there was a relationship it was unique for each engine type, and perhaps for each engine (Clark et al., 1999).
- The assumed relationship between CO and HP is also problematic. It also is based on (Yanowitz et al., 2000) which showed a non-significant slope (*p* = 0.08) and large variation (*R*^2^ = 0.01).
- HP was based on inventories of diesel engines and mine ventilation data that appears to be rarely available prior to 1976. Vermeulen et al. (2010a,b) indicated they were rarely available during the 1980s.
- The statistical model is unreliable for estimating REC from CO. The NCI/NIOSH test of their model found a median difference of 33% when the model contained a variable that used CO measurements from the 1998–2001 DEMS survey. If CO data from a 1976–1977 survey are used, the mean relative difference is −274%. This test indicates a poor model, even though the 1976–1977 data were used to develop the CO model.
- Crump et al. (Crump and van Landingham 2012) found substantial differences in REC estimates when the CO model was improved. The CO model was improved: by using collected data on the CO:REC relationship rather than assuming an implausible linear relationship; by taking statistical uncertainty into account rather than using only best estimates of parameters; and by using an improved method for imputing CO levels from samples <LOD. The net result was that the NCI/NIOSH REC values for most mines “do not lie completely within the confidence bands” estimated in the Crump et al. analyses.

The inability to replicate the NIOSH/NCI results – finding different results from the same data and unreliable correlations between HP:CO:REC – indicates that the exposure assessments may be unreliable and inadequate for estimating exposure in the cohort and case-control epidemiology studies. Until there is an independent verification of the DEMS exposure results, the DEMS E-R results should be considered unreliable and inconclusive.

#### 6.4.3 Model dependency

The veracity of the authors' conclusions depends on which models are chosen. The conclusions tend to be supported only by 15-year lagged models. Conclusions are not supported by unlagged models. Four different continuous regressions models (power, linear, linear-exponential and log-linear), and two or three different categorical models (quartile, expanded and modified expanded) were reported in the analyses. For the 15-year lagged models, the linear-exponential model visually fits the data well compared to the expanded categorical data model (their Figure 1C, Figures 19–21) and has the highest statistical significance. But the E-R pattern is implausible from a biological standpoint because of the declines in the upper half of the exposure range. The power model suggests the most biologically plausible E-R trend, but the >four-fold increased OR at low exposures also does not fit a plausible E-R pattern.

#### 6.4.4 Inconsistencies between cohort and case-control results

In the case-control study, it was suggested that the “unlagged approach led to exposure misclassification because recent exposures may not have had sufficient time to contribute to lung cancer risk.” The best fitting continuous models from the case-control study are linear-exponential, with *p*_trend_ values of 0.09 and 0.002 for the unlagged and 15-year lagged models respectively. It is not clear why the continuous model ORs continue to decline despite the elevated OR in the last exposure category (Figure 21). A noteworthy feature in Figure 21 is the shallower and non-significant slope of the unlagged regression model. The peak is shifted to the right perhaps 500 μg/m^3^ years. Shifting from unlagged to 15-year lagged exposures reduces cumulative exposure so the referent quartile is reduced from <19 μg/m^3^ years unlagged to <l3 μg/m^3^ years in the 15-year lagged analysis. No expanded categorical model was provided for the unlagged analysis.

Thus, the rationale for selecting the 15-year lagged analysis does not appear to apply as it did not hold true in the cohort study, nor in the studies the authors cite as consistent with the findings of this study (see discussion below). If DE is increasing the risk of lung cancer, it could exert a carcinogenic effect on the lung through ‘late-stage’ mechanisms such as cell proliferation from chronic inflammation. If this is a mechanism, then unlagged analyses would be preferred to account for these effects potentially occurring in the last 15 years before death.

Appropriate use of lags is a concern for both the cohort and case-control studies. Should a lagged or unlagged model be used as the primary description of the results? In the other cohort and case-control diesel studies with quantitative E-R trends, zero lags were reported as the primary result, and when lags were evaluated there was generally no substantive difference from the zero lag results (See Table 9). What makes the DEMS data set inconsistent with other studies? Why are the p-values so inconsistent across models and dependent on the lag periods?

**Table 9:** Summary of diesel-exposed workers in occupational cohorts with quantitative or semi-quantitative estimates of cumulative exposure to diesel exhaust and analysis of exposure-response trends.

	Coal miners (Johnston et al, 1997)	Truckers (Steenland et al., 1998)	Railroad engineers/conductors (Laden et al., 2006)	Potash miners (Neumeyer-Gromen et al., 2009)	Non-metal miners (Silverman et al., 2012)
Exposure units	Respirable g/m^3^ h from historical PM & NO_2_ samples	μg/m^3^ years EC based on emissions, 1990 samples = transitional DE; actual exposure = TDE	Intensity-years (emission × HP × fuel consumption) = g/mile emissions; ecological	mg/m^3^ years total carbon, 1992 samples	μg/m^3^ years REC
Latency	Potential 35 years. follow-up; but ∼50% latency short	Essentially all <20-years; inaccurate estimates of “dieselization”	Hired 1945–1949, both steam and diesel; ∼70% diesel only with adequate latency	Latency >20-years. 80–90%;	Presumably adequate
Lags/lung cancer deaths	Zero and 15-year lags/; both non-significant after adj pit/632 cases	Zero years; 994 cases 1982–1983, 1085 controls	5-years; 2396 cases among engineers/conductors, 918 unexposed (clerks, signal maintainers), 880 shopworkers	Zero years 61 cases	Zero years 198 cases, 562 controls
Exposure-response	Cox: unlagged HR = −0.0296/0.57 mg/m^3^ year diesel PM; 15-year lag HR = 0.1451	Logistic: log = 0.1797(0.0696) (*p* = 0.01); linear = 0.000352 (0.000155) (*p* = 0.02)	Quintile, Cox proportional hazards model on workers hired 1945–1949	Cox regression, RR = 1.07(0.87–1.31)	Linear-exponential change deviance = 4.7, β = 0.0016, λ = −0.00041, *p* = 0.09
Limitations	About 60% may have inadequate latency	Exposure bases on air pollution; extrapolate from 1990 transitional DE; low lung cancer risk in referents; low exposure (∼5 μg/m^3^ EC); μg/m^3^ years extrapolated from vehicle miles for non-drivers;	No increased risk among higher exposed shopworkers (E-R not analyzed); Lack of E-R trends; exposure misclassification (no measurements)	E-R slope reduced to 1.02 when adjusted for uranium confounding. Significant lung cancer deficit in referent group;	Incorrect adjustments for smoking and unreliable REC make E-R unreliable
Authors' conclusion	“Limited evidence” entirely dependent on high exposures at Colliery Q	“Positive and significant increase … risk with increasing estimated cumulative exposure” among truck drivers; regard results with “caution” and considered “exploratory”	Evidence supports carcinogenicity of DE; “no evidence of increasing risk” by intensity-years.;	Non-significant E-R support diesel hypothesis	Supports DE hypothesis with “steep increase in risk” at low-moderate levels followed by plateau or decline in risk at high exposures
Conclusion	Does not support diesel hypothesis	Indeterminate	Indeterminate based on lack of E-R trends and lack of alternative hypotheses	Does not support diesel hypothesis; no E-R trend	Indeterminate based on unreliable exposure and biased E-R trends

It's not clear why the lags are having such an effect on the *p* values. But one thing we can be sure about is that excluding the last 15-years of exposure reduces the exposure range by reducing cumulative exposure for all individuals except for retirees living more than 15-years after retirement from the mine. But since latency is adequate in this cohort, the 15-year lags are not needed to assure adequate latency. Lags increase the number of referents with cumulative exposure being reduced to zero. For example, in the unlagged analysis there were 50 workers with cumulative REC exposures >964 µg/m^3^ years; with 15-year lags the exposure range of these same 50 workers has been reduced to >536 µg/m^3^ years. Does this change in the data set result in an artifact in the *p* values?

#### 6.4.5 Inconsistencies in extrapolation of results

The authors’ extrapolation of the study results to public health burden in urban areas is unfortunate. In the last paragraph the authors suggest that the 2–6 μg/m^3^ REC levels in polluted cities is similar to the lower cumulative exposure of UG workers. “Because such workers had at least a 50% increased lung cancer risk, our results suggest that the high air concentrations of elemental carbon in some urban areas may confer increased risk of lung cancer.”

As we have explained, the E-R trends in this study are unreliable and non-linear, and should not be extrapolated to any other population until independent verification can be achieved. We have several questions regarding the authors' suggestion based on their results:

(i) The origin of the 50% increased risk among low exposed UG workers is uncertain.
(ii) ORs of about 1.5 occur in the third quartiles of the unlagged and 15-year lagged models at 246–964 μg/m^3^ years and 72–536 μg/m^3^ years, respectively, for the complete cohort, or at about 350 μg/m^3^ years in the linear-exponential models (Figures 19–20). But these are not low exposures for UG miners.
(iii) At low exposures the ORs are not elevated for the complete cohort, but are elevated in models of UG workers only. In the quartile analysis, the 2nd quartile is the “low” exposure quartile, assuming the 1st quartile is non-exposed. The ORs are 1.45 and 2.46 in the unlagged and lagged models for UG workers. For the complete cohort, however, the ORs are 0.87 (0.48–1.59) and 0.74 (0.40–1.38), and the cumulative RECs are 19–246 and 3–72 μg/m^3^ years for the complete cohort.
(iv) In the expanded categorical 15-year lagged model for the complete cohort, the OR is 0.49 in the lowest exposure category of 20–40 μg/m^3^ years.
(iv) If 2–6 μg/m^3^ REC in highly polluted cities is about the same as the cumulative REC for low exposed UG miners, then there is no apparent 50% increased risk associated with that level of REC exposure in the case-control study. In the categorical analyses of UG miners, the low exposed group is the referent group with ORs set at 1.0, and highest exposures of 81 and 298 μg/m^3^ years are in the quartile analyses for UG workers only. The OR = 1.45 (0.68–3.11) was reported in the 2nd quartile of UG miners with unlagged cumulative exposures 298–675 μg/m^3^ years, which do not amount to low REC exposures.

Johnston et al. (1997) estimated that the average annual ambient exposures to diesel PM amount to a concentration of approximately 2 μg/m^3^. Assuming REC constitutes about 33–90% of diesel particulate carbon (Gamble 2010), Johnson et al. have concluded that 0.7–1.8 µg/m^3^ of REC is unlikely to increase the risk of lung cancer based on their study of DE coal miners.

### 6.6 Summary

In sum, this case-control study suffers from limitations that detract from the authors’ (and editors) conclusion that DE increases the risk of lung cancer. Whether there is an association depends on smoking adjustments. The smoking adjustments appear to be incorrect and producing spuriously increased ORs that are sometimes interpreted as E-R trends. In the absence of a “negative confounding” effect from smoking in the complete cohort of all cases and controls there appears to be no E-R trends. For a risk factor to confound an association it must be associated with REC. The data indicate no apparent association of current smoking and REC, so current smoking is not a confounder. Therefore the smoking adjustment is incorrect, and appears to produce a biased “negative confounding” effect. Crude ORs are suggestive of no association of lung cancer and DE. The reported E-R analyses based on adjusted ORs are considered unreliable and require independent verification and replication before any reliable conclusions are possible regarding lung cancer and DE in this cohort of workers.

Even if the smoking adjustments are not unreliable and do not bias ORs upward, positive results supporting the diesel-lung cancer hypothesis are still inconclusive because they are model dependent; significance depends on which model and lag period are used. There is a statistically significant association of lung cancer and cumulative REC exposure when exposures are lagged 15-years, but no significant associations for unlagged exposures. Model dependency detracts from the diesel hypothesis.

Exposure misclassification is probable and requires independent verification and analysis to determine if the uncertain exposure estimates have biased E-R patterns. Until verification is accomplished study results are considered inadequate for reaching a conclusion.

## 7. Summary of NcI/NIOSH studies of non-metal miners (Attfield et al., 2012; Silverman et al., 2012)

The DEMS studies of this diesel-exposed cohort of nonmetal miners are among the most important epidemiology studies of diesel-exposed workers because of the high and wide range of DE exposures, large sample size, surrogate-based quantitative exposure estimates for E-R analyses, and information on potential workplace and life-style confounders.

After reviewing the DEMS studies (Attfield et al., 2012; Silverman et al., 2012), the guidelines from HEI (Bailar et al., 1999) struck us as having particular relevance to the results of these studies, as follows:

- When specific E-R models are proposed, validation can be accomplished by comparing the fit of the model with general parametric or categorical models. We will use the quartile and expanded categorical models for comparison of the continuous models.
- Were analytical methods specified *a priori*? HEI notes that a study protocol specifies the primary methods of analysis investigators plan to use. “Additional analytical techniques, especially those suggested by the data, can supplement the primary methods of analysis, but they lack full statistical justification. A general concern is that analytical methods not specified *a priori* may be chosen to emphasize some aspect of the findings and, therefore, bias the results.”

As part of the scientific process, “sharing copies of the data and related documentation with colleagues can assist the scientific community and regulatory agencies in understanding the details of a particular study and can provide a scientific ‘second opinion’ that further evaluates the pertinent issues in an objective manner. Sharing data with other investigators for reanalysis can allow other analytic approaches to be developed, which can be particularly important when published studies do not produce a clear consensus about the magnitude, or sometimes even the direction, of an effect.” Our analysis of the epidemiology portion of the NCI/NIOSH studies produced a conclusion contrary to that of the authors. We are not the only “second opinion” that conflicts with the NCI/NIOSH conclusions.

The NCI/NIOSH cohort of non-metal miners is a potentially important addition to the occupational epidemiology database regarding the diesel-lung cancer hypothesis. Major questions remain about smoking adjustments and “negative confounding” effects of smoking, about exposure misclassification and its effect on E-R patterns, about reliance on *a posteriori* analyses and other limitations that require verification and resolution through independent investigators as suggested by Bailar et al., Hopefully these discussions will produce a more definitive answer regarding REC exposure of workers, and from that a more reliable and definitive estimate of the risks potentially associated with exposure to TDE diesel exhaust.

Epidemiology results from the cohort and case-control studies should be confined to consideration of the unlagged and 15-year lagged analyses of the case-control studies. The case-control results are more definitive because adjustments were made for potential confounders including smoking, history of respiratory disease, and history of employment in non-diesel high- risk jobs. Results from the restricted exposure range of <1280 μg/m^3^ years and exclusion of workers with <5-years tenure are considered *a posteriori* results and considered uninformative with regard to the diesel-lung cancer hypothesis.

Overall, we conclude that the results from the nested case-control study are indeterminate with regard to the potential lung carcinogenic effects of REC from TDE in this mining environment. Results are considered indeterminate for several reasons:

- Results are both nominally statistically significant and not significant. Results could be due to chance in the unlagged analyses, but statistically significant in the 15-year lagged analyses. There are no definitive reasons for selecting one model over the other, so the results are model dependent. Model dependent results tend to detract from the diesel-lung cancer hypothesis, or make results indefinite.
- The continuous regression models do not have a conventional E-R pattern. The decline in ORs above about 2000 μg/m^3^ years detracts from asserting a causal mechanism. However, the results are not entirely clear about the risks at these exposure levels. The authors indicate that the E-R slope at these high exposures is a plateau or a decline. The expanded categorical and linear-exponential models are contradictory, especially when cumulative REC exposures are 3000 μg/m^3^ years and greater. Neither a decline nor a plateau in ORs at higher exposures is consistent with typical E-R patterns. E-R is among the strongest evidence supporting a causal association (Hill 1965). A decline or lack of increased risk at the highest exposure levels detracts from the weight of evidence. Perhaps a non-parametric model would allow better understanding of E-R. But if there is no increased risk above 2000 μg/m^3^ years, the argument of no apparent association is strengthened.
- The contrast in exposure data results (Crump, 2012; Borak et al.) and how those differences might change E-R patterns remain unresolved. This conflict may remain unresolved until published results can be independently confirmed. Until then, the results must be viewed as indeterminate.
- We question the basis for concluding that there is a “negative confounding” effect of smoking. Independent verification of the smoking adjustment is necessary before accepting the E-R results of the case-control study.

## 8. Additional cohort studies of truck and bus drivers without estimates of DE exposure

### 8.1. Mortality of truck drivers in trade association (Birdsey et al., 2010)

This is a cohort mortality study of over 150,000 members of a truck driver trade association, 69% of whom were considered drivers. Follow-up was 1989 through 2004 with 3% mortality. There was a deficit in overall mortality with 4,368 cases and an SMR of 0.76 (0.74–0.78). The SMR for lung cancer was 1.00 (0.92–1.09) with 557 observed deaths. Transportation accidents was the only significantly elevated cause of death with an SMR of 1.52 (1.36–1.70).

The authors conclude that the absence of excess mortality may be due to a strong healthy worker effect and a short follow-up period, and that further follow-up is needed. This study contributes little evidence regarding lung cancer and DE. Information on exposure is very limited, and does not include data such as whether a member was an active driver or not, the type of truck, years worked, or age at initial exposure. Contrary to the authors' statement, the healthy worker effect is not expected to be a strong source of bias for diseases with a rapid clinical course such as lung cancer.

### 8.2. Cancer morbidity among Danish bus drivers (Petersen et al., 2010)

This is a 25-year follow-up for cancer of a cohort of 2,037 male Danish urban bus drivers from the three largest cities in Denmark established in 1978 (Netterstrom 1988). There were 540 malignant neoplasms with an overall SIR of 1.09 (1.00–1.20). The increased risks were from lung cancer and bladder cases with 100 and 69 observed cases and Incidence Rate Ratios (IRRs) of 1.2 (1.0–1.4) and 1.6 (1.2–2.0), respectively. Bladder cancer showed a positive but non-significant trend (*p*_trend_ = 0.40) with an IRR of 1.31 (0.70–2.48) in the ≥25-years employed exposure category. Lung cancer showed a non-significant negative E-T trend (*p*_trend_ = 0.79) with IRRs of 1.0, 0.89 (0.59–1.48) and 0.95 (0.55–1.63) in the <15, 15–25-year and ≥25-year exposure groups respectively. Both E-R analyses were adjusted for smoking, city of employment, usual type of bus route, age and calendar time.

Windy weather in these Danish cities tends to attenuate traffic pollution although PM_10_ levels in Copenhagen are compatible with concentrations in London and Paris, but about half those in southern Europe such as Malan and Barcelona. The authors conclude there was no substantial evidence of increased cancer risk from traffic pollution among bus drivers in the Danish cities of Copenhagen, Aarhus and Odense. This is mainly a study of air pollution with only marginal relevance to diesel exposure and partially overlaps with other studies of drivers from Denmark (Soll-Johanning et al., 2003).

### 8.3 Cohort mortality study of bus drivers and bus maintenance workers in Genoa, Italy

This is a cohort mortality study of 9267 male transport workers ever employed 1949–1980 in Genoa, Italy. Follow-up was 1970–2005 with 2916 total deaths and overall SMR of 0.95 (0.92–0.99). There were 386 lung cancer deaths with a significantly increased SMR of 1.23 (1.12–1.36) with the Italian population as the referent. The SMR was reduced to 1.16 (1.05–1.28) with the local Ligurian male population as the referent. There was no apparent E-R slope as SMRs were 2.36 (0.79–1.7) at <9-years, 1.30 (0.98–1.74) at 10–20 years, 1.09 (0.94–1.20) at 20–29 years and 1.21 (1.02–1.43) at ≥30 years employed.

The authors concluded the increased mortality from lung cancer “may be associated” with long-term exposure to PM_10_ and DE air pollution. There was no information on smoking. Length of exposure was the surrogate for exposure thereby “precluding any meaningful direct implication of smoking, particulate matter and diesel fumes as plausible causes.” This study is only marginally relevant to the DE-lung cancer association.

## 9. Updated summary of occupation-based Studies with quantitative estimates of exposure

**Figure 31:**
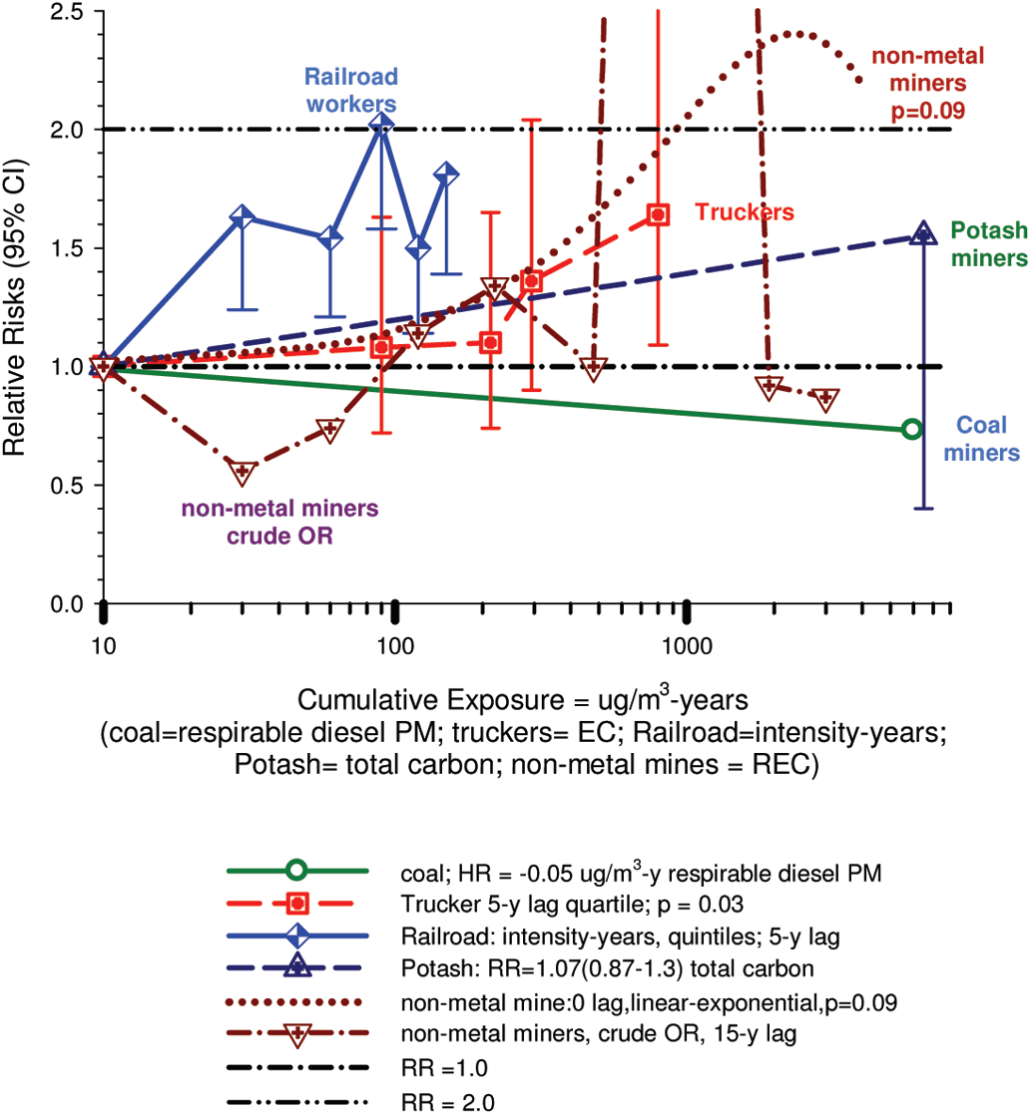
Exposure-response trends of Coal Miners (Johnston et al., 1997), Truckers (Steenland et al., 1998), Railroad engineers/conductors (Laden et al., 2006); Potash miners (unadjusted for uranium confounding) (Neumeyer-Gromen et al., 2009); US nonmetal miners (crude and adjusted ORs) (Silverman et al., 2012).

This section is an overall review of studies having a well-defined cohort of exposed workers with quantitative E-R analyses (Johnston et al., 1997; Steenland et al., 1998; Laden et al., 2006; Neumeyer-Gromen et al., 2009). The overall review is summarized in Figure 31 and Table 9 and is an update of a previous review (Gamble 2010).

Exposure in all studies is to traditional diesel exhaust (TDE) (Hesterberg et al., 2006; Gamble 2010; Hesterberg et al., 2011; Hesterberg et al., 2012) when particulate and gaseous emissions were unregulated and high, and occurred prior to the reductions in emission that began in the 1990s when increasingly stringent regulations began taking effect, resulting in the marked declines in emissions that have continued to the present day (Hesterberg et al., 2011; Hesterberg et al., 2012).

### German potash miners

The potash miners study used a 10-year lag. In the Cox regression adjusted for age, smoking and calendar time, the E-R slope was about 1.07 (0.87–1.31). The tertile E-R slope had a *p*_trend_ of 0.19 and the quintile *p*_trend_ was 0.09. The slope was positive because of an unusually low mortality in the referent group (Gamble 2010).

The only evidence supporting an association is a finding of increasing risk when adjusting for time since first employment, which the authors interpret as a healthy worker effect (HWE), i.e., healthier workers remain in high exposure jobs while sick workers move to less exposed jobs or quit. But the evidence is weak that a HWE operates for lung cancer where the clinical history of disease is short. Further, the evidence cited by the authors is not supportive: a general textbook with no example on lung cancer (Checkoway et al., 2004); a methodological paper with no mention of lung cancer (Steenland et al., 1996); a study of automobile workers where adjustment for duration of work produced slight increased risk estimates in two jobs (Park 1996); and a cohort study where adjustments for duration of employment led to a more dramatic effect on risk estimates, but which the authors warned could be due to residual confounding (Cardis et al., 2007). No mention is made of the large body of evidence from occupational studies of lung cancer showing no effect on risk estimates after adjustments for time since first employment or duration of employment.

Matthias Möhner re-analyzed the potash study and presented his findings on March 24, 2012 at the conference of the German Society for Occupational and Environmental Medicine in Göttingen, Germany. After adjustment for former work in uranium mining, the OR was reduced to a non-significant slope of 1.02 (0.8–1.3) per mg/m^3^ year of cumulative exposure to respirable total carbon. This estimated slope further reduces the non-significant slope of 1.07 (0.87–1.31) from the published Cox regression (Möhner et al., 2012).

The authors suggest strength of this study is that radon is not a relevant confounding variable in potash mining, and cite Short and Petsonk to support that assumption. Radon is not mentioned as an exposure associated with potash mining (Short and Petsonk 1993). The analysis of Mohner et al. (2012) indicates radon from uranium mining is a confounder determined from complete workplace histories among potash miners.

Our interpretation of this study conflicts with the authors of DEMS (Attfield et al., 2012; Silverman et al., 2012). We conclude the German potash miner study does not support the diesel hypothesis because there is no significant E-R trend, and the suggestion of a possible trend is removed by adjustment for radon exposure in uranium mining and the very low lung cancer mortality in the referent group.

### US teamsters cohort

The teamsters study showed similar results and model fits for 5-year lagged and unlagged exposures (Steenland et al., 1998). The quartile and logistic regressions were adjusted for smoking and showed positive slopes with *p*_trend_ values of 0.05 and 0.02 using 1970 emissions of 4.5 g/mile. The log cumulative exposure model slope is 0.18 with a *p*_trend_ of 0.01. Despite exposures being near background air pollution levels and considerably lower than UG miners, the E-R association is stronger than that of UG miners (Figure 31). However, imprecise estimates of dieselization rates undermine the results of this study.

### US railroad workers

In the Railroad cohort, exposure lags of 0, 5, 10 and 15 years were evaluated in models using years worked as the exposure variable. Lags had little effect on results and 5-year lags were used in subsequent analyses based on statistical significance (Laden et al. 2006).

To reduce exposure misclassification, E-R trends are presented for workers hired during 1959–1966 when railroads had completed their conversions from steam to diesel. The risk for any exposure in this group of engineers/conductors was 1.77 (1.50–2.09). There was no evidence of increasing lung cancer risk with increasing years worked or cumulative exposure measured as intensity-years. There were no adjustments for smoking or other potential confounders, which are considered important potential confounders in this group of railroad workers (Crump 1999; Garshick et al., 2006; Gamble 2010). These data do not support the diesel hypothesis.

### UK coal miners

For the coal miner cohort, lags of zero and 15-years are presented, and 20- and 25-year lags sometimes presented. (Johnston et al. 1997). Quantitative estimates of DE exposure were prospective and based on respirable mine dust adjusted for coal and quartz.

Lung cancer SMR overall was 0.86 (0.80–0.93) with the highest SMR of 1.10 (0.88–1.4) in Colliery Q. E-R trends were adjusted for age, smoking and cohort entry data. The 15-year lagged regression had a positive E-R slope of 1.16 (0.90–1.49) that was confounded by mine differences and entirely dependent on Colliery Q, which had higher respirable quartz levels but no internal E-R trend within the colliery. The unlagged model had a negative E-R slope of 0.97 (0.78–1.20).

This is a mining study with quantitative estimates of exposure that the DEMS authors (Attfield et al., 2012; Silverman et al., 2012) overlooked. These study results do not support the diesel hypothesis.

### US non-metal miners (DEMS)

The six studies from the DEMS cohort of non-metal miners exposed to diesel exhaust (Coble et al., 2010; Stewart et al., 2010; Vermeulen et al., 2010a,b; Attfield et al., 2012; Silverman et al., 2012; Stewart et al., 2012) could potentially provide the best epidemiology-based test of the lung cancer-diesel hypothesis when the noted uncertainties are resolved.

Results from the case-control study (Silverman et al, 2012) are considered the most relevant for evaluating the diesel-lung cancer hypothesis and assessing the weight of evidence. These results are adjusted for smoking and other potential confounders. Primary analyses included all cases and controls without exclusion based on tenure or restriction of exposure. The authors' concluded that DE increases the risk of lung cancer with significant E-R trends. The study is large with 198 cases with well-defined time of initial DE exposure and adequate latency.

Exposure assessment results are uncertain, however, and have not been replicable. REC exposure is based on extrapolations from HP to CO to REC where the correlations are low, variable, and not linear based on independent analyses; and the CO data are of questionable precision with such a high proportion of non-detectable samples. Uncertainty in the exposure estimates raises questions about the pattern of E-R trends and detracts from the reliability of reported E-R associations. Exposure assessment results need further analyses and independent confirmation to assure reliability.

Significant E-R associations are found only with 15-year lagged REC cumulative exposure. There are no biological gradients based on crude unadjusted ORs. Adjustments for potential confounding effects of smoking are implausible. Smoking does not appear to be confounder based on the apparent lack of association with REC exposure. Smoking adjustments may be inappropriate based on the authors' citation of inappropriate comparisons of smoking prevalence in high versus low exposed tertiles for UG workers instead of for all cases and controls, and on the implausibly large effects of adjusting for potential confounders. Results are considered indefinite until these questions are resolved.

### Overall weight of evidence

The weight of evidence from these studies is not definitive and is inadequate to conclude that workplace exposure to TDE increases the risk of lung cancer. E-R trends tend to be weakly positive which may be suggestive of causal associations. However, close inspection of these trends indicates potential biases or hidden limitations that complicate interpretation. These include such factors as:

(i) Adjustments for smoking may produce an apparent “negative confounding” effect that biases E-R trends because current smoking was not associated with DE exposure, and therefore was not a confounder (Silverman et al., 2012; Attfield et al., 2012). Thus, confirmation of the ‘true’ relationship by independent investigators is required.
(ii) Sometimes there is a sharp increased risk that remains at the same level even as DE exposure increases. That is, there may be a plateau of increased risk at higher exposures but no apparent E-R trend (Laden and al 2006; Attfield et al., 2012; Silverman et al., 2012).
(iii) In the German potash worker cohort there is a significant overall deficit in lung cancer mortality and the estimated SMR in the referent group is even lower. The observed E-R trend may be due to an inadequate referent group, and it is the unusually low lung cancer mortality rate in that group that produces the trend (Neumeyer-Gromen et al., 2009). Some potash miners had worked in uranium mines, and when this hazardous employment was adjusted for, statistical significance disappeared (Möhner 2012)
(iv) In the UK Study, inclusion of all coal mines showed a statistically significant E-R trend that was produced by one pit that had much higher exposures but only slightly higher mortality. Omission of this pit produced inverse E-R trends. The authors suggest a possible regional effect (Johnston et al., 1997).
(v) One of the strongest E-R trends is among the least biologically plausible workers due to the relatively low DE exposures of Teamsters (Steenland et al., 1998).
(vi) The strength of associations is with RRs less than 2.0 at the highest exposure levels. E-R trends tend to be positive but do not provide consistent or convincing evidence of clear associations with DE exposure because the results could be due to chance or residual confounding when there is a possible trend.

We conclude that the DEMS results are indeterminate because of numerous inconsistencies and unanswered questions. More definitive conclusions must await responses from the authors and independent analyses to address the multiple limitations that have been noted.

Overall, in these occupational cohort studies with the better estimates of DE exposure and adjustments for smoking, the weight of evidence remains inadequate to conclude that there is a causal association between DE exposures and lung cancer. As a result, the epidemiological evidence remains indeterminate regarding the association between traditional diesel exhaust and risks of lung cancer.

## 10. Overall conclusions

The publication of recent meta-analyses, cohort studies, and case-control studies relating to the possible association of occupational exposures to diesel exhaust and an increased incidence of lung cancer has raised the question whether the available epidemiological evidence is different from what the International Agency for Research on Cancer (IARC) determined it to be in 1989 – “limited.” IARC’s conclusion in 1989 (IARC 1989) regarding the limited nature of the available epidemiological data was echoed by the U.S. EPA in its 2002 Health Assessment Document (EPA 2002) and by the Health Effects Institute, both of which noted significant uncertainties in the underlying exposure-response (E-R) relationships, uncertainties that precluded the derivation of any confident quantitative estimate of cancer risk.

This review paper examined in detail the seven recent epidemiology studies that have been published since the data of our prior review (Gamble 2010). Those seven studies are: Birdsey et al., 2010; Merlo et al., 2010; Petersen et al., 2010; Olsson et al., 2011; and Villeneuve et al., 2011 (collectively, the “population and pooled analyses”); and Attfield et al., 2012; and Silverman et al., 2012 (collectively, the “Diesel Exhaust in Miners Study” or “DEMS”). As detailed in our critical review, neither the results of the population and pooled analyses nor the DEMS results (which include a cohort and case-control study) are sufficient to change the conclusion that the available epidemiological data base is inadequate to support a definitive causal association between occupational exposures to diesel engine exhaust and increased risks for lung cancer.

More specifically, the population and pooled analyses suffer from inherent defects in job groupings and exposure estimations, insufficient latency periods, inconsistent *a posteriori* sub-analyses based on cell type, non-significant E-R trends after adjustment for potential cofounders, and failures to adjust for the rates of dieselization or for the evolution of diesel engines and fuels (and thus exposure levels) over time.

The DEMS results are similarly questionable. For the entire cohort, surface workers had higher SMRs than underground miners even though the underground miners' estimated exposures to diesel emissions were 75 times higher than those for surface workers.

Exposure estimates are based on presumed correlations between estimated CO emissions from diesel engines and estimated PM emissions (the marker for respirable elemental carbon). It was further assumed that estimated CO emission levels could be derived from estimates of engine horsepower and mine ventilation rates. None of those assumptions is robust or supported by the available data or literature.

In addition, what many appear to have glossed over is the fact that based on the study’s *a priori* analyses, DEMS cohort was a negative study: **“Initial (i.e., a priori defined) analy-ses from the complete cohort did not reveal a clear rela-tionship of lung cancer mortality with DE exposure**. The hazard ratios (HRs) for the upper three quartiles of cumulative REC exposure were all less than 1.0.” [Bold added.]

Faced with these negative results, the DEMS authors moved to sub-analyses based on worker location. But even then, the results obtained were counter-intuitive. This led to still more sub-analyses of the underground workers only. In those additional analyses, the most significant E-R results were premised entirely on what appear to be unjustified *a posteriori* truncations of the data, including: exposure levels were arbitrarily cut off at 1280 µg/m^3^ year to eliminate an apparent leveling-off or plateauing of any response; a 15-year lag was added to improve the “fit” of the model; an additional minimum 5-year tenure of underground work was added for the highlighted sub-analyses, again to enhance the calculated hazard ratios.

In the case-control study a “negative” confounding effect of smoking was observed in UG workers. Adjustments for purported confounding from smoking in the complete cohort of cases and controls produced a similar “negative confounding” effect to that observed in UG workers. This appears to be an incorrect adjustment for confounding as current smoking is not associated with DE exposure, so smoking cannot be a confounder, and if “confounding” adjustments are made the effect should be negligible. The effect of the unjustified adjustments for current smoking produced spuriously elevated ORs that were incorrectly attributed to DE exposure. The slope of E-R trends using crude ORs are flat, similar to initial results from the cohort study, and are suggestive of inconclusive E-R trends and potentially no association of lung cancer and DE exposure in this study population. Case-control results also do not allow a definitive regarding the association of lung cancer and DE in the DEMS studies.

In sum, the recent publication of new epidemiology studies has not altered the state of the epidemiological data base to the point where the epidemiological data can be deemed sufficient to support a definitive causal association between occupational exposures to diesel engine exhaust and an increased risk of lung cancer. To the contrary, the evidence remains “limited” and inconclusive.

In sum, the evidence is inadequate to adequately test the diesel-lung cancer hypothesis for potential effects of TDE or transitional diesel exhaust on humans.

## Abbreviations

CO_2_: carbon dioxide
CO: carbon monoxide
COD: cause of death
COPD: chronic obstructive pulmonary disease
DE: diesel exhaust
DEMS: diesel exhaust in miners study
DME: diesel motor exhaust
DOC: diesel oxidation catalyst
EM: elemental carbon
E-R: exposure-response
HR: hazard ratio
HEI: Health Effects Institute
IH: industrial hygiene
IARC: International Agency for Research on Cancer
JEM: job exposure matrix
NCI/NIOSH: National Cancer Institute/National Institute of Occupational Safety and Health
NTP: national toxicology program
NO_2_: nitrogen dioxide
NOx: nitrogen oxides
NMRD: nonmalignant respiratory disease
NTDE: non-traditional diesel exhaust
OR: odds ratio
PAHs: polynuclear aromatic hydrocarbons
REC: respirable elemental carbon
SES: socioeconomic status
SMR: standardized mortality ratio
TDE: traditional diesel exhaust
TB: tuberculosis
UG: underground
